# A Molecularly Defined Midbrain Circuit Mechanism for Sound‐Driven Defensive Behavior and Anxiety

**DOI:** 10.1002/advs.77983

**Published:** 2026-09-27

**Authors:** Jie Cao, Xingxing Lai, Lifang Huo, Zhouyuan Zheng, Qian Zhang, Zhimin Ye, Panpan Wan, Chao Ma, Congping Shang

**Affiliations:** ^1^ Guangzhou Medical University Guangzhou China; ^2^ Guangzhou National Laboratory Guangzhou China; ^3^ Department of Rehabilitation Sun Yat‐Sen Memorial Hospital Sun Yat‐Sen University Guangzhou China

**Keywords:** Cerebellin‐2, inferior colliculus, neural circuits, sound‐driven defensive behavior, threatening sound

## Abstract

The innate defense behaviors in response to threatening sounds play a fundamental role in survival. However, the neural circuits that detect threat‐relevant auditory cues and transform them into defensive behaviors remain poorly defined. Here, we identified Cerebellin‐2‐expressing (Cbln2+) neurons work as threatening sound detector in the inferior colliculus shell region (ICx) that were essential for the induction of sound‐driven defensive responses. Cbln2+ ICx neurons specifically responded to threatening sound in a graded manner, and their activation induces escape behavior, autonomic arousal, and fear memory formation. Moreover, this neural subtype provoked sound‐driven defensive and anxiety‐like behaviors through their projection to the dorsal periaqueductal gray (dPAG). Our findings reveal a molecularly defined midbrain circuit that regulates sound‐evoked innate defense and anxiety‐like states primarily through an ICx‐centered subcortical pathway.

## Graphical abstract

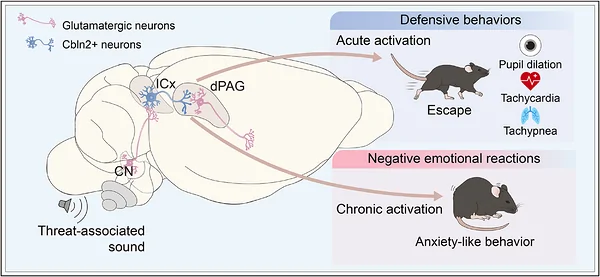

## 1 Introduction

Threat‐relevant sensory stimuli reliably trigger defensive reactions that are essential for survival [1, 2]. Although mammals often rely on hearing to detect danger, the neural mechanisms that transform specific acoustic threat cues into defensive behavior remain less well understood than those underlying visually or olfactorily driven innate defense [3, 4]. As delineated by the classical “predation threat imminence continuum” theory [5], threatening sounds can signal a potential predator during the post‐encounter stage and evoke robust defensive responses. Previous studies have identified multiple neural pathways for sound‐evoked defensive responses. These include brainstem pathways mediating acoustic startle and rapid arousal [6, 7, 8], and top‐down circuits involving the anterior cingulate cortex (ACC) and zona incerta (ZI) that flexibly regulate defensive behavior in a context‐dependent manner [9, 10]. In addition, non‐canonical subcortical pathways, including the inferior colliculus shell region (ICx)–ATm pathway implicated in defensive arousal [11]. Notably, much of the mechanistic work on auditory defense has emphasized cortical or corticofugal pathways, in which the auditory cortex (ACx) can amplify or directly drive defensive responses [12, 13, 14]. Such cortical circuits are well suited for stimulus evaluation, context integration, and associative modulation. By contrast, the auditory midbrain, particularly the ICx, occupies an earlier stage of auditory processing, receives direct ascending input from lower brainstem auditory nuclei [15, 16], suggesting that it may support faster transformation of threat‐related sound cues into defensive actions.

Despite the above discoveries, several key questions remain unresolved. First, it remains unclear whether rapid auditory defense relies primarily on ACx‐dependent descending pathways or on a faster ICx‐centered subcortical neural circuit, and which neuronal populations mediate the detection of threat‐relevant auditory cues and the generation of defensive responses. Second, the synaptic connectivity patterns through which these threat‐detecting neurons connect to downstream brain regions involved in motor execution remain uncharted. Finally, whether persistent overactivation of an auditory defense circuit contributes to anxiety‐related states remains unclear.

In the present study, we used a modified alarm‐call paradigm to evoke defensive behavior in mice. This paradigm enabled quantification of locomotor speed, pupil dilation, tachycardia, tachypnea, and fear‐memory formation. Using this approach, we identify the ICx as a key subcortical node for sound‐evoked defensive responses. We further generated a single‐nucleus transcriptomic atlas of the mouse IC and identified *Cbln2*‐expressing (Cbln2+) neurons in ICx as a critical excitatory neuronal population for sound‐evoked defensive responses. Optogenetic activation of these Cbln2+ ICx neurons was found to trigger robust escape behavior, pupillary dilation, increased heart rate, and accelerated respiration, as well as the formation of fear memories, thus mimicking sound‐evoked defensive states. These neurons responded selectively to threat‐associated acoustic stimuli and initiated defensive responses via axonal projections to the dorsal periaqueductal gray (dPAG). In addition, chronic activation of this circuit induced anxiety‐like behavior in mice. Importantly, repeated 17–20 kHz sound exposure also induced anxiety‐like behavior, which was attenuated by chemogenetic inhibition of the Cbln2+ ICx–dPAG pathway. We further identified the cochlear nucleus (CN)–ICx pathway as an important ascending input for sound‐evoked defense and found that *Cbln2* maintains synaptic efficacy at both the glutamatergic CN–Cbln2+ ICx input and the Cbln2+ ICx–dPAG output. Together, these findings reveal a molecularly defined CN–ICx–dPAG circuit mechanism underlying auditory‐driven escape behavior and anxiety‐like states.

## 2 Results

### 2.1 An Aversive Auditory Stimulus Elicits Innate Escape Behavior

**FIGURE 1:**
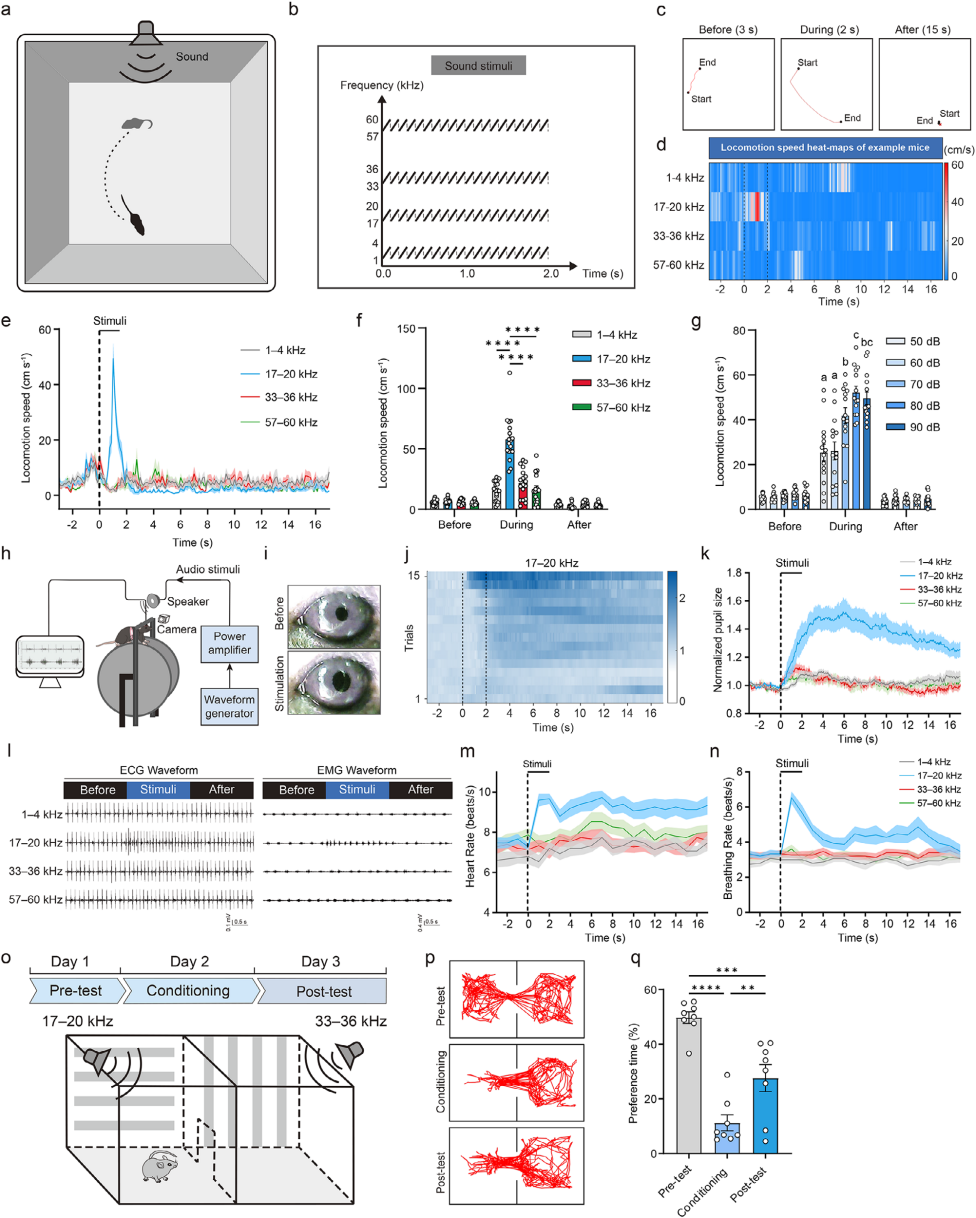
Sound‐evoked innate defensive behavior and autonomic responses. (a) Schematic showing the sound‐evoked escape behavior, in which a speaker was mounted on one wall of the chamber. (b) Schematic of the four upward frequency‐modulated (FM) sweep stimuli. (c) Representative locomotor trajectories of an example mouse before (3 s), during (2 s), and after (15 s) presentation of the 17–20 kHz FM sweep. (d) Heatmaps of speed time course of example mice exposed to the four FM sweep stimuli. (e) Mean locomotor speed traces aligned to stimulus onset for the four FM sweep stimuli (n = 18 mice). (f) Summary of locomotor speed before, during, and after presentation of the four FM sweep stimuli (n = 18 mice). (g) Quantification of locomotor speed before, during, and after sound presentation at different sound intensities (n = 14 mice). (h) Schematic of the setup for simultaneous monitoring of pupil dynamics, respiration, and electrocardiogram (ECG) in awake head‐fixed mice during sound stimulation. (i) Representative pupil images before and during presentation of the 17–20 kHz FM sweep. (j) Heatmaps of pupil dilation bouts in response to the 17–20 kHz FM sweep. (k) Average curves of normalized pupil size of mice with four FM sweep stimuli (n = 5 mice, 15 trials). (l) Representative electrocardiographic (ECG) traces (left) and diaphragm electromyographic (EMG) traces (right) from the awake head‐fixed mice before, during and after four FM sweep stimuli. (m) Average curves of heart rate traces evoked by the four FM sweep stimuli (n = 5 mice, 15 trials). (n) Average curves of breathing rate traces evoked by the four FM sweep stimuli (n = 5 mice, 15 trials). (o) Schematic of the conditioned place aversion (CPA) paradigm. (p) Representative locomotor trajectories during the pre‐test, conditioning, and post‐test phases of the CPA assay. (q) Summarized data from CPA tests (n = 8 mice). All graphs are represented as mean ± SEM, *p < 0.05, **p < 0.01, ***p < 0.001 or ****p < 0.0001. In panel g, bars with different letters differ significantly, whereas bars sharing one or more letters are not significantly different. For detailed statistics information, see Table S4.

To investigate sound‐evoked innate fear, we first screened escape behaviors elicited by four upward frequency‐modulated (FM) sweeps with distinct frequency ranges. Wild‐type (WT) adult male mice were placed in an open field and presented with four sound stimuli (Figure 1a, b, please see supplementary methods and supplementary tables for more information). To quantitatively characterize the escape response, we measured the mean locomotor speed before stimulation (3 s), the peak speed during stimulation (2 s), and the mean speed after stimulation (15 s). Compared with the three control sounds, the 17–20 kHz stimulus markedly increased locomotor speed (Figure 1c–f; Video S1), consistent with previous reports showing that this frequency range effectively elicits defensive responses in rodents [17, 18]. Moreover, the escape speed evoked by the 17–20 kHz stimulus increased with sound intensity and reached a maximum at 80 dB (Figure 1g). Furthermore, we simultaneously monitored autonomic responses, including heart rate, breathing rate, and pupil size, to assess defensive arousal. Mice were head‐fixed and allowed to run freely on a wheel system with controlled ambient luminance (∼60 lx). Following 17–20 kHz sound presentation, mice exhibited rapid pupil dilation (Figure 1i–k and Figure S1a, b), accompanied by sustained increases in both heart rate and breathing rate (Figure 1l–n and Figure S1c, d). Given that loud noise can induce aversion in real‐time place aversion tests, we next examined whether the aversive auditory stimulus could elicit immediate avoidance and produce lasting fear memory. Conditioned place aversion (CPA) tests were performed, in which mice entry into distinct chambers was paired with either the 17–20 kHz or the 33–36 kHz sound stimulus. The results demonstrated that the 17–20 kHz sound successfully induced real‐time place aversion and led to the formation of a conditioned place fear memory (Figure 1o–q). To minimize potential behavioral variability associated with sex, only male mice were used in this study. Collectively, our results demonstrate that the 17–20 kHz sound could reliably induce robust defensive behavior. Thus, we employed this 17–20 kHz sound paradigm to explore the synaptic and circuit mechanisms underpinning the sound‐evoked innate defensive state in mice.

To identify the auditory pathway for sound‐evoked innate fear, we first characterized neuronal activity as reflected in Fos expression in response to 17–20 kHz sound stimulation. Neurons across the auditory pathway showed markedly increased Fos expression (Figure S1e, g), and most Fos‐positive cells were primarily positive for glutamate and negative for GABA (Figure S1f, h). By contrast, silent control group showed low expression level of Fos in the auditory pathway (Figure S1e, g). We next asked which auditory region plays the dominant role in mediating sound‐evoked innate defensive behavior.

### 2.2 Importance of Glutamatergic ICx Neurons in Sound‐Evoked Innate Defensive Behavior

**FIGURE 2:**
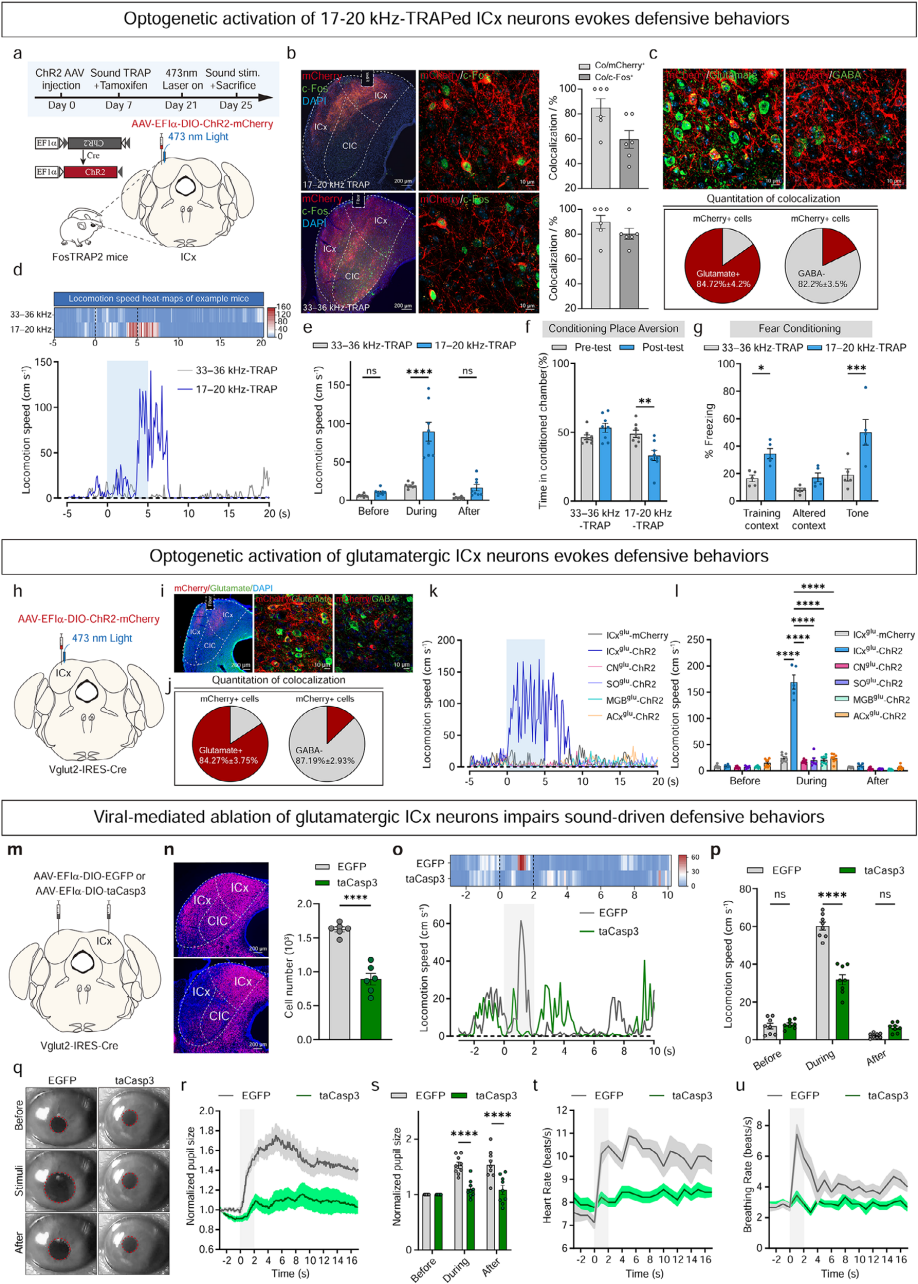
ICx glutamatergic neurons are sufficient and required for sound‐evoked defensive behaviors. (a) Schematic of the FosTRAP procedure and injection of AAV‐EF1α‐DIO‐ChR2‐mCherry into the ICx followed by optical fiber implantation above the ICx of FosTRAP2 mice. (b) Representative micrographs (left) and magnified fields (middle) showing ChR2‐mCherry and Fos expression induced by sound (17–20 kHz or 33–36 kHz)‐TRAPed and sound (17–20 kHz or 33–36 kHz)‐Fos, respectively, together with quantification of the proportion of c‐Fos+/mCherry+ neurons within the mCherry+ population and within the c‐Fos+ population, respectively (right; n = 6 fields from 3 mice per group). (c) Representative images showing that 17–20 kHz‐TRAPed neurons positive for glutamate but negative for GABA (top), together with quantification of the proportions of mCherry+ neurons that were glutamate‐positive or GABA‐negative (bottom; n = 6 fields from 3 mice per group). (d) Example heatmaps of speed time course of photostimulation of 17–20 kHz‐TRAPed and 33–36 kHz‐TRAPed ICx neurons (top) and typical time course of locomotion speed (bottom). (e) Quantitative analyses of locomotion speed before, during, and after optogenetic activation (n = 8 mice per group). (f) Summarized data from CPA tests for optogenetic activation of two sound‐TRAPed ICx neuronal groups (n = 8 mice per group). (g) Quantitative analyses of fear conditioning test after optogenetic activation of sound‐TRAPed ICx neurons (n = 5 mice per group). (h) Schematic of optogenetic activation of Vglut2+ ICx neurons. AAV‐EF1α‐DIO‐ChR2‐mCherry was injected into the ICx of Vglut2‐IRES‐Cre mice and an optical fiber was placed above the ICx. (i) Coronal ICx section showing the expression of ChR2‐mCherry (red) in Vglut2+ ICx neurons and optical fiber tracks in ICx (left). Micrographs showing expression of ChR2 neurons positive for glutamate (middle) but negative for GABA (right). (j) Quantitative analysis of the efficiency and specificity of ChR2 to label Vglut2+ ICx neurons (n = 6 fields from 3 mice). (k) Typical time course of locomotion speed before, during, and after activation of the auditory pathway. (l) Quantification of locomotor speed before, during, and after optogenetic activation of glutamatergic neurons in the indicated auditory regions (n = 8, 5, 7, 6, 8, and 8 mice for the ICxglu‐mCherry, ICxglu‐ChR2, CNglu‐ChR2, SOglu‐ChR2, MGBglu‐ChR2, and ACxglu‐ChR2 groups, respectively). (m) Schematic of virus‐induced apoptosis in Vglut2+ ICx neurons. AAV‐EF1α‐DIO‐taCasp3 was injected into the ICx of Vglut2‐IRES‐Cre mice. AAV‐EF1α‐DIO‐EGFP was injected into the ICx of Vglut2‐IRES‐Cre mice as control. (n) Typical images of taCasp3‐induced apoptosis in Vglut2+ ICx neurons, and summarized data for number of neurons in ICx (n = 6 mice per group). Scale bar, 200 µm. (o) Heatmaps of speed time course of example mice in response to 17–20 kHz stimulation (top) and representative time course of locomotion speed (bottom). (p) Effects of ablation of ICx Vglut2+ neurons on locomotion speed of mice with 17–20 kHz sound‐evoked defensive behavior in Vglut2‐IRES‐Cre mice (n = 8 mice per group). (q) Representative pupil images before, during, and after 17–20 kHz sound stimulation in control and ICx glutamatergic neuron‐ablated mice. (r) Average curves of normalized pupil size. (s) Summarized data of normalized pupil size (right; n = 3 mice, 9 trials per group). (t) Average curves of heart rate. (u) Average curves of breathing rate. All graphs are represented as mean ± SEM, *p < 0.05, **p < 0.01, ***p < 0.001 or ****p < 0.0001. For detailed statistical information, see Table S4.

Given that the ICx has been implicated in innate auditory defense [12], we next used FosTRAP2 to selectively label and optogenetically reactivate ICx neurons activated by 17–20 kHz sound stimulation; neurons activated by 33–36 kHz sound stimulation were labeled as a control population (Figure 2a). Co‐labeling of Fos and mCherry confirmed that the sound‐TRAP procedure labeled the corresponding sound‐responsive ICx ensembles with acceptable specificity and efficiency (Figure 2b). Most neurons within the 17–20 kHz‐TRAPed population were glutamatergic rather than GABAergic (Figure 2c). Strikingly, photostimulation of 17–20 kHz‐TRAPed ICx neurons, but not 33–36 kHz‐TRAPed ICx neurons, robustly increased locomotor speed, induced conditioned place aversion and promoted fear memory formation (Figure 2d–g). To exclude insufficient optical recruitment of ventrally located 33–36 kHz‐TRAPed neurons, we additionally targeted the optical fiber to the CIC and found that activation of this population still failed to induce defensive behaviors (Figure S2a‐e). By contrast, optogenetic activation of Vgat+ ICx neurons failed to evoke obvious defensive behavior (Figure S2f–i). These results suggest that 17–20 kHz‐responsive glutamatergic ICx neurons are sufficient to drive sound‐evoked defensive responses.

We next asked whether glutamatergic neurons across the auditory pathway are generally sufficient to evoke innate defense. ChR2 was expressed in glutamatergic neurons in the ICx, CN, SO, MGB, and ACx, and expression was verified histologically (Figure 2h–j and Figure S2l, m). Optogenetic activation of Vglut2+ neurons in the ICx (473 nm, 10 ms, 20 Hz, 5 s, 20 mW) triggered a vigorous locomotor burst followed by prolonged freezing, whereas activation of glutamatergic neurons in the CN, SO, MGB, or ACx did not elicit comparable defensive responses (Figure 2k, l and Figure S2j, k). Moreover, photostimulation of ICx Vglut2+ neurons was sufficient to induce both CPA and fear memory formation (Figure S2n, o). These findings indicate that ICx glutamatergic neurons are sufficient to evoke innate defensive behavior.

We then tested whether ICx Vglut2+ neurons are required for auditory innate fear. Ablation of these neurons with taCasp3 in Vglut2‐IRES‐Cre mice markedly reduced escape speed evoked by 17–20 kHz sound (Figure 2m–p). Compared with control mice, taCasp3‐treated mice also showed reduced aversion memory, as reflected by increased time spent in the 17–20 kHz‐paired chamber during the CPA assay (Figure S3d). In addition, ablation of ICx Vglut2+ neurons attenuated sound‐evoked pupil dilation and the increases in heart rate and breathing rate (Figure 2q–u, Figure S3a–c). Given that previous studies have demonstrated that ACx neurons projecting to the ICx mediate broadband noise‐evoked escape behavior [12], we also examined the role of ACx glutamatergic neurons. Ablation of glutamatergic neurons in the ACx significantly reduced freezing to the conditioned tone (Figure S3h), but did not significantly impair the escape speed induced by 17–20 kHz sound (Figure S3f, g). These data suggest that 17–20 kHz‐evoked escape behavior is largely independent of ACx‐dependent descending control.

Finally, we next expressed GCaMP6s in ICx Vglut2+ neurons and recorded population activity with fiber photometry. Presentation of 17–20 kHz sound elicited a pronounced increase in GCaMP fluorescence in ICx Vglut2+ neurons. Notably, Vglut2+ ICx neurons also exhibited increased GCaMP fluorescence in response to the other three control sounds (Figure S3i–m). These findings suggest that the Vglut2+ ICx population is functionally heterogeneous. This observation prompted us to ask whether a more specific neuronal subtype within ICx glutamatergic neurons selectively detects and mediates the defensive responses evoked by 17–20 kHz sound.

### 2.3 Single‐Nucleus Transcriptomic Atlas of IC

**FIGURE 3:**
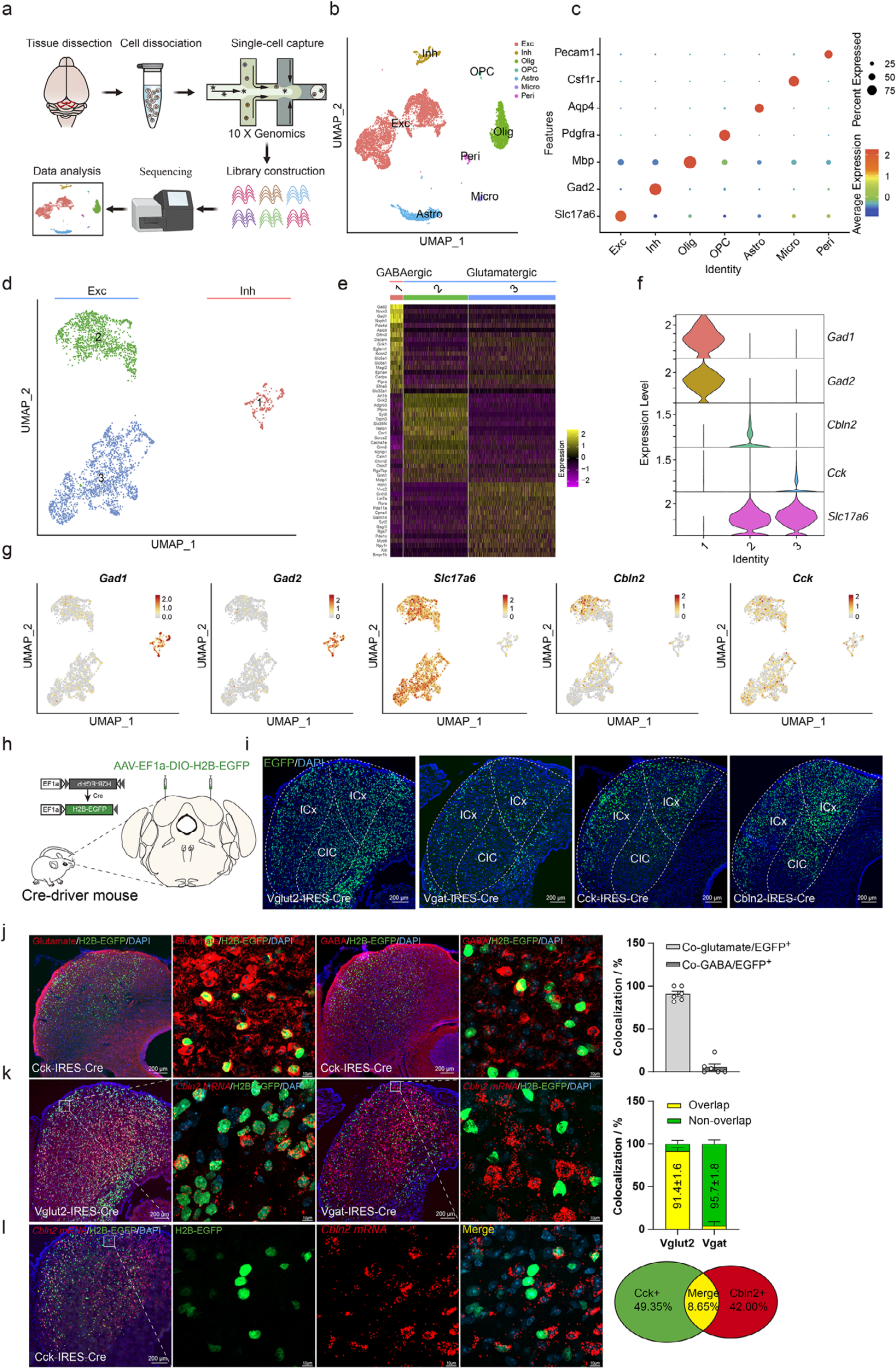
Single‐nucleus transcriptomic identification and histological validation of neuronal subtypes in the IC. (a) Schematic diagram showing the brain tissues containing the IC were micro‐dissected for snRNA‐seq. (b) UMAP visualization of all IC cell classes, including excitatory neurons (Exc), inhibitory neurons (Inh), oligodendrocytes (Olig), oligodendrocyte precursor cells (OPC), astrocytes (Astro), microglia (Micro), and pericytes (Peri). (c) Dot plot showing representative marker genes across major IC cell classes. Dot size indicates the percentage of cells expressing each gene, and color indicates average expression level.Expression values are log‐normalized counts. (d) UMAP visualization of neuronal clusters, showing inhibitory cluster (cluster 1) and excitatory clusters (clusters 2 and 3). (e) Heatmap of representative marker genes across the three neuronal clusters. (f) Violin plots showing the expression of Gad1, Gad2, Cbln2, Cck, and Slc17a6 across neuronal clusters. (g) Feature plots showing the expression patterns of Gad1, Gad2, Slc17a6, Cbln2, and Cck in neuronal clusters. Expression values are log‐normalized counts. (h) Diagram showing AAV‐EF1α‐DIO‐H2B‐EGFP injection into the IC of Cre‐line mice. (i) Example coronal brain section showing expression of H2B‐EGFP in the IC of Cre‐line mice. (j) Example coronal brain section and magnified field from Cck‐IRES‐Cre mice showing colocalization of H2B‐EGFP+ neurons with glutamate (left) or GABA (right) immunostaining, together with quantification of colocalization (n = 6 fields from 3 mice). (k) Example coronal brain section and magnified field showing co‐localization of Cbln2 mRNA with H2B‐EGFP+ neurons in Vglut2‐IRES‐Cre and Vgat‐IRES‐Cre mice, together with quantification of colocalization (n = 6 fields from 3 mice). (l) Example coronal brain section and magnified field showing co‐localization of Cbln2 mRNA with H2B‐EGFP+ neurons in Cck‐IRES‐Cre mice, together with a summary diagram showing limited overlap between Cck+ and Cbln2+ neuronal populations (n = 9 fields from 3 mice). Scale bars, 200 µm in low‐magnification images and 10 µm in high‐magnification images.

Because the IC contains a large and heterogeneous neuronal population, overlap among distinct cell types may complicate the identification of functionally relevant neuronal subtypes based solely on morphology or physiological properties [16]. We therefore examined cellular diversity in the IC using single‐nucleus RNA sequencing (snRNA‐seq) [19] with the 10x Genomics Chromium platform [20] (Figure 3a). After quality control, we obtained 7549 cells from WT mice across two biological replicates (5 mice in total). These cells were clustered using graph‐based analysis in Seurat with batch correction performed by Harmony, and cell identities were assigned on the basis of canonical marker genes (Figure 3b, c) [21, 22]. Three neuronal clusters were identified in the IC: cluster‐1 was GABAergic, whereas clusters 2 and 3 were glutamatergic (Figure 3d). We next identified signature genes for each cluster, including neurotransmitter‐related genes, transcription factor‐related genes, and ion channels (Figure 3e). Notably, *Cbln2* and *Cck* selectively marked the two excitatory clusters, nominating them as candidate markers of molecularly distinct glutamatergic IC subtypes (Figure 3f, g).

To validate the expression specificity of these markers in vivo, we injected AAV‐DIO‐H2B‐EGFP into the IC of the corresponding Cre driver lines. The results demonstrated that EGFP‐labeled Vglut2+, Vgat+, Cbln2+ and Cck+ neurons were distributed throughout the IC (Figure 3h, i). Histological analysis further showed that Cck+ neurons were predominantly glutamatergic and rarely GABAergic (Figure 3j). In addition, Cbln2+ neurons largely overlapped with the glutamatergic population labeled in Vglut2‐IRES‐Cre mice (Figure 3k). By contrast, Cbln2+ and Cck+ neurons exhibited only limited overlap (8.65%±1.89%, n = 4 mice), indicating that they represent two molecularly distinct excitatory IC populations (Figure 3l). These results establish Cbln2+ and Cck+ neurons as genetically accessible glutamatergic IC subtypes for subsequent functional dissection of sound‐evoked defensive behavior.

### 2.4 Cbln2+ ICx Neurons are Essential for Sound‐Evoked Defensive Responses

**FIGURE 4:**
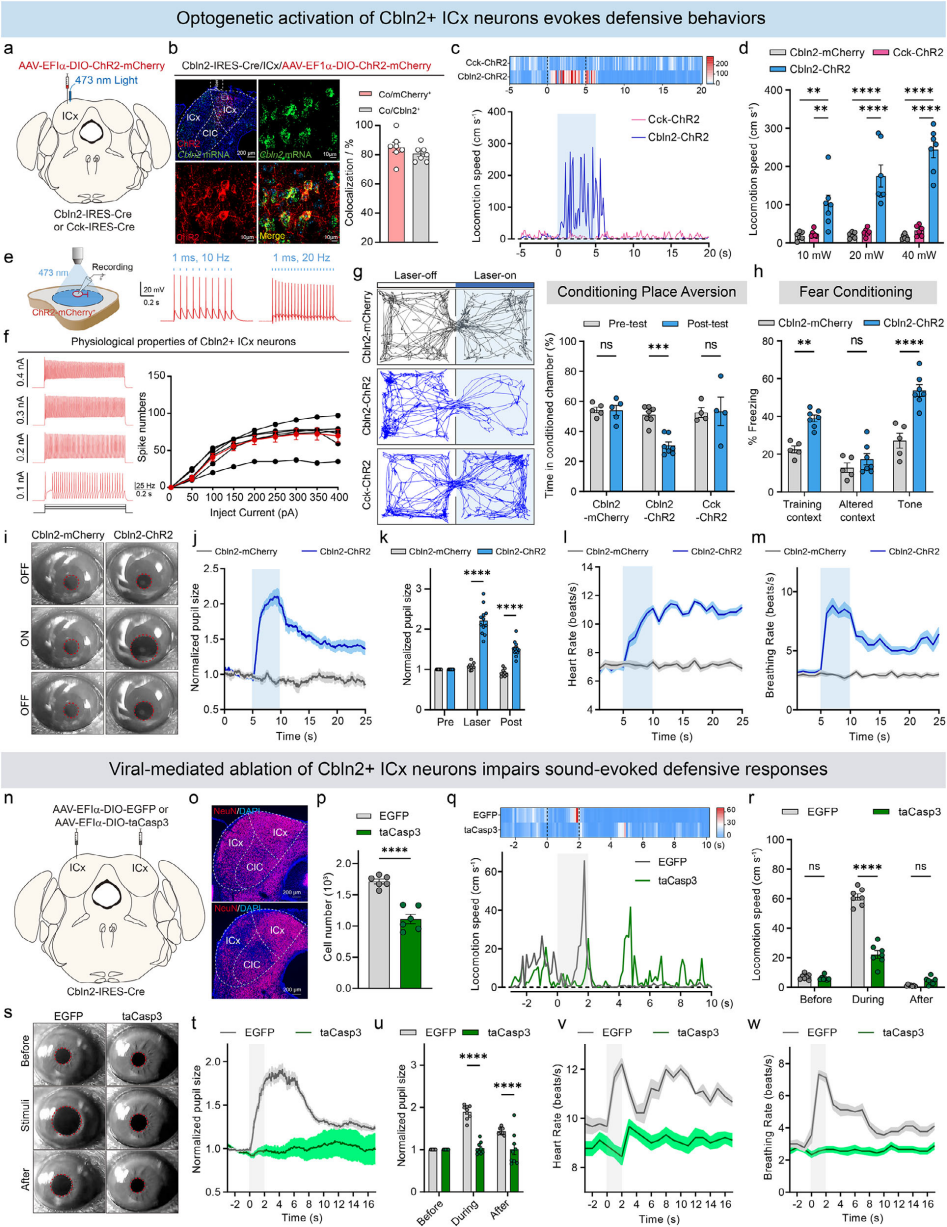
Cbln2+ ICx neurons are sufficient and required for sound‐evoked defensive and autonomic responses. (a) Schematic of optogenetic activation of Cbln2+ ICx neurons. AAV‐EF1α‐DIO‐ChR2‐mCherry was injected into the ICx of Cbln2‐IRES‐Cre mice and optical fiber was placed above the ICx. (b) Representative coronal section and higher‐magnification images showing ChR2‐mCherry expression in ICx Cbln2+ neurons and colocalization of ChR2‐mCherry with Cbln2 mRNA signals (left), quantification of the specificity and efficiency of ChR2‐mCherry labeling in Cbln2+ ICx neurons (right; n = 7 fields from 3 mice). (c) Representative heat maps of locomotor speed (top) and mean locomotor speed traces (bottom) during photostimulation in Cck‐ChR2 and Cbln2‐ChR2 mice. (d) Quantitative analyses of peak locomotion speed of Cck‐IRES‐Cre or Cbln2‐IRES‐Cre mice during different laser power activation (n = 6, 6, and 7 mice for Cbln2‐mCherry, Cck‐ChR2 and Cbln2‐ChR2 groups, respectively). (e) Schematic of acute slice recording (left), and representative light‐evoked spiking in ChR2‐expressing Cbln2+ ICx neurons during 10 Hz and 20 Hz photostimulation (right). (f) Electrophysiological properties of Cbln2+ ICx neurons, including representative firing patterns evoked by depolarizing current injections (left) and quantitative analyses of spike number as a function of current intensity (right; n = 6 cells). (g) Representative trajectories in the CPA test (left), quantification of time spent in the conditioned chamber during the pre‐test and post‐test sessions (right; n = 5, 4, 7 mice for Cbln2‐mCherry, Cck‐ChR2 and Cbln2‐ChR2 groups, respectively). (h) Quantification of freezing during fear conditioning (n = 5 mice for Cbln2‐mCherry and n = 7 mice for Cbln2‐ChR2). (i) Representative pupil images before and during photostimulation in Cbln2‐mCherry and Cbln2‐ChR2 mice. (j) Average curves of normalized pupil size. (k) Summarized data of normalized pupil size (n = 3 mice, 9 trials for Cbln2‐mCherry and n = 4 mice, 12 trials for Cbln2‐ChR2). (l) Average curves of heart rate. (m) Average curves of breathing rate. (n) Schematic of virus‐induced apoptosis in Cbln2+ ICx neurons. (o) Typical images of taCasp3‐induced apoptosis in Cbln2+ ICx neurons, Scale bar, 200 µm. (p) Quantification of surviving cells in ICx (right; n = 6 mice per group). (q) Heatmaps of speed time course of example mice in response to 17–20 kHz sound stimulation (top) and representative time course of locomotion speed (bottom). (r) Quantification of locomotor speed before, during, and after 17–20 kHz sound stimulation in control and Cbln2+ ICx neuron‐ablated mice (n = 7 mice per group). (s) Representative pupil images before, during, and after 17–20 kHz sound stimulation in control and Cbln2+ ICx neuron‐ablated mice. (t) Average curves of normalized pupil size. (u) Summarized data of normalized pupil size (n = 3 mice, 9 Trials per group). (v) Average curves of heart rate. (w) Average curves of breathing rate. All graphs are represented as mean ± SEM, *p < 0.05, **p < 0.01, ***p < 0.001 or ****p < 0.0001. For detailed statistical information, see Table S4.

We next asked whether the two molecularly defined excitatory ICx populations, Cbln2+ and Cck+ neurons, differentially control sound‐evoked innate fear. To this end, we expressed ChR2‐mCherry in the ICx of Cbln2‐IRES‐Cre and Cck‐IRES‐Cre mice and implanted optical fibers above the ICx (Figure 4a, b and Figure S4o). In acute slices, light pulses reliably evoked APs in ChR2‐expressing Cbln2+ ICx neurons (Figure 4e). Photostimulation of Cbln2+ ICx neurons induced a robust locomotor burst. However, no such escape responses were elicited upon the photoactivation of Cck+ ICx neurons (Figure 4c, d; Videos S2 and S3). The speed of light‐evoked escape depended on the duration and frequency of photostimulation, but did not decrease across repeated stimulation bouts (Figure S4a–c). Consistent with this difference, activation of Cbln2+ but not Cck+ ICx neurons induced fear memory formation (Figure 4g). Moreover, Cbln2+ ICx activation triggered significant pupil dilation and increases in heart rate and breathing rate, whereas Cck+ ICx activation did not produce comparable autonomic responses (Figure 4i–m and Figure S4d–n). Together, these data indicate that activation of Cbln2+ ICx neurons is sufficient to drive innate fear‐like defensive responses.

To test whether Cbln2+ ICx neurons are required for sound‐evoked innate fear, we injected AAV vector encoding taCasp3 to ICx induce Cbln2+ neurons lesion (Figure 4n–p). Ablation of Cbln2+ ICx neurons markedly reduced 17–20 kHz sound‐evoked defensive responses, as evidenced by reduced escape speed (Figure 4q, r), the absence of avoidance behavior, and the failure to formation conditioned place fear memory (Figure S5d). Furthermore, no significant alterations were observed in autonomic responses, including pupil diameter, heart rate, and breathing rate (Figure 4s–w and Figure S5a–c). By contrast, such effects were not observed following the ablation of Cck+ ICx neurons (Figure S5e–r). These results therefore demonstrate that Cbln2+ ICx neurons are required for sound‐evoked innate fear.

We next validated the importance of *Cbln2* itself for 17–20 kHz‐evoked defensive responses. We knocked down *Cbln2* in the ICx of wild‐type mice using an AAV expressing an H1‐driven shRNA against *Cbln2* (Figure S6a). This manipulation efficiently reduced *Cbln2* expression, as confirmed by histology (Figure S6a, b). Then, we examined defensive responses to 17–20 kHz sound stimulation in *Cbln2*‐knockdown (*Cbln2*‐KD) and negative control (NC) mice. Compared with NC mice, *Cbln2*‐KD mice showed markedly attenuated defensive responses, including reduced escape speed (Figure S6c, d), diminished conditioned place aversion (Figure S6e) and autonomic responses (Figure S6f–j). Together, these results indicate that *Cbln2* expression in the ICx is required for the full expression of 17–20 kHz‐evoked innate fear responses.

### 2.5 Cbln2+ ICx Neurons Specifically Detect Threat‐Relevant Auditory Signals

Next, we systematically characterized the physiological properties of Cbln2*+* ICx neurons. AAV‐DIO‐EGFP was injected into ICx of Cbln2‐IRES‐Cre mice. Whole‐cell recording from the EGFP+ ICx neurons in acute slices revealed spiking activity with a slow adaptive pattern in response to depolarizing current injections (Figure 4f).

**FIGURE 5:**
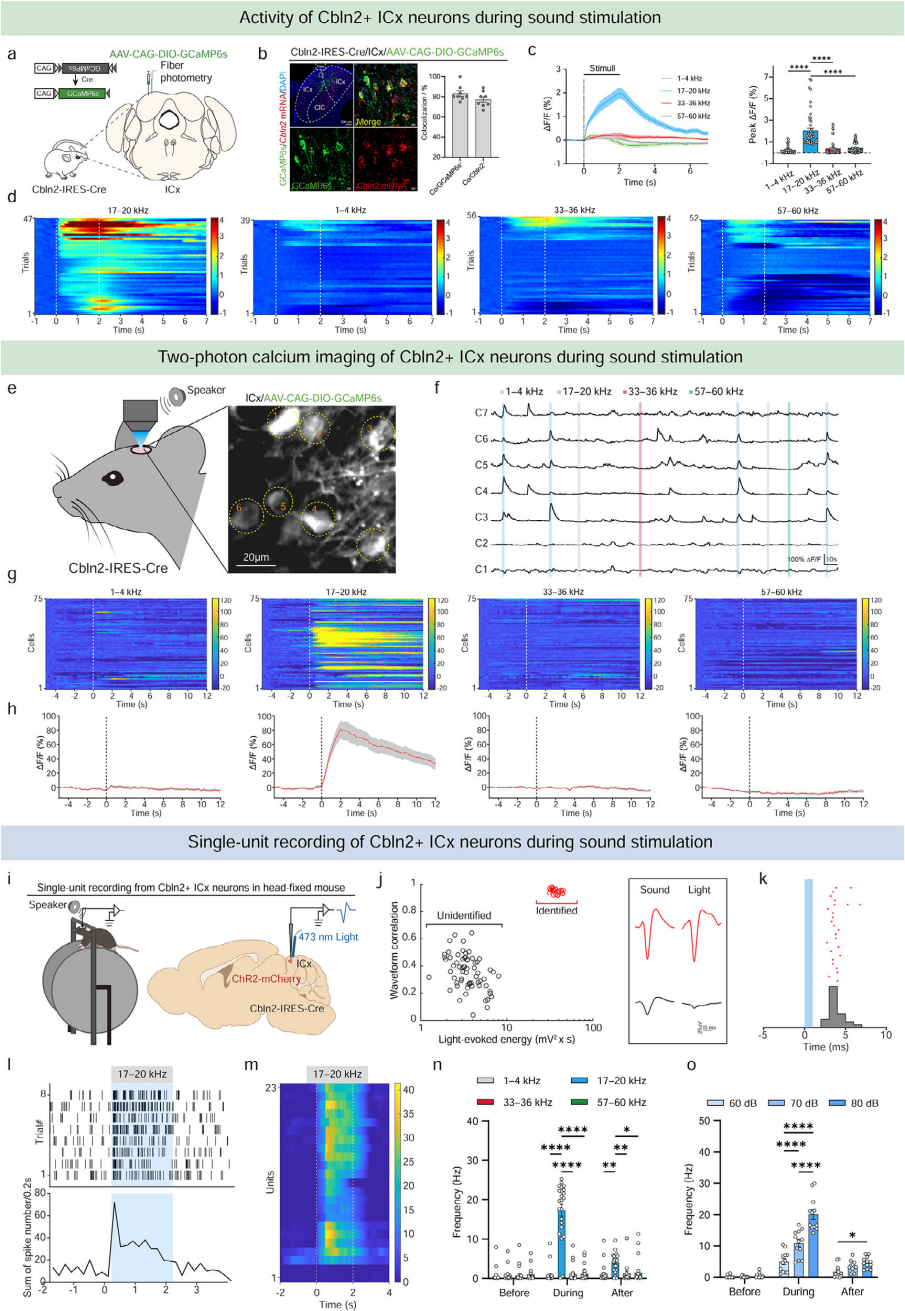
Activity of Cbln2+ ICx neurons during sound stimulation. (a) Schematic of fiber photometry recording from Cbln2+ ICx neurons. AAV‐CAG‐DIO‐GCaMP6s was injected into the ICx of Cbln2‐IRES‐Cre mice, followed by optical fiber implantation above the ICx. (b) Example coronal section showing optical fiber track above GCaMP6s‐expressing neurons in ICx of Cbln2‐IRES‐Cre mice. Scale bar, 200 µm. Micrographs showing specific expression of GCaMP6s in Cbln2+ ICx neurons. Scale bar, 10 µm. Together with quantification of the specificity and efficiency of GCaMP6s labeling (n = 8 fields from 4 mice). (c) Average GCaMP response curve (left) and quantification of peak ΔF/F responses in Cbln2+ ICx neurons evoked by the indicated sound stimuli (right; n = 6 mice). (d) Heatmaps of Ca2+ activity of Cbln2+ ICx neurons to the indicated sound stimuli. (e) Schematic of in vivo calcium imaging in head‐fixed Cbln2‐IRES‐Cre mice during sound presentation, together with a representative imaging field showing identified neurons. (f) Representative calcium traces from individual Cbln2+ ICx neurons during presentation of the indicated sound stimuli. (g) Heatmaps showing sound‐evoked calcium responses to the indicated sound stimuli (n = 75 cells from 4 mice). (h) Mean single‐neuron calcium responses to the indicated sound stimuli. (i) Schematic diagram showing a head‐fixed awake mouse walking on a cylindrical treadmill (left) and antidromic activation strategy for single‐unit recording from Cbln2+ ICx neurons (right). (j) Correlation analysis of action potentials of individual units evoked either by light pulses (Light) or by threatening sound (sound), confirming a segregation between antidromically identified units (red dots and traces) and unidentified units (black dots and traces). (k) Raster (top) and peristimulus time histogram (PSTH, bottom) of putative Cbln2+ ICx neurons showing a latency of ∼3 ms relative to the onset of light pulses (n = 23 units from 4 mice). (l) Raster plots from an example putative Cbln2+ ICx neuron in response to 17–20 kHz sound stimulation (top; n = 8 trials), together with the summed spike counts across trials (bottom). (m) Heat map of firing responses from optotagged Cbln2+ ICx neurons to 17–20 kHz sound stimulation (bottom; n = 23 units). (n) Mean firing frequency from ChR2‐tagged neurons in response to the indicated sound stimulation (n = 23 units). (o) Mean firing frequency from ChR2‐tagged neurons in response to different intensities of 17–20 kHz sound stimulation (n = 12 units). All graphs are represented as mean ± SEM, *p < 0.05, **p < 0.01, ***p < 0.001 or ****p < 0.0001. For detailed statistical information, see Table S4.

To further define the response selectivity of Cbln2+ ICx neurons to auditory stimuli, we recorded GCaMP signals in Cbln2+ ICx neurons using fiber photometry. AAV‐DIO‐GCaMP6s was injected into ICx of Cbln2‐IRES‐Cre mice, followed by implantation of an optical fiber above the Cbln2+ ICx neurons. We observed localized and specific expression of GCaMP6s in Cbln2+ ICx neurons (Figure 5a, b). The calcium signaling responses of Cbln2+ ICx neurons were examined following exposure to 17–20 kHz sound and other three control sounds (1–4, 33–36, and 57–60 kHz). We observed that the 17–20 kHz stimuli elicited a significant increase in GCaMP fluorescence intensity (Video S4), whereas control sounds produced little or no response (Figure 5c, d). Additionally, the GCaMP fluorescence responses of Cck+ ICx neurons to various sound stimuli were evaluated. Intriguingly, both 57–60 kHz and 33–36 kHz stimuli elicited significant increases in GCaMP responses. By contrast, the 17–20 kHz sound induced only a weak transient response followed by suppression, and 1–4 kHz sound produced a more pronounced decrease in fluorescence (Figure S7a–d). These findings suggest that Cbln2+ ICx neurons exhibit a marked preference for the 17–20 kHz threat‐associated sound.

Since fiber photometry does not provide information about the activity of individual neurons, we next performed in vivo two‐photon Ca2+ imaging experiments in Cbln2‐IRES‐Cre mice delivered with AAV‐DIO‐GCaMP6s virus to ICx (Figure 5e). Two weeks post‐injection, we recorded neuronal responses to various sound stimuli. Cbln2+ ICx neurons showed a significant, time‐locked increase in GCaMP signals upon 17–20 kHz sound, whereas responses to the three control sounds were weak or absent (1–4, 33–36, and 57–60 kHz). When the GCaMP signals of all recorded Cbln2+ ICx neurons were averaged, the peak GCaMP signal reached ∼80% during 17–20 kHz stimulation (Figure 5g,h). These results indicate that the selective response of Cbln2+ ICx neurons to threat‐associated sound is already evident at single‐neuron resolution.

To achieve better temporal resolution of Cbln2+ ICx neuron activity in response to various sound stimuli, we performed in vivo optrode recordings in head‐fixed awake mice. AAV‐DIO‐ChR2‐mCherry was injected into the ICx of Cbln2‐IRES‐Cre mice. The putative Cbln2+ ICx neurons were identified according to the action potentials (APs) evoked by light pulses (473 nm, 1 ms, 5 mW) illuminating on ChR2‐mCherry+ ICx neurons. The light‐evoked APs were required to meet two stringent criteria: a) response latencies to light pulses should be less than 5 ms; b) light‐evoked waveforms should be similar to spontaneous spikes. We identified 23 units from four mice as putative Cbln2+ ICx neurons according to the two criteria (Figure 5i–k). Next, we examined the responses of these neurons to auditory stimuli. The results showed that these neurons responded to 17–20 kHz stimulation in a graded manner (Figure 5o and Figure S8d, e), while showing little or no response to several control stimuli (Figure 5l–n and Figure S8a–c). Together, the fiber photometry, two‐photon imaging, and optotagged single‐unit recordings consistently indicate that Cbln2+ ICx neurons are preferentially tuned to 17–20 kHz threat‐associated sound stimuli.

### 2.6 The Cbln2+ ICx–dPAG Pathway Mediates Sound‐Driven Defensive Behavior

**FIGURE 6:**
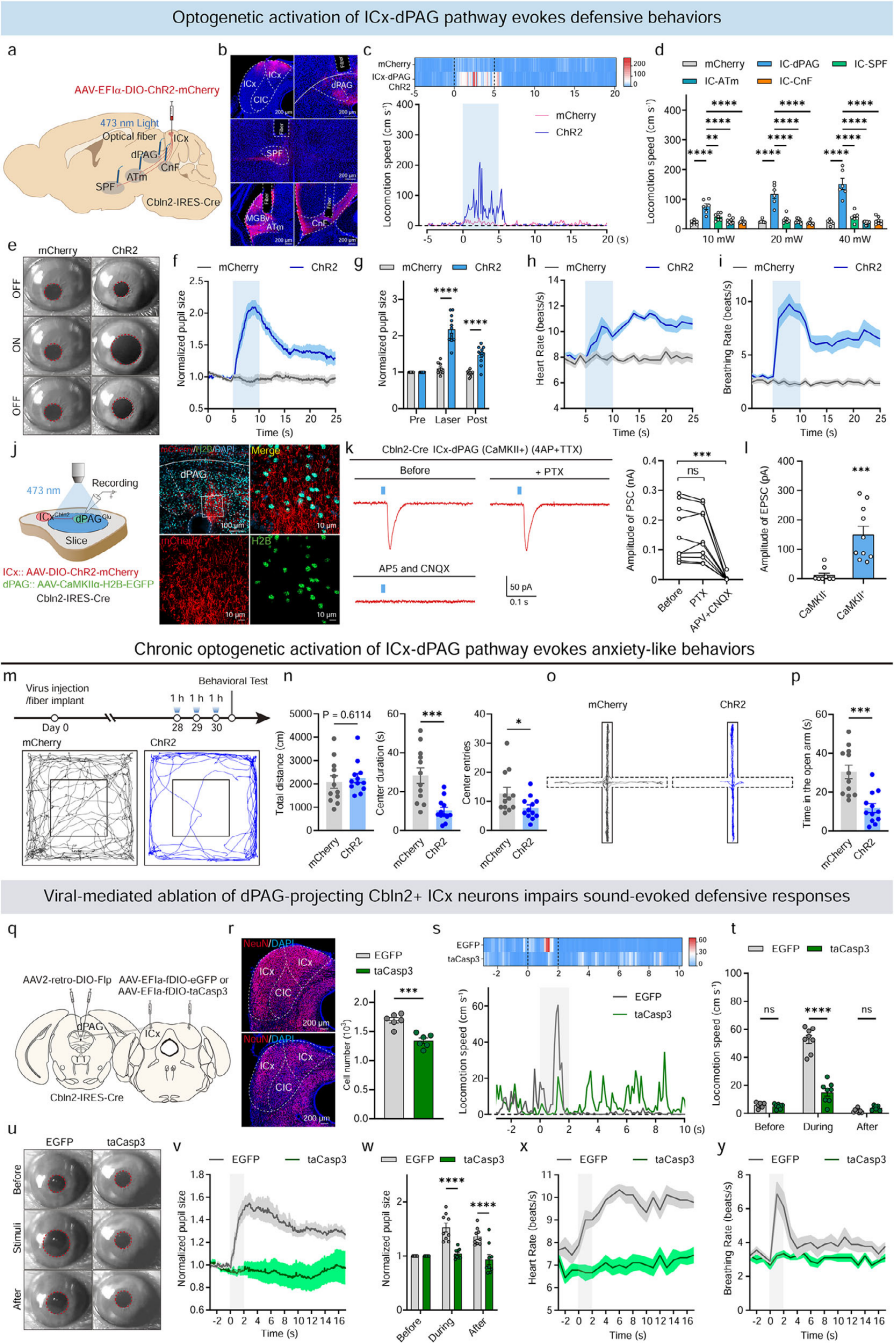
The Cbln2+ ICx‐dPAG pathway is sufficient and required for sound‐evoked defensive and autonomic responses, and repeated activation induces anxiety‐like behaviors. (a) Schematic of optogenetic activation of the Cbln2+ ICx‐dPAG, ICx‐CnF, ICx‐ATm or ICx‐SPF pathway. AAV‐DIO‐ChR2‐mCherry was injected into the ICx of Cbln2‐IRES‐Cre mice and optical fiber was placed above the dPAG, CnF, ATm or SPF. (b) Coronal ICx section showing the expression of ChR2‐mCherry in ICx and optical fiber tracks above ChR2‐mCherry+ axonal projections of Cbln2+ ICx neurons in dPAG, CnF, ATm or SPF. (c) Example heatmaps of speed time course of optogenetic activation of Cbln2+ ICx‐dPAG pathway (top) and typical time course of locomotion speed (bottom). (d) Quantification of peak locomotor speed during photostimulation of Cbln2+ ICx terminals in the indicated projection targets at different laser powers (n = 6, 6, 7, 8, and 8 mice for the Cbln2‐mCherry ICx–dPAG control, and the Cbln2‐ChR2 ICx–dPAG, ICx–CnF, ICx–ATm, and ICx–SPF projection groups, respectively). (e) Typical images of pupil of activation of Cbln2+ ICx‐dPAG pathway. (f) Average curves of normalized pupil size. (g) Summarized data of normalized pupil size (n = 3 mice, 9 Trials for mCherry and n = 4 mice, 12 Trials for ChR2). (h) Average curves of heart rate. (i) Average curves of breathing rate. (j) Schematic diagram showing virus injection and recording configuration in acute slices of Cbln2‐IRES‐Cre mice (left). Example coronal brain section and magnified area (right) showing mCherry+ fibers in dPAG surrounding EGFP+ glutamatergic neurons. Scale bar, 200 µm; inset, 10 µm. (k) Example traces (left) and summarized data (right) showing effects of picrotoxin (PTX, 50 µM) and APV (50 µM)/CNQX (20 µM) on postsynaptic currents recorded from dPAG EGFP+ glutamatergic neurons in acute slices in response to light‐stimulation of ICx‐dPAG circuits (n = 10 cells). (l) Quantitative analyses showing the amplitude of light‐evoked excitatory postsynaptic currents (EPSCs) recorded from EGFP‐negative (n = 8 cells) and EGFP+ neurons (n = 10 cells) in the dPAG. (m) Experimental procedure to examine if repeated activations of Cbln2+ ICx‐dPAG pathway caused anxiety‐like behaviors (top), representative trajectory chart in the open field test (bottom). (n) Quantitative analyses of total distance, Center duration and center entries in the open field test (n = 12 mice per group). (o) Representative traces in the elevated plus maze (EPM). (p) Quantitative analyses of time in open arm (n = 12 mice per group). (q) Schematic of pathway‐specific ablation of dPAG‐projecting Cbln2+ ICx neurons. (r) Typical images of taCasp3‐induced apoptosis in dPAG‐projecting Cbln2+ ICx neurons (left), and quantification of surviving cells. Scale bar, 200 µm (right; n = 6 mice for EGFP and taCasp3 groups, respectively). (s) Heatmaps of speed time course of example mice in response to 17–20 kHz sound stimulation (top) and representative time course of locomotion speed (bottom). (t) Quantification of locomotor speed before, during, and after 17–20 kHz sound stimulation (n = 8 mice per group). (u) Typical pupil images following ablation of Cbln2+ ICx‐dPAG pathway in response to 17–20 kHz sound stimulation. (v) Average curves of normalized pupil size. (w) Summarized data of normalized pupil size (bottom; n = 3 mice, 9 Trials per group). (x) Average curves of heart rate. (y) Average curves of breathing rate. All graphs are represented as mean ± SEM, *p < 0.05, **p < 0.01, ***p < 0.001 or ****p < 0.0001. For detailed statistical information, see Table S4.

Having identified the Cbln2+ ICx neurons, but not Cck+ ICx neurons, as a key excitatory population for auditory‐driven innate fear, we compared the output patterns of Cbln2+ and Cck+ ICx neurons by anterograde tracing. AAV‐DIO‐H2B‐EGFP‐synaptophysin‐mRuby was injected into the ICx of Cbln2‐IRES‐Cre and Cck‐IRES‐Cre mice (Figure S9a–f). The anterogradely tracing results implied that differences in downstream projection targets between Cbln2+ neurons and Cck+ neurons might be responsible for driving distinct behavioral outputs. As shown in Figure S8b, Cbln2+ ICx neurons projected broadly to multiple downstream targets, including auditory‐associated regions, CnF, ATm, the dorsal periaqueductal gray (dPAG), and the subparafascicular thalamic nucleus (SPF). Previous studies have shown that neural populations in the CnF, ATm, dPAG, and SPF are involved in distinct aspects of defensive processing, including escape control, defensive arousal, and aversive‐state coding [11, 23, 24, 25, 26]. We therefore tested whether stimulation of these projection‐defined pathways is sufficient to evoke defensive behavior. AAV‐DIO‐ChR2‐mCherry was injected into the ICx of Cbln2‐IRES‐Cre mice, followed by optical fiber implantation in dPAG, ATm, CnF or SPF. Terminal photostimulation in the dPAG robustly increased locomotor speed (Video S5), whereas stimulation in SPF, ATm or CnF failed to evoke comparable escape responses (Figure 6a–d). Moreover, the magnitude of the dPAG terminal‐evoked escape response scaled with photostimulation power, duration, and frequency, but remained stable across repeated stimulation bouts (Figure 6d and Figure S10a–c). Thus, among the major downstream targets tested, the Cbln2+ ICx–dPAG projection was uniquely sufficient to drive robust escape behavior.

To further investigate the synaptic properties of this pathway, we next injected Cbln2‐IRES‐Cre mice with AAV‐DIO‐ChR2‐mCherry into the ICx and AAV‐CaMKIIα‐H2B‐EGFP into the dPAG. Histological analysis showed that ChR2‐labeled Cbln2+ ICx axon terminals were distributed within the dPAG, where they apposed H2B‐EGFP‐labeled glutamatergic neurons (Figure 6j). In acute slices containing the dPAG, light stimulation evoked postsynaptic currents in dPAG neurons that were abolished by APV/CNQX but not by picrotoxin, indicating that the Cbln2+ ICx–dPAG projection is glutamatergic. Notably, light‐evoked PSCs were markedly larger in H2B‐EGFP‐labeled dPAG neurons than in unlabeled neurons, supporting the existence of functional synaptic connections between Cbln2+ ICx neurons and dPAG glutamatergic neurons (Figure 6k, l). Consistent with its behavioral effect, activation of this pathway induced robust pupil dilation together with marked increases in heart rate and breathing rate (Figure 6e–i and Figure S10d–f). In addition, photostimulation of the Cbln2+ ICx‐dPAG terminals in the dPAG induced conditioned place aversion and promoted fear memory formation in the fear conditioning assay (Figure S10g, h).

To monitor the activity of dPAG‐projecting Cbln2+ ICx neurons during auditory defense, we next used an intersectional fiber photometry strategy by injecting AAV2‐retro‐DIO‐GCaMP6s into the dPAG of Cbln2‐IRES‐Cre mice (Figure S10n, o). Fiber photometry was performed to record GCaMP fluorescence (sampling rate: 100 Hz) in parallel with sound stimulation in these mice. 17–20 kHz sound elicited a robust calcium response in dPAG‐projecting Cbln2+ ICx neurons, whereas the three control sounds produced little or no response (Figure S10p–s). Thus, the projection‐defined Cbln2+ ICx–dPAG population selectively encodes the threat‐associated sound stimulus.

Finally, to test whether this pathway is required for sound‐evoked innate fear, we used a dual‐AAV strategy, AAV2‐retro‐DIO‐Flp was injected into the dPAG and AAV‐fDIO‐taCasp3 or AAV‐fDIO‐EGFP was injected into the ICx of Cbln2‐IRES‐Cre mice. Ablation of this projection‐defined population markedly attenuated 17–20 kHz‐evoked defensive responses, including reduced escape speed (Figure 6q–t), diminished conditioned aversion and fear memory (Figure S10j). Furthermore, no significant alterations were observed in autonomic responses (Figure 6u–y and Figure S10k–m). Together, these findings identify the Cbln2+ ICx–dPAG pathway as a major circuit substrate for sound‐evoked defensive behavior.

### 2.7 The Cbln2+ ICx–dPAG Pathway Contributes to Repeated Sound‐Induced Anxiety‐Like Behavior

Interestingly, chronically activating the pathway (1 h per day for 3 days) further induced anxiety‐like behavior, as evidenced by reduced center duration and fewer center entries in the open field test, as well as reduced open‐arm time in the elevated plus maze (Figure 6m–p). We further explored the postsynaptic target of the dPAG glutamatergic neurons that receive ICx input. To this end, AAV1‐Ef1α‐DIO‐Flp was injected into the ICx and AAV‐Ef1α‐fDIO‐mCherry was injected into the dPAG of Vglut2‐IRES‐Cre mice. The mCherry‐labeled axon terminals were mainly distributed in AHN (Figure S10i). These data indicate that optogenetic activation of the Cbln2+ ICx‐dPAG circuit is sufficient to drive robust innate fear responses, and that excessive activation of this circuit further promotes anxiety‐like behavior.

**FIGURE 7:**
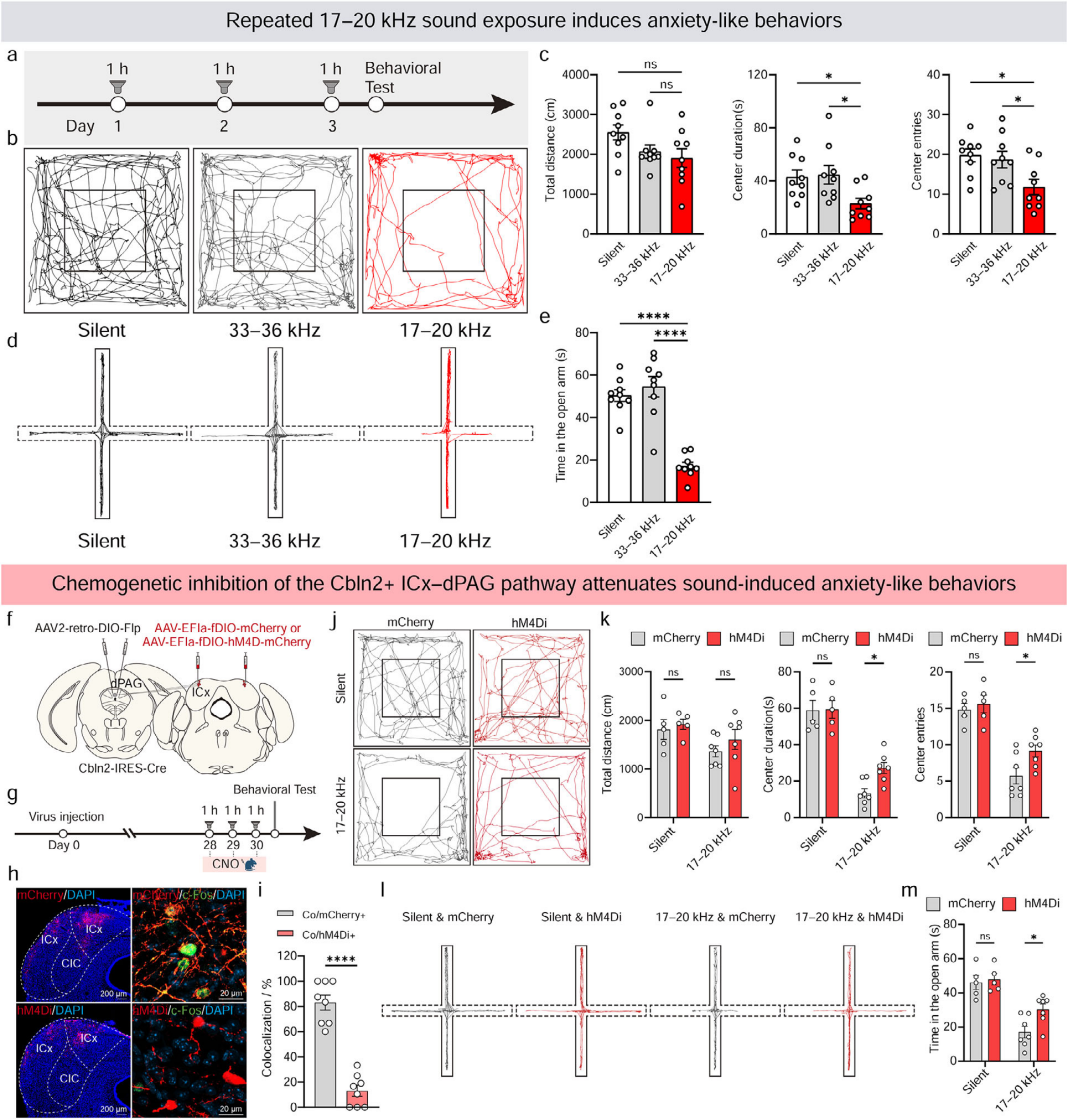
Repeated 17–20 kHz sound exposure induces anxiety‐like behaviors that are attenuated by inhibition of the Cbln2+ ICx–dPAG pathway. (a) Experimental timeline for repeated sound exposure and subsequent behavioral testing. (b) Representative locomotor trajectories in the open field test (OFT) from mice exposed to silent conditions, 33–36 kHz sound, or 17–20 kHz sound. (c) Quantification of total distance traveled, time spent in the center, and number of center entries during the OFT (n = 9 mice per group). (d) Representative locomotor trajectories in the elevated plus maze (EPM) following repeated exposure to silent conditions, 33–36 kHz sound, or 17–20 kHz sound. (e) Quantification of time spent in the open arms of the EPM (n = 9 mice per group). (f) Schematic of the intersectional viral strategy used to selectively express hM4Di in Cbln2+ ICx neurons projecting to the dPAG of Cbln2‐IRES‐Cre mice. (g) Experimental timeline. CNO was administered 30 min before each 1‐h sound‐exposure session for 3 consecutive days. Behavioral testing was performed immediately after the final exposure on Day 3, with no sound presented during testing. (h) Representative images showing c‐Fos expression in pathway‐targeted ICx neurons in the mCherry and hM4Di groups following 17–20 kHz sound stimulation in the presence of CNO. (i) Quantification of the percentage of c‐Fos+ neurons among virus‐labeled Cbln2+ ICx–dPAG neurons, confirming effective chemogenetic suppression by hM4Di (mCherry, n = 8 fields; hM4di, n = 8 fields). (j) Representative locomotor trajectories in the open field test (OFT) from mice in the Silent + mCherry, Silent + hM4Di, 17–20 kHz + mCherry, and 17–20 kHz + hM4Di groups. (k) Quantification of total distance traveled, time spent in the center, and number of center entries during the OFT (Silent + mCherry, n = 5 mice; Silent + hM4Di, n = 5 mice; 17–20 kHz + mCherry, n = 7 mice; 17–20 kHz + hM4Di, n = 7 mice). (l) Representative locomotor trajectories in the elevated plus maze (EPM) from the four experimental groups. (m) Quantification of time spent in the open arms of the EPM. Chemogenetic inhibition of the Cbln2+ ICx–dPAG pathway during repeated 17–20 kHz sound exposure significantly attenuated the reduction in open‐arm exploration (Silent + mCherry, n = 5 mice; Silent + hM4Di, n = 5 mice; 17–20 kHz + mCherry, n = 7 mice; 17–20 kHz + hM4Di, n = 7 mice) All graphs are represented as mean ± SEM, *p < 0.05, **p < 0.01. For detailed statistical information, see Table S4.

To determine whether repeated exposure to the 17–20 kHz auditory stimulus could reproduce the anxiety‐like phenotype induced by chronic activation of the Cbln2+ ICx–dPAG pathway, we exposed mice to 17–20 kHz sound for 1 h per day over 3 consecutive days (Figure 7a). Compared with silent and 33–36 kHz sound controls, repeated 17–20 kHz exposure significantly reduced center exploration in the open field test and open‐arm exploration in the elevated plus maze, without significantly affecting total locomotor activity (Figure 7b–e). These results indicate that repeated exposure to the threat‐associated sound is sufficient to induce anxiety‐like behaviors.

We next asked whether the Cbln2+ ICx–dPAG pathway is required for these sound‐induced anxiety‐like behaviors. Using an intersectional chemogenetic strategy, hM4Di was selectively expressed in Cbln2+ ICx neurons projecting to the dPAG (Figure 7f–i). Chemogenetic inhibition of this pathway during repeated 17–20 kHz sound exposure significantly attenuated the reduction in center exploration in the open field test and open‐arm exploration in the elevated plus maze, while having little effect under silent conditions (Figure 7j–m). Together, these findings demonstrate that repeated threat‐associated sound exposure induces anxiety‐like behaviors and that recruitment of the Cbln2+ ICx–dPAG pathway contributes critically to this behavioral phenotype.

### 2.8 *Cbln2* Regulates Synaptic Transmission Within the CN–ICx–dPAG Circuit

We next mapped monosynaptic inputs to dPAG‐projecting Cbln2+ ICx neurons using rabies‐based retrograde tracing (Figure S11a, b). Labeled upstream inputs were observed in multiple regions, including the cochlear nuclei, superior olive, nuclei of the lateral lemniscus, contralateral IC, parabigeminal nucleus, auditory cortex, ATm, and S1Tr (Figure S11c, d). These results indicate that dPAG‐projecting Cbln2+ ICx neurons are positioned to integrate both ascending auditory inputs and descending or multisensory signals. Given the prominent input from the cochlear nuclei, we next tested the functional contribution of the CN–ICx pathway to sound‐evoked defensive behavior. Selective ablation of glutamatergic CN neurons projecting to the ICx markedly reduced locomotor responses during 17–20 kHz sound stimulation (Figure S11e–h). These findings indicate that the CN–ICx pathway provides an important ascending auditory input required for 17–20 kHz sound‐evoked defensive responses.

We next examined the synaptic mechanism underlying the behavioral effects of *Cbln2* knockdown. *Cbln2* knockdown markedly reduced *Cbln2* expression without neuronal loss in the ICx (Figure S12a–c). Notably, optogenetically evoked EPSCs recorded from dPAG glutamatergic neurons were significantly reduced following *Cbln2* knockdown, indicating weakened Cbln2+ ICx–dPAG synaptic transmission (Figure S12d–f). In addition, *Cbln2* knockdown significantly reduced glutamatergic CN‐evoked EPSCs in Cbln2+ ICx neurons, indicating weakened CN–Cbln2+ ICx synaptic transmission (Figure S12g–i). Together, these findings indicate that *Cbln2* is required for maintaining synaptic efficacy at both the glutamatergic CN–Cbln2+ ICx input and the Cbln2+ ICx–dPAG output, providing a mechanistic basis for the reduced defensive responses following *Cbln2* knockdown.

## 3 Discussion

**FIGURE 8:**
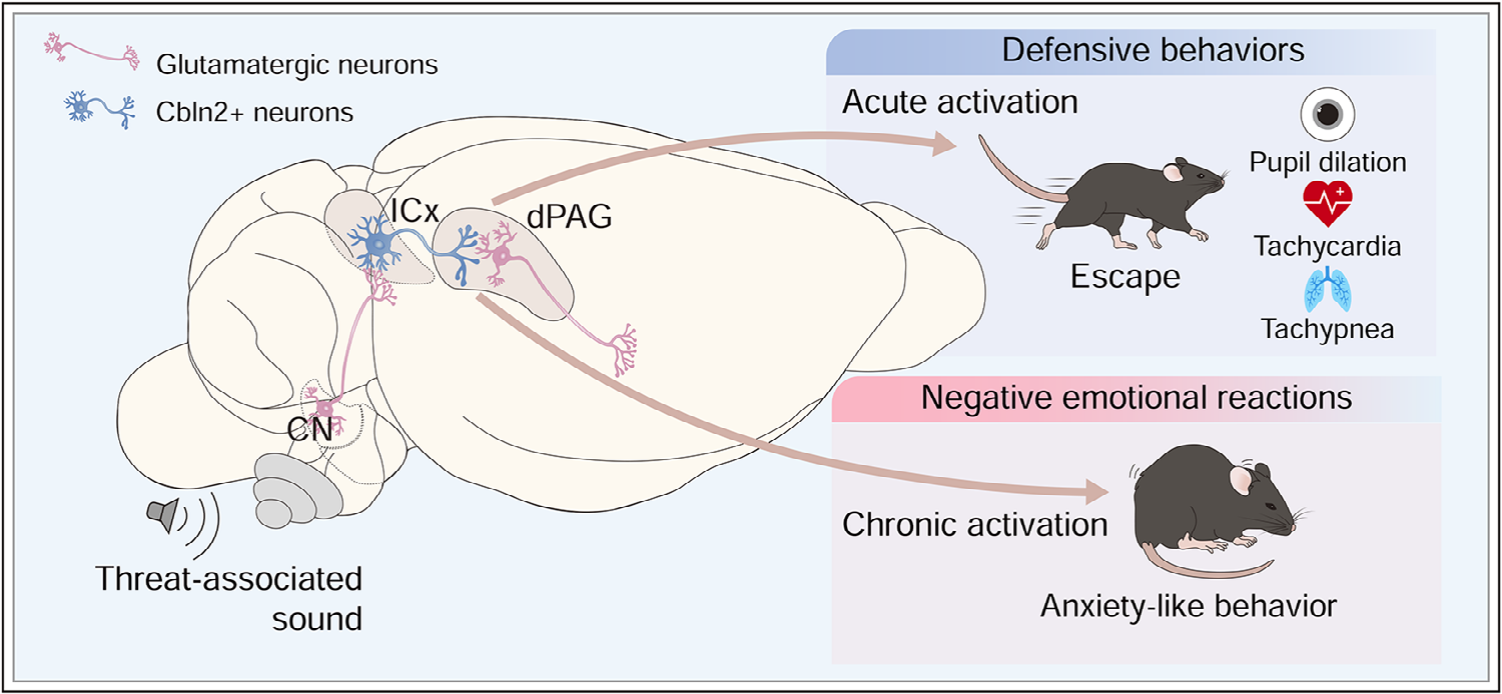
Schematic diagram showing the Cbln2+ ICx–dPAG circuit for sound‐driven defensive and anxiety‐like behaviors.

Threatening sounds can rapidly trigger defensive reactions, yet the cell types and subcortical circuits that transform specific acoustic threat cues into behavioral and autonomic outputs have remained poorly defined. Here, we identify Cbln2+ neurons in the ICx as a molecularly defined excitatory population that specifically detects 17–20 kHz threat‐associated sound and is both sufficient and required for sound‐evoked defensive responses. We further show that this population drives defensive behavior and autonomic arousal through a projection to the dPAG, and that repeated activation of this pathway promotes anxiety‐like behavior. Importantly, repeated 17–20 kHz sound exposure also induced anxiety‐like behavior, which was attenuated by inhibition of the Cbln2+ ICx–dPAG pathway. We further identify glutamatergic CN input as an upstream component and show that *Cbln2* supports synaptic transmission along the CN–ICx–dPAG pathway. Together, these findings define a circuit linking threat‐related auditory processing to defensive and anxiety‐like behaviors (Figure 8).

### 3.1 Experimental Paradigm for Assessing Sound‐Evoked Escape Behavior

In many social species, alarm signals serve to warn conspecifics of potential danger and thereby promote collective survival [27, 28, 29]. In rodents, threat‐related states are often accompanied by ultrasonic vocalizations around 20 kHz [30]. Intriguingly, such innately aversive sweeps have been shown to elicit robust escape behavior in mice [17, 26, 31]. In the present study, we used a single 17–20 kHz acoustic stimulus (2 s, 80 dB) to probe sound‐evoked escape behavior. Whereas prior work has largely focused on escape speed or real‐time aversion induced by loud or broadband noise [12, 13, 14], comparatively less attention has been paid to autonomic responses and auditory fear memory, both of which have been widely incorporated into studies of visual‐ and olfactory‐evoked innate fear [2, 32, 33]. Our behavioral paradigm extends previous studies of sound‐evoked innate defense by capturing multiple dimensions of the defensive state, including pupil dilation, changes in heart rate and breathing rate, conditioned place aversion, and fear memory formation, thereby enabling a more comprehensive assessment of auditory‐evoked innate defensive responses (Figures 1 and S1).

### 3.2 Importance of ICx in Auditory‐Driven Defensive Behavior

An important question raised by our study is how cortical and subcortical pathways cooperate in auditory defense. Previous work showed that the ACx can amplify and directly drive sound‐evoked flight through corticofugal projections to the ICx, which then recruit the dPAG [12]. In our paradigm, ablation of ACx glutamatergic neurons did not significantly impair 17–20 kHz‐evoked escape speed, although it did reduce conditioned freezing to the tone. Together, these results suggest that rapid escape to 17–20 kHz sound is mediated mainly by an ICx‐centered subcortical circuit in our conditions, whereas ACx glutamatergic neurons contribute more to associative components of auditory threat processing. (Figures 2 and S2, 3).

### 3.3 A Critical Role of Cbln2+ ICx Neurons in Auditory‐Driven Escape Behaviors

Previous studies have reported that glutamatergic ICx neurons are involved in mediating tactile vibration sensing [34]. Consistently, the pivotal role of these neurons in 17–20 kHz‐induced escape behaviors is further substantiated by our findings, suggesting substantial functional heterogeneity within the glutamatergic IC population. Given the challenges in classifying IC neurons using traditional morphological and physiological criteria [16], snRNA‐seq was employed, revealing two distinct clusters of excitatory neurons. In this study, *Cbln2* and *Cck* were selected as representative marker genes to differentiate these excitatory populations (Figure 3).

We compared the respective roles of these two excitatory subtypes in 17–20 kHz‐induced escape behaviors by combining optogenetic activation, fiber photometry, and viral‐mediated selective inactivation. Consequently, Cbln2+ ICx neurons were identified as the critical subtype mediating these escape responses. Furthermore, single‐unit optrode recordings revealed that the firing rates of these neurons are positively correlated with the intensity of the acoustic stimuli (Figures 4, 5, and S4, S5, S7,S8). Notably, we found that the knockdown of *Cbln2* within the ICx significantly attenuates both 17–20 kHz–induced escape behaviors and fear memory formation (Figure S6). Because *Cbln2* is a secreted synaptic organizer that links presynaptic neurexins to postsynaptic GluD receptors and regulates synapse assembly, maintenance, and glutamatergic signaling [35, 36], we further examined whether the attenuated defensive responses following *Cbln2* knockdown were associated with impaired synaptic transmission. *Cbln2* knockdown reduced *Cbln2* expression without detectable neuronal loss and significantly weakened both glutamatergic CN–Cbln2+ ICx input and Cbln2+ ICx–dPAG output transmission (Figure S12). These findings provide direct functional evidence that *Cbln2* contributes to maintaining synaptic efficacy along the CN–ICx–dPAG pathway and suggest that impaired transmission through this circuit may underlie the attenuated defensive responses following *Cbln2* knockdown. Future studies should determine how *Cbln2* regulates synaptic organization at the molecular level.

Collectively, we identify Cbln2+ neurons as the excitatory ICx subtype most tightly linked to 17–20 kHz‐evoked innate fear. In contrast, Cck+ ICx neurons displayed a distinct response profile, with stronger activity to higher‐frequency sounds, raising the possibility that separate molecularly defined ICx subpopulations are specialized for threat‐related versus socially relevant acoustic signals.

### 3.4 Role of the Cbln2+ ICx–dPAG Pathway in Auditory‐Driven Escape Behavior

In the present study, our findings demonstrated that the Cbln2+ ICx–dPAG pathway is essential for 17–20 kHz‐evoked escape behaviors. Among several projection‐defined targets tested, only terminal stimulation in the dPAG robustly evoked escape behavior, autonomic arousal, conditioned place aversion, and fear‐conditioning phenotypes. Slice physiology further showed that Cbln2+ ICx terminals form functional glutamatergic synapses onto dPAG glutamatergic neurons. These findings are consistent with prior evidence that dPAG circuits are specialized for escape initiation and defensive arousal [37, 38, 39]. At the same time, our results extend this literature by identifying the upstream, molecularly defined auditory input that recruits this midbrain escape node in response to threat‐associated sound (Figures 6 and S9, S10).

Our rabies‐based tracing further showed that dPAG‐projecting Cbln2+ ICx neurons receive substantial monosynaptic input from brainstem auditory relay nuclei, particularly the CN (Figure S11). Together with previous evidence for direct CN–ICx projections [40, 41], selective ablation of glutamatergic CN neurons projecting to the ICx markedly attenuated 17–20 kHz‐evoked defensive responses. These findings identify the CN–ICx pathway as a functionally important ascending component of the CN–ICx–dPAG circuit for threat‐associated auditory defense.

Given accumulating clinical evidence linking dysregulated fear states, particularly phobias and panic disorder, to anxiety pathogenesis [42, 43, 44], we asked whether sustained recruitment of this circuit could promote a more persistent affective state. Indeed, chronic activation of the Cbln2+ ICx–dPAG pathway was sufficient to induce anxiety‐like states. Repeated optogenetic activation of this pathway was sufficient to induce anxiety‐like behavior, and repeated 17–20 kHz sound exposure produced a similar phenotype that was attenuated by chemogenetic inhibition of the Cbln2+ ICx–dPAG pathway (Figures 6 and 7). These findings suggest that sustained recruitment of this circuit contributes to the transition from acute defensive responses to anxiety‐like states. Circuit tracing further identified the AHN as a candidate downstream target of dPAG glutamatergic neurons receiving ICx input. This is consistent with recent studies showing that the AHN links anxiety‐like avoidance with innate defensive responses in a cell‐type‐specific manner [45, 46]. A limitation of the present study is that only male mice were used. Therefore, whether Cbln2+ ICx neurons and the ICx–dPAG pathway contribute similarly to sound‐evoked defensive behavior and anxiety‐like states in females remains unknown. Future studies should include both sexes and systematically examine sex as a biological variable.

In summary, we identify Cbln2+ ICx neurons as a molecularly defined auditory threat‐detecting population and establish the Cbln2+ ICx–dPAG projection as a key circuit substrate for sound‐evoked innate defense. These findings further suggest that threat‐selective auditory processing can emerge within a subcortical node and be routed directly to midbrain defense circuitry. By identifying a genetically defined ICx cell type and its projection‐defined defensive circuit, this study provides insight into how threat‐relevant auditory signals are transformed into rapid defensive responses and anxiety‐like states.

## Author Contributions

C.S., J.C. and X.L. conceived the study. J.C. and Q.Z. did injections and fiber implantation. J.C., P.W. and Z.Y. did in vivo electrophysiology. L.H. did slice physiology. X.L. and J.C. did behavioral tests and in vivo 2P Ca2+ imaging. X.L. and Z.Z. performed the in situ hybridization assay and immunohistochemistry. C.S., J.C. and C.M. wrote the manuscript with help from all authors. Every author reviewed the manuscript.

## Conflicts of Interest

The authors declare no conflicts of interest.

## Supporting information




**Supporting File 1**: advs77983‐sup‐0001‐SuppMat.docx.

**Supporting File 2**: advs77983‐sup‐0002‐Tables.docx.

**Supporting File 3**: advs77983‐sup‐0003‐Figures.pdf.

**Supporting File 4**: advs77983‐sup‐0004‐SuppMat.docx.

**Supporting File 5**: advs77983‐sup‐0005‐MovieS1.mp4.

**Supporting File 6**: advs77983‐sup‐0006‐MovieS2.mp4.

**Supporting File 7**: advs77983‐sup‐0006‐MovieS3.mp4

**Supporting File 8**: advs77983‐sup‐0006‐MovieS4.mp4.

**Supporting File 9**: advs77983‐sup‐0009‐MovieS5.mp4.

## Data Availability

The data that support the findings of this study are available from the corresponding author upon reasonable request.
