# Supplementary material for: A Molecularly Defined Midbrain Circuit Mechanism for Sound‐Driven Defensive Behavior and Anxiety
Source: Adv Sci (Weinh). 2026 Sep 27:e77983. Online ahead of print. doi: 10.1002/advs.77983 (PMC13616374; doi:10.1002/advs.77983)
Supplement: Supplementary file 1 [file ADVS-9999-e77983-s008.pdf]

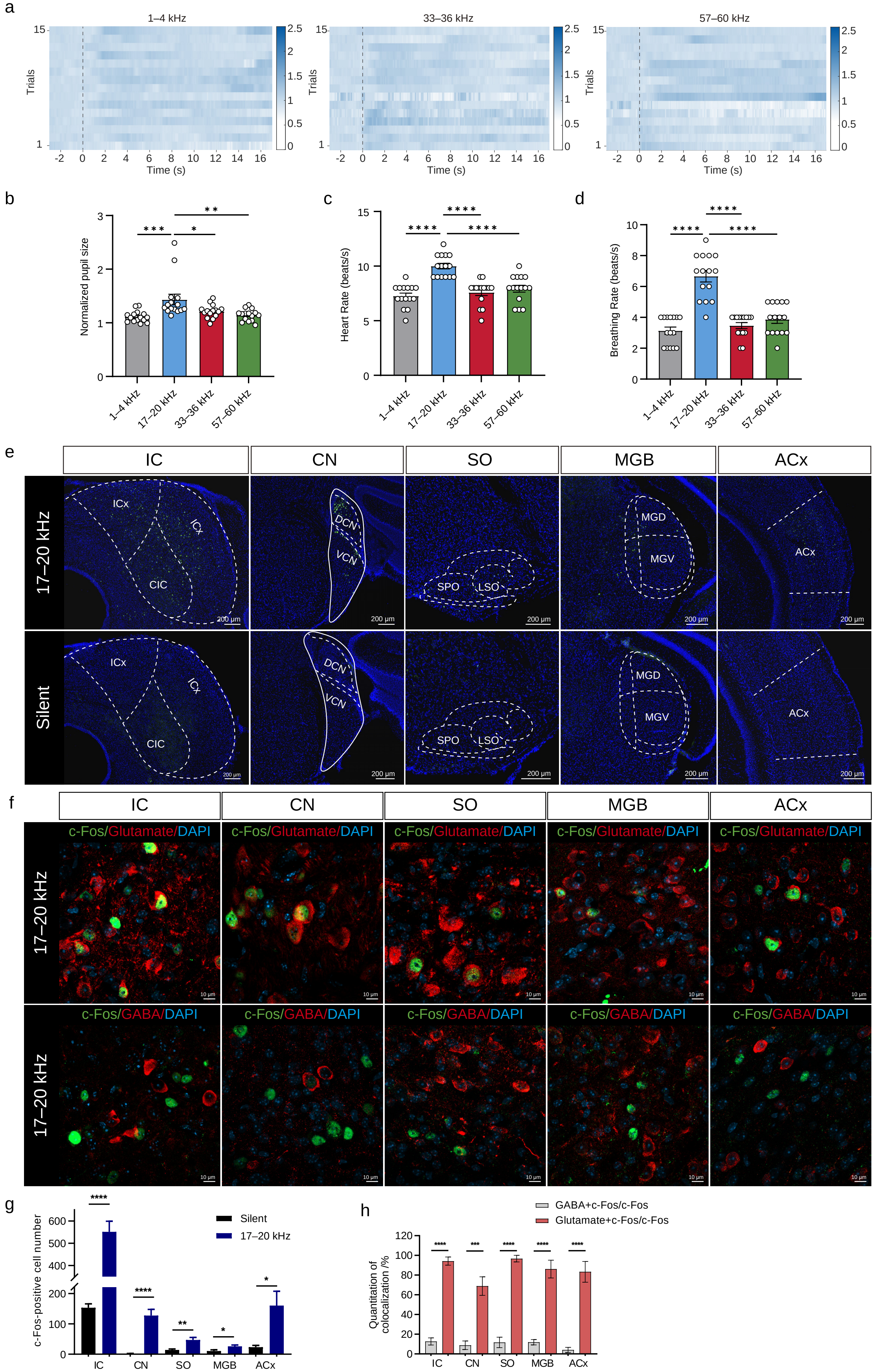

Figure S1 See next page for figure legends.

Figure S1. 17–20 kHz sound preferentially activates glutamatergic neurons in the auditory pathway.
(a) Heatmaps of pupil dilation bouts in response to frequency-modulated sweep of 1–4 kHz (left), 33–36 kHz (middle), and 57–60 kHz (right).
(b) Quantification of maximal normalized pupil size in response to the indicated FM sweep stimuli (n = 5 mice, 15 trials).
(c) Quantification of maximal heart rate in response to the indicated FM sweep stimuli (n = 5 mice, 15 trials).
(d) Quantification of maximal breathing rate in response to the indicated FM sweep stimuli (n = 5 mice, 15 trials).
(e) Representative coronal sections showing Fos expression in auditory regions following 17–20 kHz sound stimulation or under silent control conditions. IC, inferior colliculus; CN, cochlear nucleus; SO, superior olive; MGB, medial geniculate body; ACx, auditory cortex. Scale bars, 200 μm.
(f) Representative images demonstrate Fos-positive neurons colocalized with a glutamate-specific antibody, but not with a GABA-specific antibody. Scale bar: 10 μm.
(g) Quantification of Fos-positive cell numbers in the indicated auditory regions in silent control and 17–20 kHz-stimulated mice (n = 8 fields from 4 mice for silent, n = 10 fields from 5 mice for 17–20 kHz-stimulated group).
(h) Quantification of neurons co-labeled for glutamate/GABA and c-Fos in the indicated auditory regions (n = 6 mice per group).
All graphs are represented as mean ± SEM, *$p < 0.05$, **$p < 0.01$, ***$p < 0.001$ or ****$p < 0.0001$. For detailed statistics information, see Supplementary Table 4.

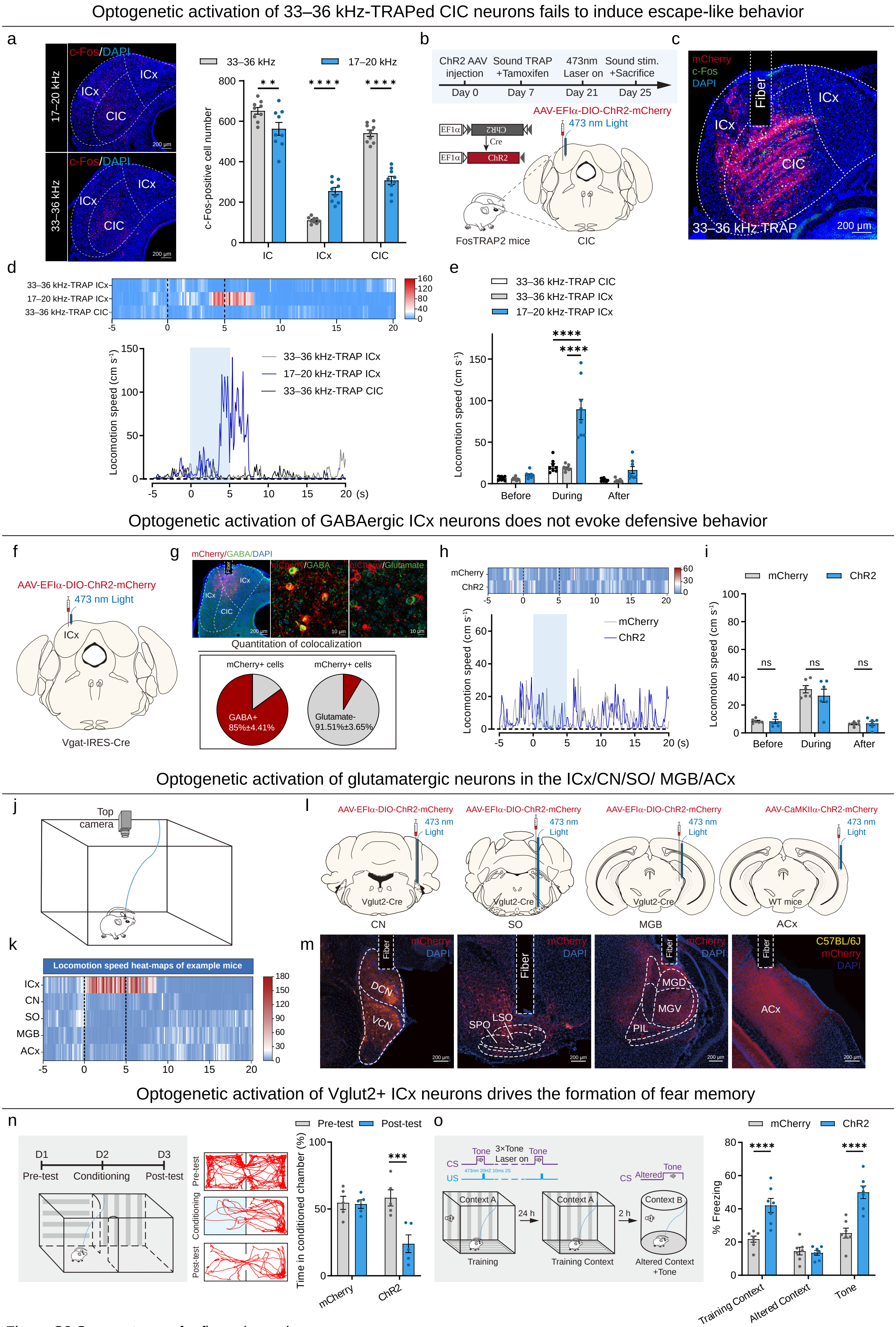

Figure S2 See next page for figure legends.

Figure S2. Functional characterization of distinct neuronal populations in auditory regions during sound-evoked defense.
(a) Representative images of c-Fos+ neurons in the IC following 17–20 kHz or 33–36 kHz sound stimulation (left), together with quantification of Fos+ neurons in the IC, ICx, and central nucleus of the inferior colliculus (CIC) after different sound stimuli (right, n = 9 mice per group).
(b) Schematic of the FosTRAP procedure and injection of AAV-EF1α-DIO-ChR2-mCherry into the CIC followed by optical fiber implantation above the CIC of FosTRAP2 mice.
(c) Representative image showing ChR2-mCherry expression in 33–36 kHz TRAPed CIC neurons after viral labeling and optical fiber implantation.
(d) Example heatmaps of speed time course of photostimulation of 17–20 kHz-TRAPed and 33–36 kHz-TRAPed ICx or CIC neurons (top) and typical time course of locomotion speed (bottom).
(e) Quantitative analyses of locomotion speed before, during, and after optogenetic activation (n = 8 mice per group).
(f) Diagram showing AAV-EF1α-DIO-ChR2-mCherry injection into the ICx followed by optical fiber implantation above the ICx of Vgat-IRES-Cre mice.
(g) Representative coronal section, scale bar, 200 µm. Magnification images showing ChR2 expression in GABAergic but not glutamatergic ICx neurons (top), and quantification of colocalization (bottom; *n* = 6 fields from 3 mice), scale bar, 10 µm.
(h) Example heatmaps of speed time course of optogenetic activation of ICx Vgat+ neurons (top) and typical time course of locomotion speed (bottom).
(i) Quantitative analyses of locomotion speed before, during, and after optogenetic activation (*n* = 6 mice per group).
(j) Schematic diagram of open field test for optogenetic activation-induced behavior. Locomotion was recorded by top positioned cameras.
(k) Example heatmaps of locomotion speed before, during, and after activation of glutamatergic neurons in the indicated auditory regions, with ICx shown as a positive control.
(l) Schematics of optogenetic activation of glutamatergic neurons in the CN, SO, MGB, and ACx. AAV-EF1α-DIO-ChR2-mCherry was injected into the CN, SO, or MGB of Vglut2-Cre mice, and AAV-CaMKIIα-ChR2-mCherry was injected into the ACx of WT mice.
(m) Representative coronal sections showing optical fiber track above ChR2-expressing neurons in CN, SO, MGB, ACx.
(n) Schematic diagram showing the procedure to induce CPA with ICx glutamatergic neurons light stimulation (left). Representative traces (middle). Summarized data from CPA tests (right; *n* = 5 mice per group).
(o) Fear-conditioning paradigm used to test whether optogenetic activation of ICx glutamatergic neurons promotes fear memory formation, with quantification of freezing in the training context, altered context, and tone test (*n* = 7 mice per group).
All graphs are represented as mean ± SEM, *p < 0.05, **p < 0.01, ***p < 0.001 or ****p < 0.0001. For detailed statistics information, see Supplementary Table 4.

Viral-mediated ablation of Vglut2+ ICx neurons impairs sound-driven defensive responses

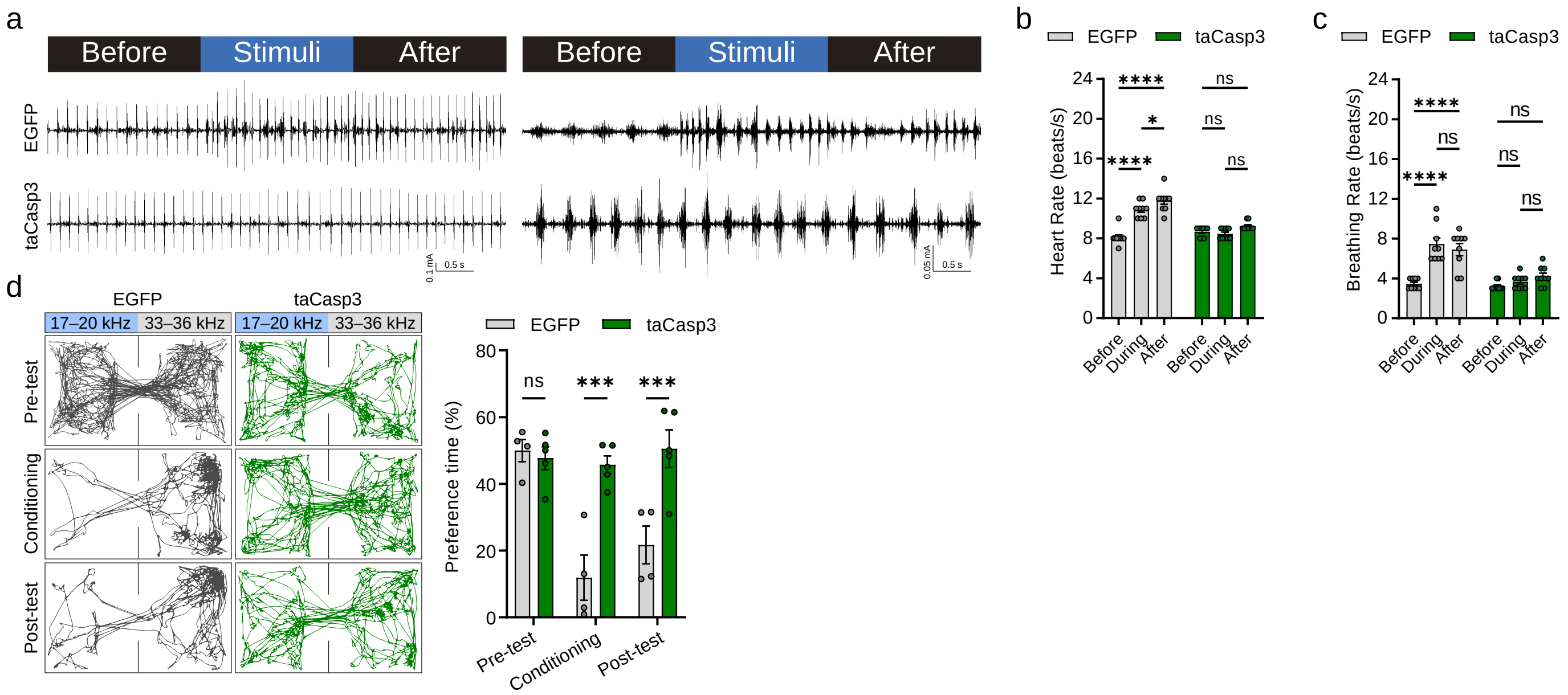

Role of ACx glutamatergic neurons in sound-induced innate defensive behavior

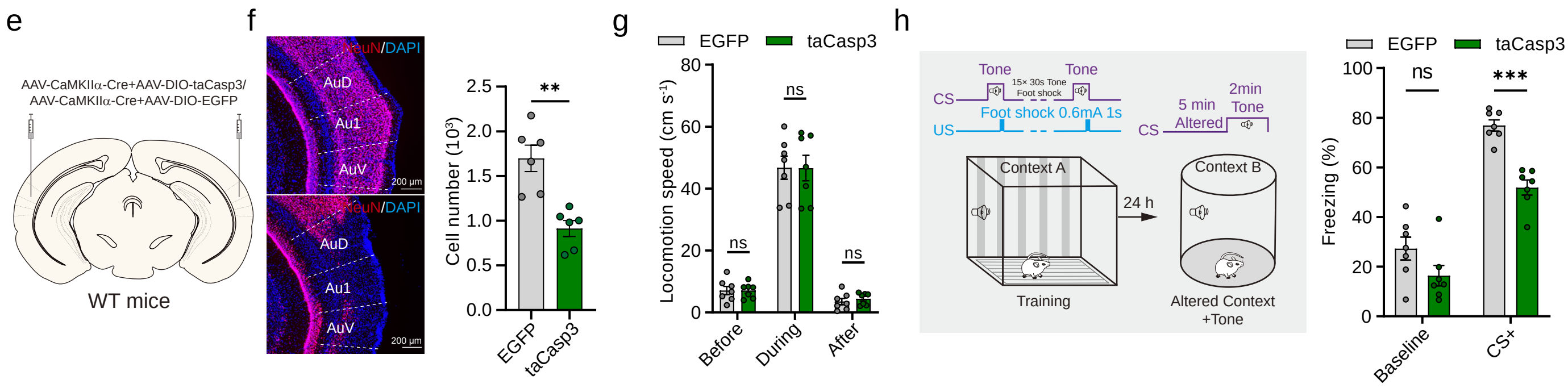

Activity of glutamatergic ICx neurons during sound stimulation

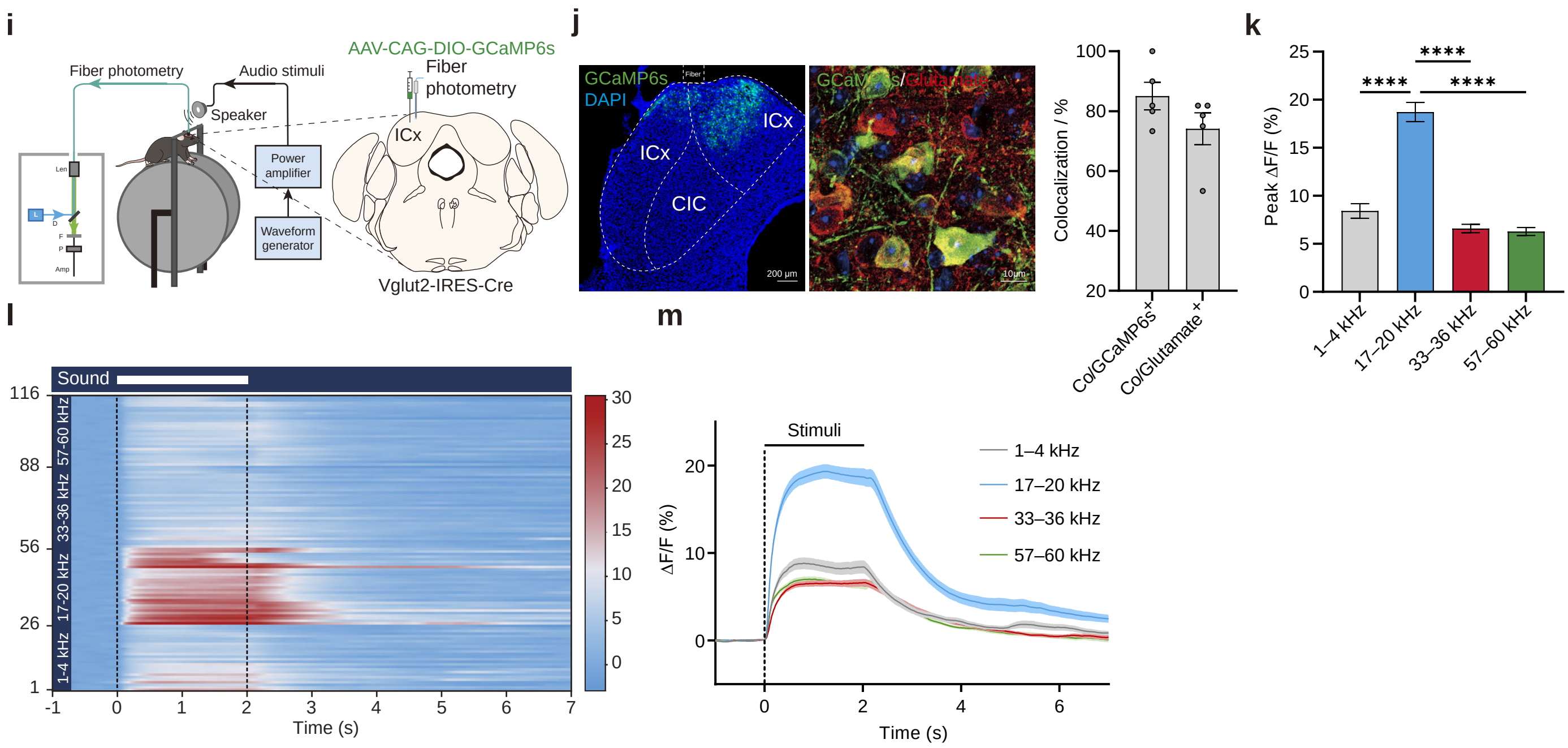

Figure S3 See next page for figure legends.

Figure S3. Differential roles of ICx and ACx glutamatergic neurons in sound-evoked innate defensive responses.
(a) Representative ECG traces (left) and diaphragm EMG traces (right).
(b) Quantification of maximal heart rate before, during (Stimuli), and after 17–20 kHz sound stimulation (*n* = 3 mice, 9 trials per group).
(c) Quantification of maximal breathing rate before, during (Stimuli), and after 17–20 kHz sound stimulation (*n* = 3 mice, 9 trials per group).
(d) Representative traces in the CPA test for control and ICx Vglut2+ neuron-ablated group in response to 17–20 kHz sound stimulation (left). Summarized data from CPA tests (right; *n* = 4 mice per group).
(e) Schematic of virus-induced apoptosis in ACx glutamatergic neurons.
(f) Typical images of taCasp3-induced apoptosis in ACx glutamatergic neurons (left), and summarized data for number of neurons (right; *n* = 6 mice per group). Scale bar, 200 μm.
(g) Quantification of locomotor speed before, during, and after 17–20 kHz sound stimulation in control and ACx glutamatergic neuron-ablated mice (*n* = 7 mice per group).
(h) Schematic diagram showing differential fear-conditioning protocol. Foot shocks were paired with conditioned sound to examine if mice formed fear memory after inactivation of ACx glutamatergic neurons (left). Quantification of freezing under baseline and CS+ conditions in control and ACx glutamatergic neuron-ablated mice (right; *n* = 7 mice per group).
(i) Schematic of fiber photometry recording, AAV-CAG-DIO-GCaMP6s-EGFP was injected into the ICx followed by optical fiber implantation above the ICx of Vglut2-IRES-Cre mice.
(j) Example coronal section showing optical fiber track above GCaMP6s-expressing neurons in ICx of Vglut2-IRES-Cre mice (left). Scale bar, 200 μm. Micrographs showing specific expression of GCaMP6s in Vglut2+ neurons in the ICx (middle). Quantification of the specificity and efficiency of GCaMP6s expression (right; *n* = 5 mice). Scale bar, 10 μm.
(k) Quantification of peak $\Delta F/F$ responses to the indicated sound stimuli (*n* = 5 mice).
(l) Heatmaps of Ca2+ activities of Vglut2+ ICx neurons.
(m) Average GCaMP response curve for Vglut2+ ICx neurons in response to various sound stimuli (*n* = 5 mice).
All graphs are represented as mean ± SEM, *p < 0.05, **p < 0.01, ***p < 0.001 or ****p < 0.0001. For detailed statistical information, see Supplementary Table 4.

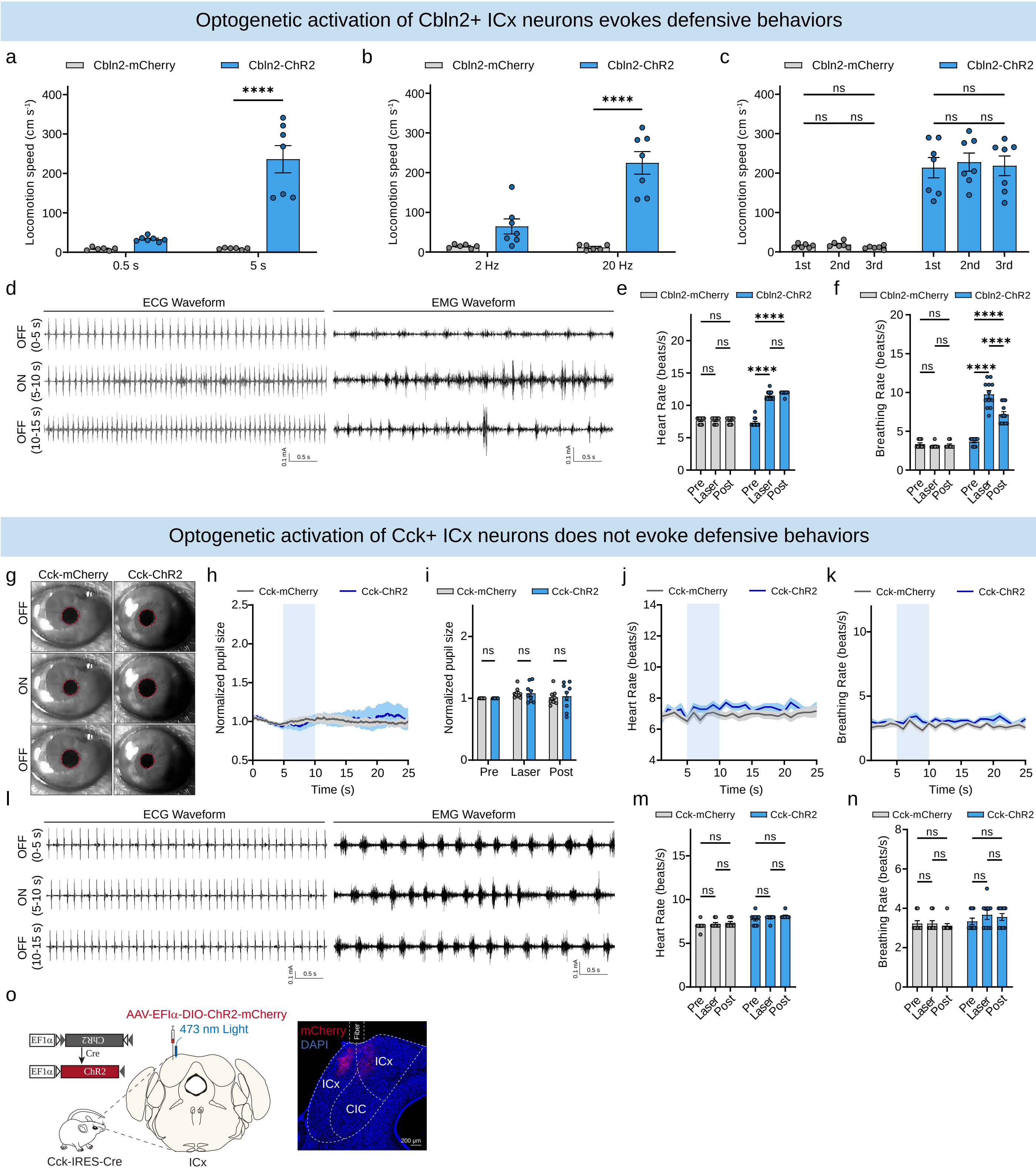

Figure S4 See next page for figure legends.

Figure S4. Optogenetic activation of Cbln2+ but not Cck+ ICx neurons drives defensive behaviors

(a) Quantification of locomotor speed during photostimulation of Cbln2+ ICx neurons with different stimulation durations (*n* = 6 mice for Cbln2-mCherry and *n* = 7 mice for Cbln2-ChR2).

(b) Quantification of locomotor speed during photostimulation of Cbln2+ ICx neurons at different stimulation frequencies (*n* = 6 mice for Cbln2-mCherry and *n* = 7 mice for Cbln2-ChR2).

(c) Quantification of locomotor speed during repeated photostimulation of Cbln2+ ICx neurons across consecutive stimulation bouts (*n* = 6 mice for Cbln2-mCherry and *n* = 7 mice for Cbln2-ChR2).

(d) Representative ECG traces (left) and diaphragm EMG traces (right).

(e) Quantification of maximal heart rate before (Pre), during (Laser), and after (Post) stimulation (*n* = 3 mice, 9 trials for Cbln2-mCherry and *n* = 4 mice, 12 trials for Cbln2-ChR2).

(f) Quantification of maximal breathing rate before (Pre), during (Laser), and after (Post) stimulation (*n* = 3 mice, 9 trials for Cbln2-mCherry and *n* = 4 mice, 12 trials for Cbln2-ChR2).

(g) Typical images of pupil of activation of Cck+ ICx neurons.

(h) Average curves of normalized pupil size.

(i) Summarized data of normalized pupil size (bottom; *n* = 3 mice, 9 trials per group).

(j) Average curves of heart rate.

(k) Average curves of breathing rate.

(l) Representative ECG traces (left) and diaphragm EMG traces (right).

(m) Quantification of maximal heart rate before (Pre), during (Laser), and after (Post) stimulation (*n* = 3 mice, 9 trials per group).

(n) Quantification of maximal breathing rate before (Pre), during (Laser), and after (Post) stimulation (*n* = 3 mice, 9 trials per group).

(o) Schematic of optogenetic activation of Cck+ ICx neurons (left). Coronal ICx section showing the expression of ChR2-mCherry (red) in Cck+ ICx neurons and optical fiber tracks in ICx (right).

All graphs are represented as mean ± SEM, *p < 0.05, **p < 0.01, ***p < 0.001 or ****p < 0.0001. For detailed statistical information, see Supplementary Table 4.

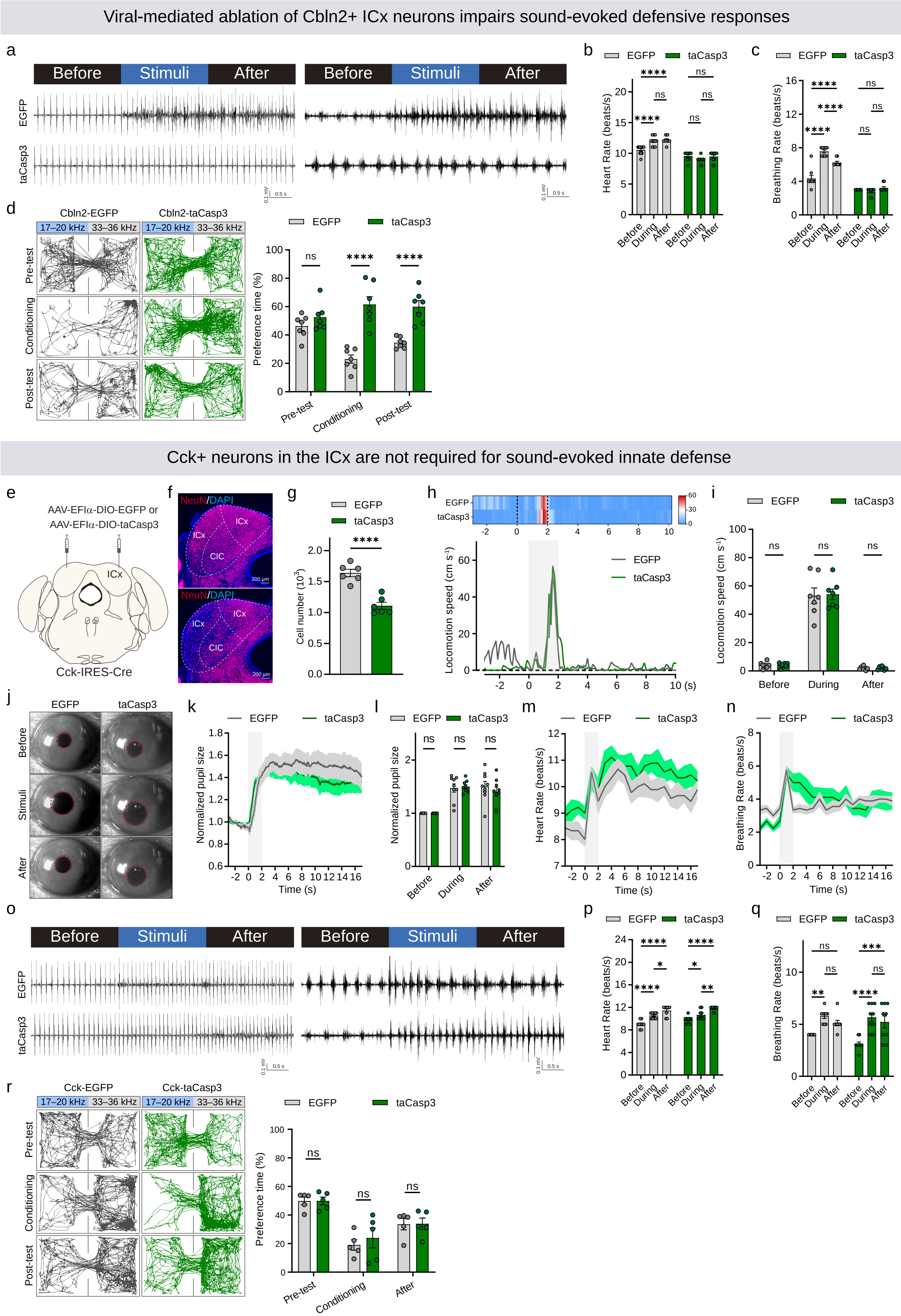

Figure S5 See next page for figure legends.

Figure S5. Cbln2+ but not Cck+ ICx neurons are required for sound-evoked innate defensive responses

(a) Representative ECG traces (left) and diaphragm EMG traces (right).
(b) Quantification of maximal heart rate before, during (Stimuli), and after 17–20 kHz sound stimulation (n = 3 mice, 9 trials per group).
(c) Quantification of maximal breathing rate before, during (Stimuli), and after 17–20 kHz sound stimulation (n = 3 mice, 9 trials per group).
(d) Representative trajectories in the CPA test for control and Cbln2+ ICx neuron-ablated group in response to 17–20 kHz sound stimulation (left). Summarized data from CPA tests (right; n = 7 mice per group).
(e) Schematic of virus-induced apoptosis in Cck+ ICx neurons (left).
(f) Typical images of taCasp3-induced apoptosis in Cck+ ICx neurons. Scale bar, 200 μm.
(g) Summarized data for number of neurons in ICx (n = 6 mice per group).
(h) Heatmaps of speed time course of example mice in response to 17–20 kHz sound stimulation (top) and representative time course of locomotion speed (bottom).
(i) Quantification of locomotor speed before, during, and after 17–20 kHz sound stimulation in control and Cck+ ICx neuron-ablated mice (n = 7 mice per group).
(j) Typical images of pupil of inactivation of Cck+ ICx neurons in response to 17–20 kHz sound stimulation.
(k) Average curves of normalized pupil size.
(l) Summarized data of normalized pupil size (bottom; n = 3 mice, 9 trials per group).
(m) Average curves of heart rate.
(n) Average curves of breathing rate.
(o) Representative ECG traces (left) and diaphragm EMG traces (right).
(p) Quantification of maximal heart rate before, during (Stimuli), and after 17–20 kHz sound stimulation (n = 3 mice, 9 trials per group).
(q) Quantification of maximal breathing rate before, during (Stimuli), and after 17–20 kHz sound stimulation (n = 3 mice, 9 trials per group).
(r) Representative trajectories in the CPA test (left) and quantification of CPA performance in control and Cck+ ICx neuron-ablated mice (right; n = 5 mice per group).
All graphs are represented as mean ± SEM, *p < 0.05, **p < 0.01, ***p < 0.001 or ****p < 0.0001. For detailed statistical information, see Supplementary Table 4.

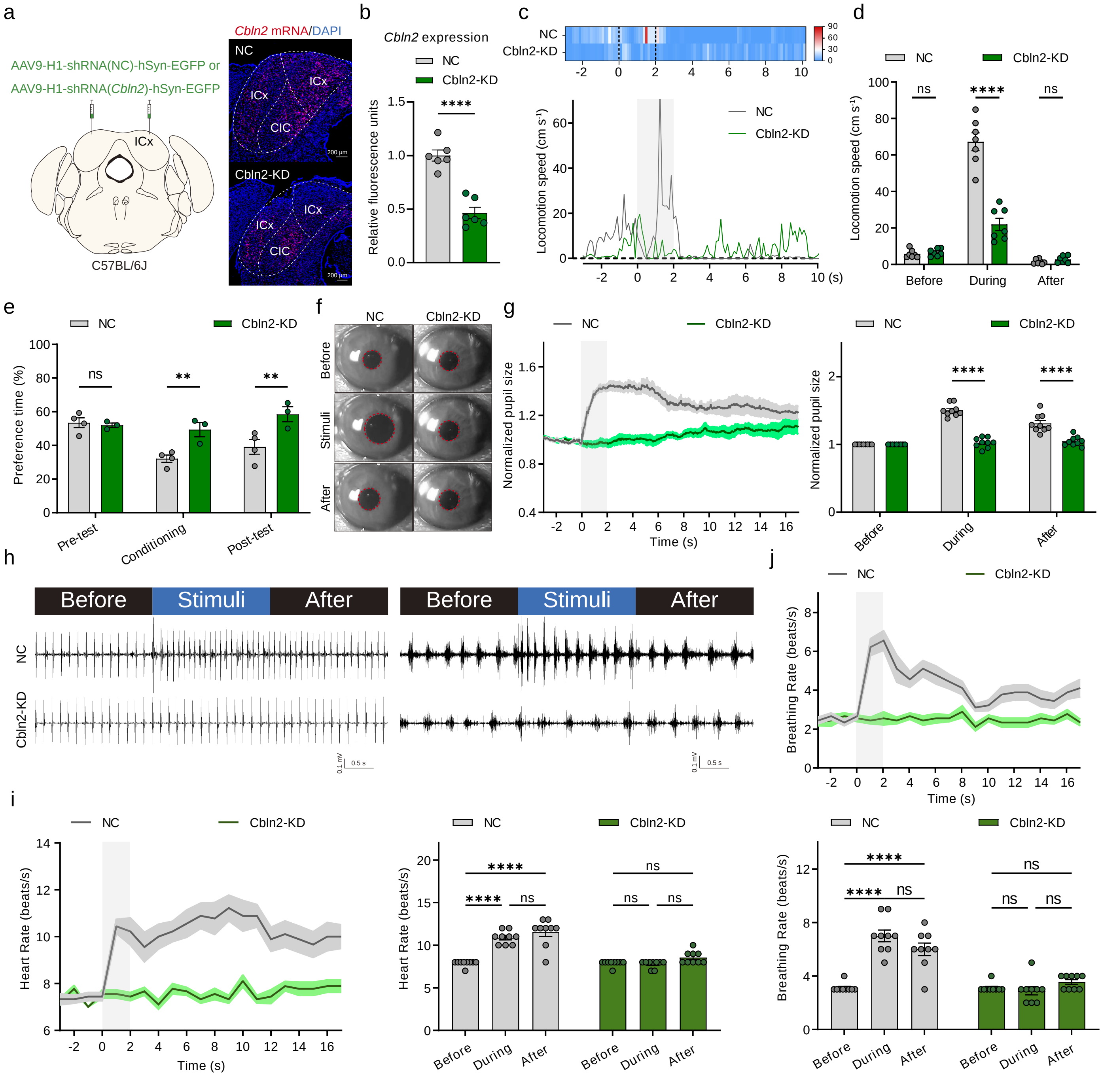

Figure S6. *Cbln2* knockdown in the ICx attenuates sound-evoked defensive and autonomic responses.
(a) Schematic diagram showing the viral injection for *Cbln2* knockdown in mouse ICx (left). Example coronal section showing *Cbln2* mRNA expressing in the ICx of negative control (NC) and Cbln2-knockdown (Cbln2-KD) mice (right; *n* = 6 mice per group). Scale bar, 200 μm.
(b) Quantification of *Cbln2* mRNA fluorescence intensity in the ICx.
(c) Heatmaps of locomotor speed in example mice in response to 17–20 kHz sound stimulation (top) and representative time course of locomotion speed (bottom).
(d) Effects of *Cbln2* knockdown on locomotion speed of mice with sound-induced defensive behavior (*n* = 7 mice per group).
(e) Summarized data from CPA tests (*n* = 4 mice for NC and *n* = 3 mice for Cbln2-KD).
(f) Typical images of pupil from NC and Cbln2-KD mice in response to 17–20 kHz sound stimulation.
(g) Average curves of normalized pupil size (left) and summarized data of normalized pupil size (right; *n* = 3 mice, 9 trials per group).
(h) Example electrocardiographic traces (left) and diaphragm EMG traces (right).
(i) Average curves of heart rate (left) and summarized data of maximal heart rate before, during (Stimuli), and after 17–20 kHz sound stimulation (*n* = 3 mice, 9 trials per group).
(j) Average curves of breathing rate (top) and summarized data of maximal breathing rate before, during (Stimuli), and after 17–20 kHz sound stimulation (*n* = 3 mice, 9 trials per group).
All graphs are represented as mean ± SEM, *p < 0.05, **p < 0.01, ***p < 0.001 or ****p < 0.0001. For detailed statistical information, see Supplementary Table 4.

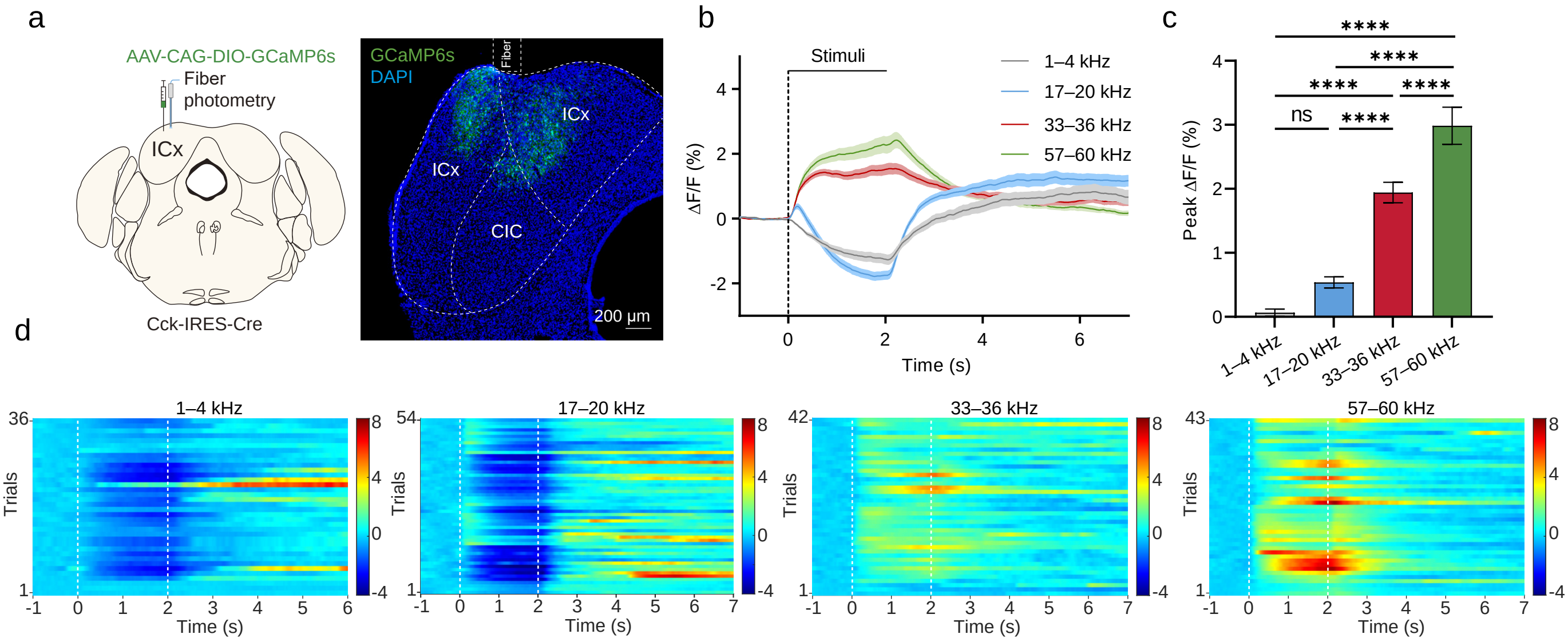

Figure S7. Cck+ ICx neurons preferentially respond to higher-frequency sound stimuli.

(a) Schematic of fiber photometry recording from Cck+ ICx neurons (left) and representative coronal section showing GCaMP6s expression and optical fiber placement in the ICx of Cck-IRES-Cre mice (right). Scale bar, 200 µm.

(b) Average GCaMP response curve.

(c) Quantification of peak ΔF/F responses in Cck+ ICx neurons evoked by the indicated sound stimuli (right; $n = 4$ mice).

(d) Heatmaps of calcium activity of Cck+ ICx neurons for sound stimulation.

All graphs are represented as mean ± SEM, *p < 0.05, **p < 0.01, ***p < 0.001 or ****p < 0.0001. For detailed statistical information, see Supplementary Table 4.

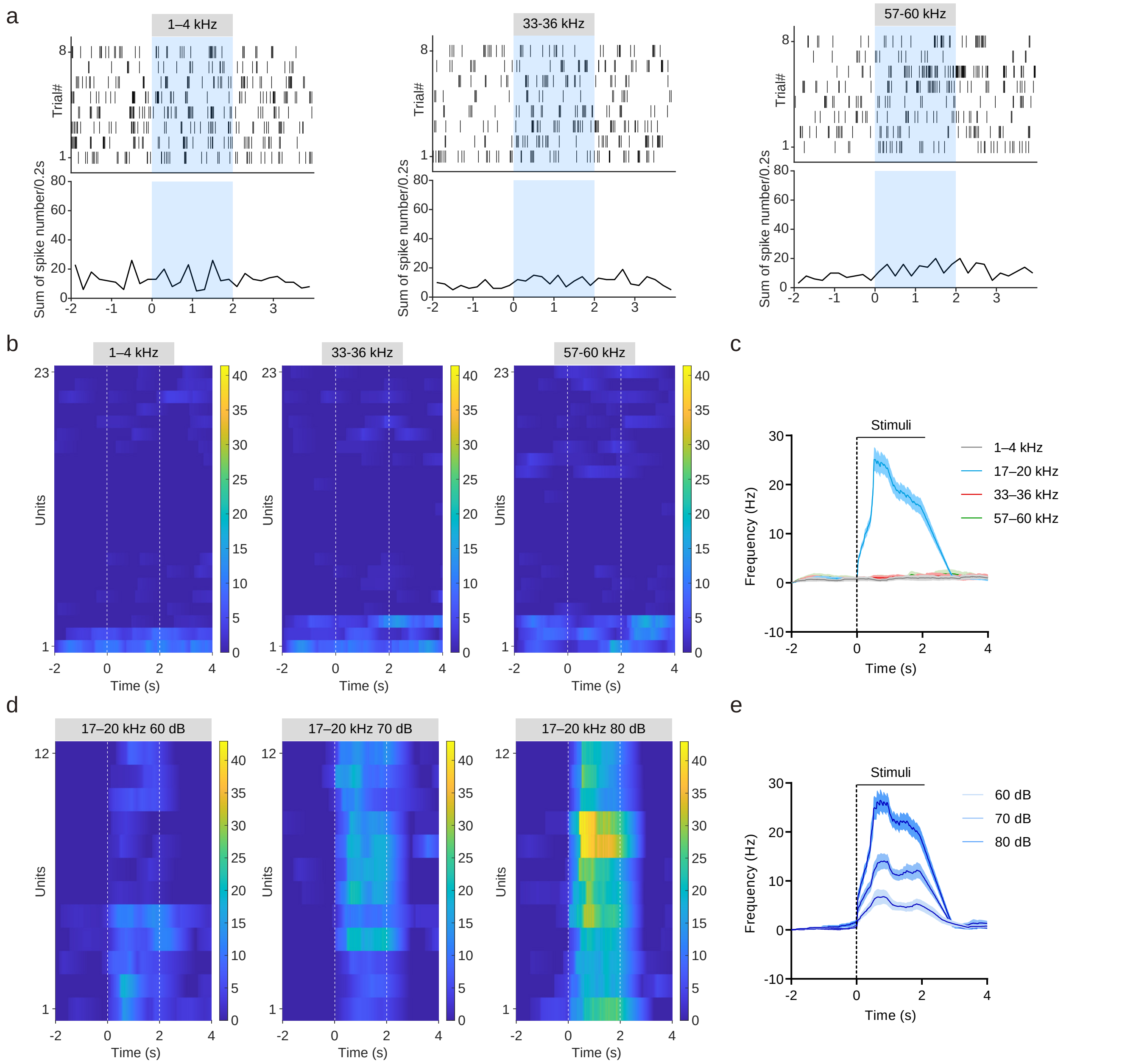

Figure S8. Single-unit response properties of Cbln2+ ICx neurons during sound stimulation.
(a) Raster plots from an example putative Cbln2+ ICx neuron in response to the indicated control sound stimulation (top; *n* = 8 trials), together with the summed spike counts across trials (bottom).
(b) Heat map of firing responses from optotagged Cbln2+ ICx neurons to the indicated control sound stimulation (left; *n* = 23 units).
(c) Mean firing-rate traces of optotagged Cbln2+ ICx neurons in response to the indicated sound stimulation.
(d) Heat map of firing responses from optotagged Cbln2+ ICx neurons to 17–20 kHz sound stimulation at different sound intensities (left; *n* = 12 units).
(e) Mean firing-rate traces of optotagged Cbln2+ ICx neurons in response to 17–20 kHz sound stimulation at different sound intensities. All graphs are represented as mean ± SEM. For detailed statistical information, see Supplementary Table 4.

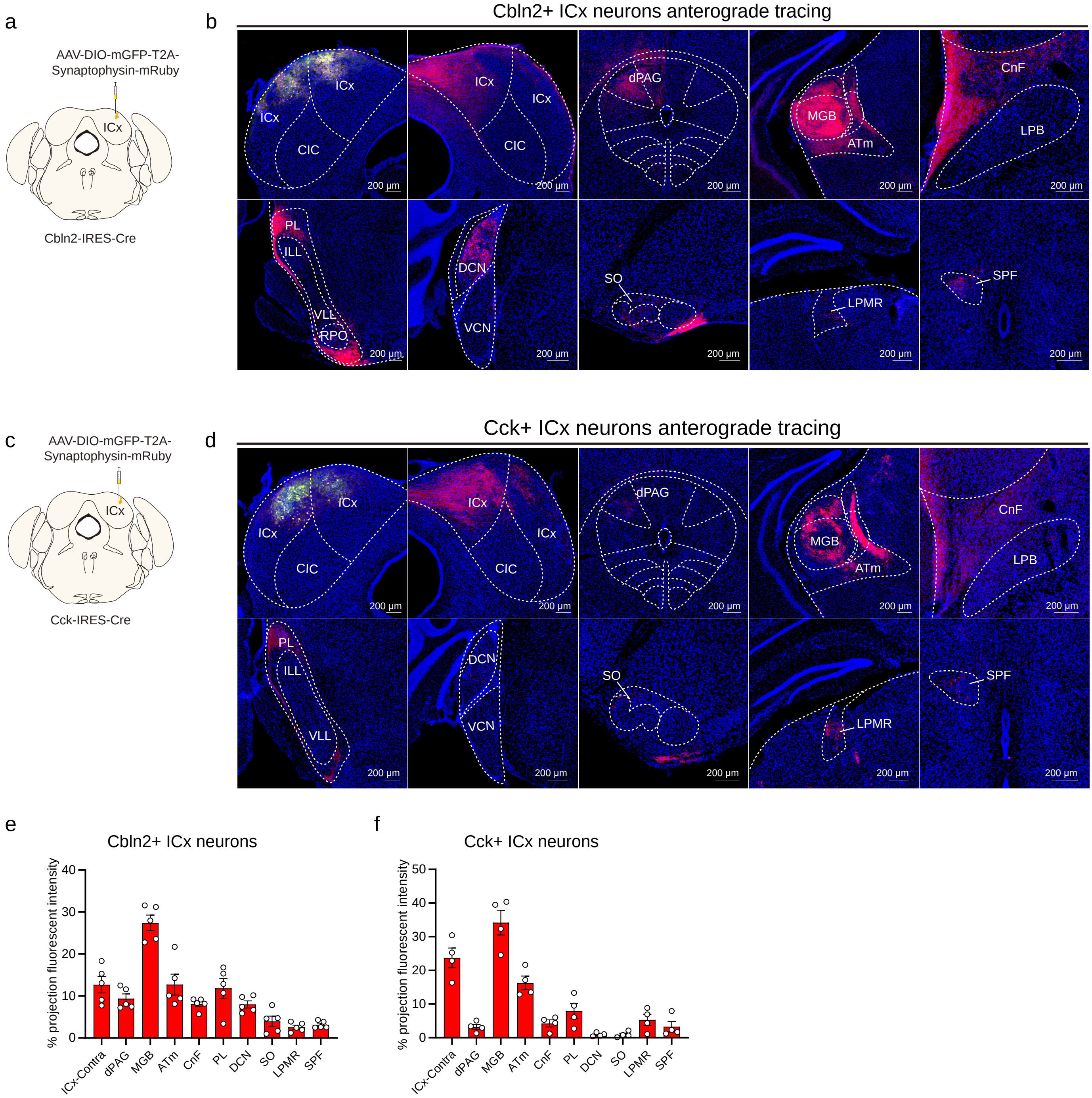

Figure S9. Mapping the output connectivity of Cbln2+ ICx and Cck+ ICx neurons.

(a) Schematic diagram showing injection of AAV-DIO-mGFP-T2A-Synaptophysin-mRuby into the ICx of Cbln2-IRES-Cre mice.

(b) Example coronal brain section showing the viral injection center and mRuby+ axonal projections of Cbln2+ ICx neurons in different brain regions. Scale bars, 200 μm.

(c) Schematic diagram showing injection of AAV-DIO-mGFP-T2A-Synaptophysin-mRuby into the ICx of Cck-IRES-Cre mice.

(d) Example coronal brain section showing the viral injection center and mRuby+ axonal projections of Cck+ ICx neurons in different brain regions. Scale bars, 200 μm.

(e) Fraction of projection fluorescent intensity in different brain regions for Cbln2+ ICx neurons (*n* = 5 mice).

(f) Fraction of projection fluorescent intensity in different brain regions for Cck+ ICx neurons (*n* = 4 mice).

Abbreviations: Central nucleus of the inferior colliculus(CIC), external cortex of the inferior colliculus(ICx), dorsal periaqueductal gray (dPAG), medial part of the auditory thalamus (ATm), cuneiform nucleus (CnF), paralemniscal nucleus (PL), dorsal cochlear nucleus (DCN), superior olive (SO), lateral posterior thalamic nucleus, mediorostral part (LPMR), subparafascicular thalamic nucleus (SPF).

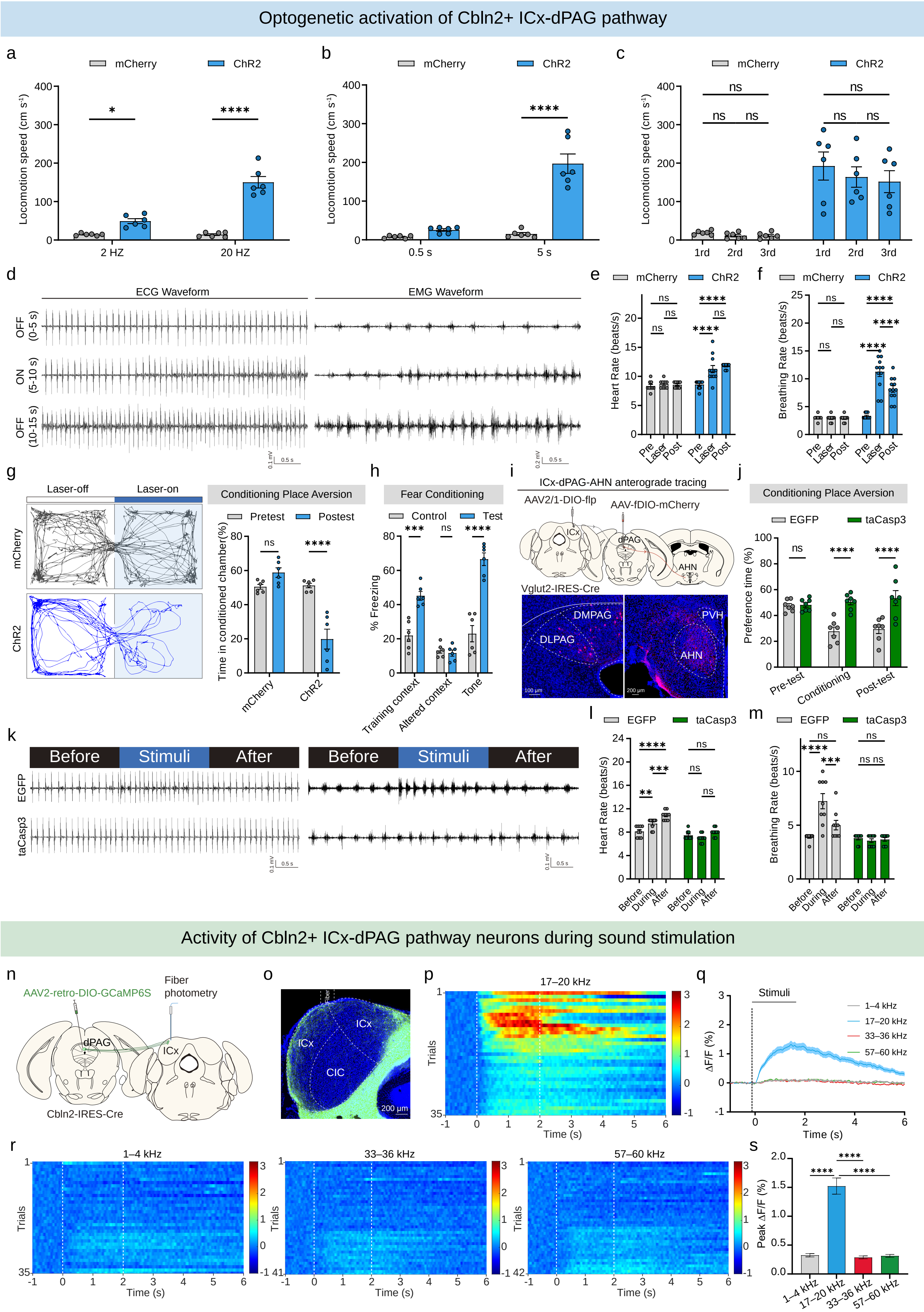

Figure S10. See next page for figure legends.

Figure S10. Activation of the Cbln2+ ICx–dPAG pathway drives defensive responses and encodes threatening auditory stimuli.
(a) Quantification of locomotor speed during optogenetic activation of the ICx-dPAG pathway at different stimulation frequencies (*n* = 6 mice per group).
(b) Quantification of locomotor speed during optogenetic activation of the ICx-dPAG pathway with different stimulation durations (*n* = 6 mice per group).
(c) Quantification of locomotor speed across three consecutive bouts of optogenetic activation of the ICx-dPAG pathway (*n* = 6 mice per group).
(d) Representative ECG traces (left) and diaphragm EMG traces (right) recorded before, during, and after photostimulation.
(e) Quantification of maximal heart rate before (Pre), during (Laser), and after (Post) stimulation (*n* = 3 mice, 9 trials for mCherry and *n* = 4 mice, 12 trials for ChR2).
(f) Quantification of maximal breathing rate before (Pre), during (Laser), and after (Post) stimulation (*n* = 3 mice, 9 trials for mCherry and *n* = 4 mice, 12 trials for ChR2).
(g) Representative trajectories in the conditioned place aversion (CPA) test (left), quantification of time spent in the conditioned chamber during the pre-test and post-test sessions (right; *n* = 6 mice per group).
(h) Quantitative analyses of fear conditioning test after optogenetic activation of Cbln2+ ICx-dPAG pathway (*n* = 6 mice per group).
(i) Intersectional anterograde tracing of ICx-dPAG neurons showing axonal projections in the anterior hypothalamic nucleus (AHN).
(j) Quantification of CPA performance in control and dPAG-projecting Cbln2+ ICx neurons-ablated mice (*n* = 7 mice per group).
(k) Representative ECG traces (left) and diaphragm EMG traces (right).
(l) Quantification of maximal heart rate before, during (Stimuli), and after 17–20 kHz sound stimulation (*n* = 3 mice, 9 trials per group).
(m) Quantification of maximal breathing rate before, during (Stimuli), and after 17–20 kHz sound stimulation (*n* = 3 mice, 9 trials per group).
(n) Schematic of fiber photometry recording from dPAG-projecting Cbln2+ ICx neurons.
(o) Representative coronal section showing GCaMP6s expression and optical fiber placement in the ICx. Scale bar, 200 μm.
(p) Heatmaps of Ca2+ activities of dPAG-projecting Cbln2+ neurons evoked by 17–20 kHz sound stimulation.
(q) Average GCaMP response curve and quantitative analyses for dPAG-projecting Cbln2+ neurons in response to various sound stimuli (duration, 2 s, 80 dB), *n* = 5 mice.
(r) Heatmaps of Ca2+ activities of dPAG-projecting Cbln2+ neurons evoked by 1–4 kHz, 33–36 kHz, and 57–60 kHz sound stimuli, *n* = 5 mice.
(s) Quantification of peak ΔF/F responses to the indicated sound stimuli.
All graphs are represented as mean ± SEM, *p < 0.05, **p < 0.01, ***p < 0.001 or ****p < 0.0001. For detailed statistical information, see Supplementary Table 4.

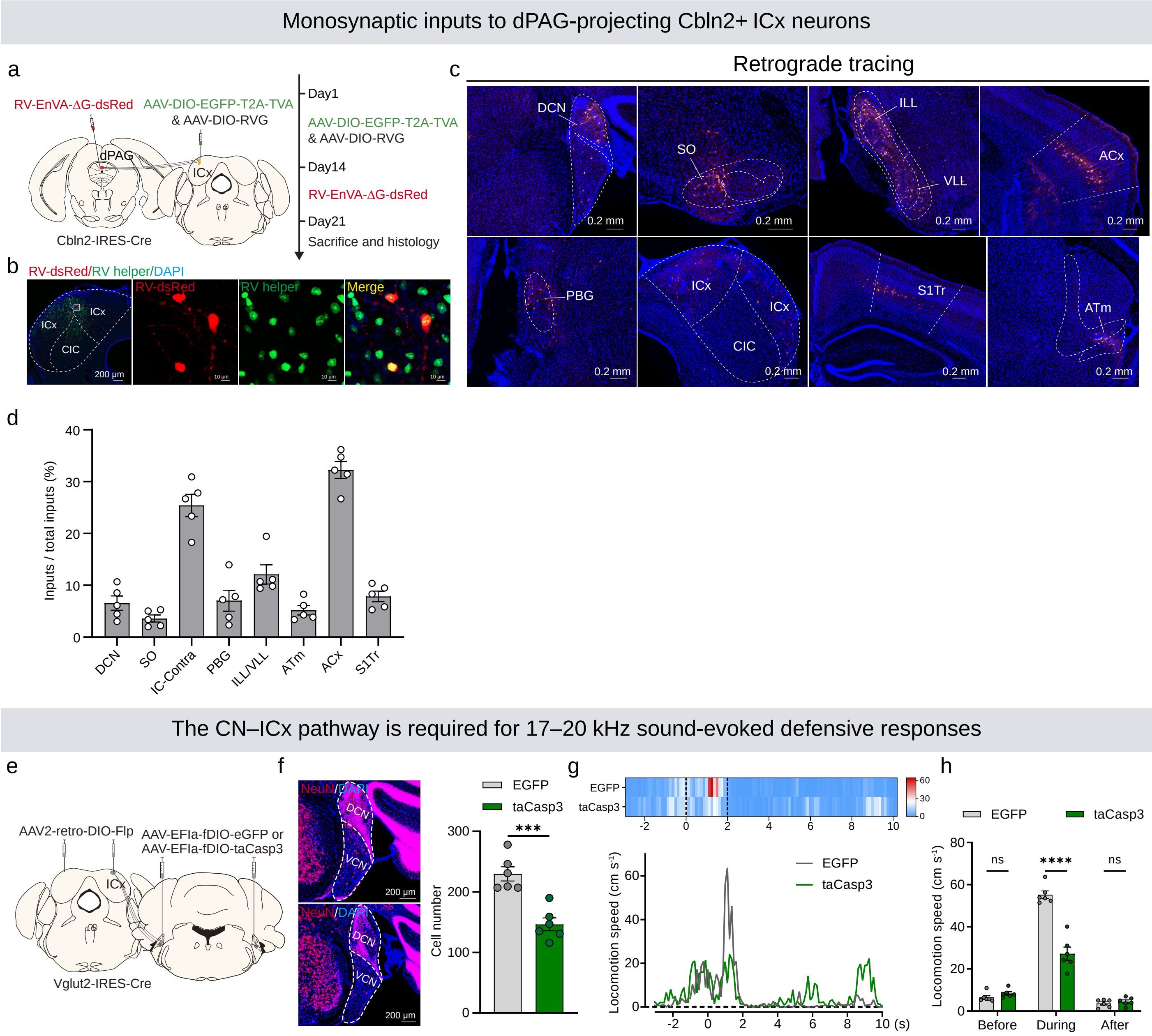

Figure S11. Monosynaptic inputs to dPAG-projecting Cbln2+ ICx neurons and the functional contribution of the CN–ICx pathway to sound-evoked defensive behavior.

(a) Schematic diagram showing the viral injection strategy for the retrograde tracing of dPAG-projecting Cbln2+ ICx neurons using a combination of AAV and rabies virus (RV).

(b) Example coronal brain section (left) and magnified area (right) showing starter cells in ICx of Cbln2-IRES-Cre mice.

(c) Example coronal sections showing rabies-labeled monosynaptic inputs to dPAG-projecting Cbln2+ ICx neurons in the indicated brain regions.

(d) Fractions of total RV-labeled cells in different brain regions monosynaptically projecting to the Cbln2+ ICx-dPAG pathway neurons (n = 5 mice). Note that the data in (d) were normalized by dividing by the total number of dsRed+ cells in these brain regions. Medial part of the auditory thalamus (ATm), dorsal cochlear nucleus (DCN),
superior olive (SO), intermediate nucleus of the lateral lemniscus (ILL), ventral nucleus of the lateral lemniscus (VLL), parabigeminal nucleus (PBG), primary somatosensory cortex (S1Tr).

(e) Schematic of the viral strategy used to selectively ablate glutamatergic CN neurons projecting to the ICx of Vglut2-IRES-Cre mice.

(f) Typical images of taCasp3-induced apoptosis in ICx-projecting CN glutamatergic neurons (left), and quantification of surviving cells (right).

(g) Locomotor-speed heatmaps and averaged traces during 17–20 kHz sound stimulation in control and taCasp3 mice.

(h) Quantification of locomotor speed before, during, and after 17–20 kHz sound stimulation (n = 6 mice per group)

All graphs are represented as mean ± SEM, ***$p < 0.001$, ****$p < 0.0001$. For detailed statistical information, see Supplementary Table 4.

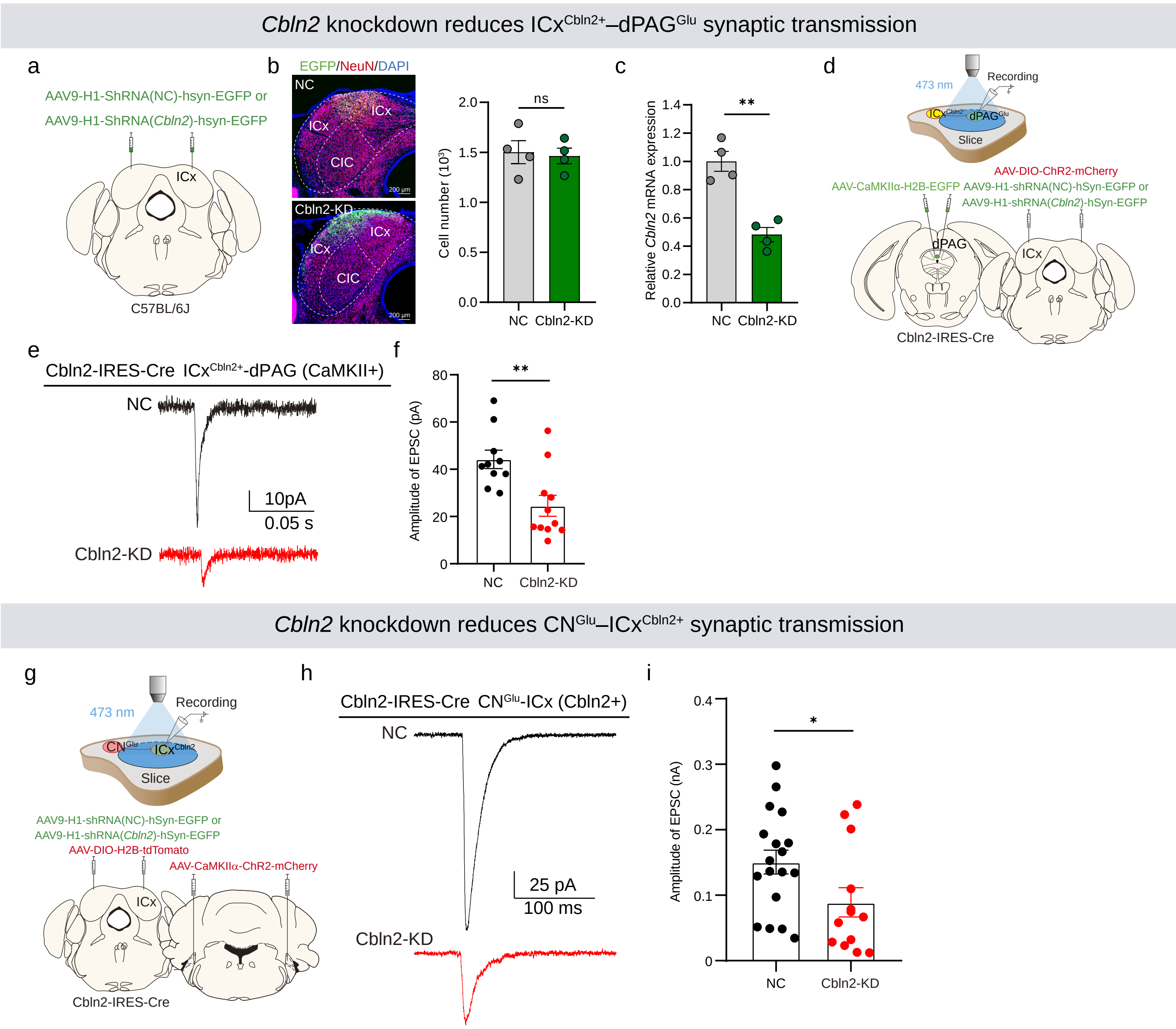

Figure S12. *Cbln2* knockdown weakens both input and output synaptic transmission of Cbln2+ ICx neurons.
(a) Schematic diagram showing the viral injection for *Cbln2* knockdown in the ICx of mice.
(b) Representative images of NeuN immunostaining in the ICx of NC and Cbln2-KD mice (left) and quantification of NeuN+ neurons (right, n = 4 mice per group).
(c) RT-qPCR analysis showing reduced *Cbln2* mRNA expression in the ICx following *Cbln2* knockdown (n = 4 mice per group).
(d) Schematic diagram showing virus injection and recording configuration in acute slices of Cbln2-IRES-Cre mice for assessing Cbln2+ ICx–dPAG synaptic transmission following *Cbln2* knockdown.
(e) Representative optogenetically evoked EPSC traces recorded from dPAG EGFP+ glutamatergic neurons in the NC and Cbln2-KD groups.
(f) Quantification of peak EPSC amplitudes (NC, n = 10 cells from 4 mice; Cbln2-KD, n = 11 cells from 5 mice).
(g) Schematic diagram showing virus injection and recording configuration in acute slices of Cbln2-IRES-Cre mice used to examine CN^{Glu}–Cbln2+ ICx synaptic transmission following *Cbln2* knockdown.
(h) Representative optogenetically evoked EPSC traces recorded from Cbln2+ ICx neurons in the NC and Cbln2-KD groups.
(i) Quantification of peak EPSC amplitudes showing that *Cbln2* knockdown significantly reduced CN–Cbln2+ ICx excitatory synaptic transmission (NC, n = 18 cells from 5 mice; Cbln2-KD, n = 13 cells from 5 mice).
All graphs are represented as mean ± SEM, $*p < 0.05$, $**p < 0.01$. For detailed statistical information, see Supplementary Table 4.
