# Supplementary material for: A Molecularly Defined Midbrain Circuit Mechanism for Sound‐Driven Defensive Behavior and Anxiety
Source: Adv Sci (Weinh). 2026 Sep 27:e77983. Online ahead of print. doi: 10.1002/advs.77983 (PMC13616374; doi:10.1002/advs.77983)
Supplement: Supplementary file 2 [file ADVS-9999-e77983-s005.docx]

**Supplementary information**

**Supplementary movie legends**

**Supplementary movie S1. 17–20 kHz** **sound-evoked defensive behavior**, related to Figure 1. Movie S1 shows defensive behavior evoked by 17–20 kHz sound in a representative WT mouse, including camera-recorded behavior (top), the open-field test, and the movement trajectory.

**Supplementary movie S2. Optogenetic activation of Cbln2+ ICx neurons**, related to Figure 4. Movie S2 shows that optogenetic activation of Cbln2+ ICx neurons using 20-Hz light pulses at 40 mW induces escape behavior in a representative mouse expressing AAV-DIO-ChR2-mCherry in Cbln2+ ICx neurons and implanted with an optical fiber above the ICx (right).

**Supplementary movie S3. Optogenetic activation of Cck+ ICx neurons**, related to Figure 4. Movie S3 shows that optogenetic activation of Cck+ ICx neurons using 20-Hz light pulses at 40 mW induces escape behavior in a representative mouse expressing AAV-DIO-ChR2-mCherry in Cck+ ICx neurons and implanted with an optical fiber above the ICx (right).

**Supplementary movie S4. GCaMP signal of Cbln2+ ICx neurons during 17–20 kHz acoustic stimulation,** related to Figure 5. Movie S4 shows fiber photometry recording of the GCaMP signal in Cbln2+ ICx neurons during 17–20 kHz-evoked escape behavior in a representative mouse. The GCaMP signal was temporally correlated with 17–20 kHz-evoked escape behavior.

**Supplementary movie S5. Optogenetic activation of the Cbln2+ ICx-dPAG pathway**, related to Figure 6. Movie S5 shows escape behavior induced by optogenetic activation of the Cbln2+ ICx-dPAG pathway (right) using 20-Hz light pulses at 40 mW in a representative mouse expressing AAV-DIO-ChR2-mCherry in Cbln2+ ICx neurons and implanted with an optical fiber above the dPAG (right).
