# Supplementary material for: A Molecularly Defined Midbrain Circuit Mechanism for Sound‐Driven Defensive Behavior and Anxiety
Source: Adv Sci (Weinh). 2026 Sep 27:e77983. Online ahead of print. doi: 10.1002/advs.77983 (PMC13616374; doi:10.1002/advs.77983)
Supplement: Supplementary file 3 [file ADVS-9999-e77983-s007.docx]

## Animals

All experimental procedures were conducted following protocols approved by the Administrative Panel on Laboratory Animal Care at Guangzhou laboratory (Guangzhou, China). Male wild-type (WT) C57BL/6 mice aged 8–14 weeks were obtained from Guangdong GemPharmatech Co., Ltd (Foshan, China). Cbln2-IRES-Cre, Cck-IRES-Cre, Vglut2-IRES-Cre, Vgat-IRES-Cre, and FosTRAP2 mouse lines were obtained from the Jackson Laboratory (JAX Mice and Services). Mice were housed at room temperature (23 ± 1 °C) with a stable humidity (50 ± 5%) on a 12-h light/12-h dark cycle, with ad libitum access to food and water. Mice were at least 8 weeks old at the time of viral injection. After viral injection, mice were singly housed for 3 weeks before subsequent experiments. Only male mice were used in this study.

## Stereotaxic injection of viral vectors

**Viral vectors.** Four adeno-associated virus (AAV) serotypes (AAV2/9, AAV2/1, AAV2/8, and AAV2-retro) were used. The AAVs and rabies virus (RV) used in this study are listed in Supplementary Table S1. Viral particles were purchased from Shanghai Taitool Bioscience Inc. and Brain VTA Inc. The titers of viral vectors were initially in the range of 0.8–1.5 × 10^13^ vg/mL. The final titer used for AAV injection was 2 × 10^12^ vg/mL after dilution with PBS.

**Stereotaxic injection.** Stereotaxic injections were performed as previously described ^1^. Mice were anesthetized with an intraperitoneal injection of tribromoethanol (125–250 mg/kg). After exposing the skull, small craniotomies were made above the target regions. Coordinates were as follows (AP from bregma, ML from midline, DV from dura): ICx, AP −5.0 mm, ML ±1.20 mm, DV −0.30 mm; CIC, AP −5.0 mm, ML ±1.20 mm, DV −0.70 mm; dPAG, AP −4.16 mm, ML ±0.50 mm, DV −2.05 mm, 8° angle; CN, AP −5.80 mm, ML 2.15 mm, DV −3.15 mm; SO, AP −5.40 mm, ML 1.10 mm, DV −4.75 mm; MGB, AP −3.00 mm, ML 1.85 mm, DV −2.85 mm; and ACx, AP −3.00 mm, ML 4.00 mm, DV −0.60 mm. A total of 200 nL of AAV vectors was delivered through a glass pipette connected to a NanoLiter Injector 201 (World Precision Instruments) at a flow rate of 0.15 μL/min. The pipette was left in place for at least 20 min after injection before withdrawal.

## Optic fiber implantation

Thirty minutes after AAV injections, a ceramic ferrule with an optic fiber (optogenetics, 200 μm in diameter, N.A. 0.22; fiber photometry, 200 μm in diameter, N.A. 0.35) was unilaterally or bilaterally implanted with the fiber tip positioned above the ICx (bregma −5.0 mm, lateral 1.20 mm, and dura −0.10 mm), CIC (bregma −5.0 mm, lateral 1.20 mm, and dura −0.50 mm), dPAG (bregma −4.16 mm, lateral 0.50 mm, and dura −1.85 mm, 8° angle), SPF (bregma −2.30 mm, lateral 0.4 mm, and dura −3.55 mm, 4° angle), ATm (bregma −3.00 mm, lateral 1.9 mm, and dura −3.2 mm), CnF (bregma −5.70 mm, lateral 1.2 mm, and dura −2.15 mm, 16° angle), MGB (bregma −3.00 mm, lateral 1.85 mm, and dura −2.65 mm), CN (bregma −5.80 mm, lateral 2.15 mm, and dura −2.95 mm), SO (bregma −5.40 mm, lateral 1.1 mm, and dura −4.55 mm), ACx (bregma −3.00 mm, lateral 4.0 mm, and dura −0.4 mm). Ferrules were secured with dental cement, and the surgical site was closed with sutures and treated with topical antibiotics. Optogenetic and fiber-photometry experiments were performed at least 3 weeks after fiber implantation. For optogenetic experiments, laser output at the fiber tip was calibrated to 10, 20, or 40 mW before each session. Photostimulation parameters, including pulse duration and frequency, were controlled by a programmable pulse generator coupled to the laser system.

## Cell-type-specific retrograde or anterograde tracing.

A modified RV-based three-virus system was used for mapping whole-brain inputs to dPAG-projecting Cbln2+ ICx neurons ^2^. All viruses, including AAV2/9-EF1α-DIO-EGFP-2A-TVA (5 x 10^12^ viral vg/mL), AAV2/9-EF1α-DIO-RG (5 x 10^12^ viral vg/mL), and EnVA-pseudotyped, glycoprotein (RG)-deleted and dsRed-expressing RV (RV-EnVA-dsRed, RV) (5.0 x 10^8^ viral vg/mL), were packaged and provided by BrainVTA Inc. (Wuhan, China). The Cre-dependent helper viruses (AAV2/9-EF1α-DIO-EGFP-2A-TVA and AAV2/9-EF1α-DIO-RG, 1:1 mixed) were injected into the ICx of Cbln2-IRES-Cre mice unilaterally. Two weeks after AAV helper injection, RV-EnVA-ΔG-dsRed was injected into the dPAG of Cbln2-IRES-Cre mice in a biosafety level-2 lab facility. Starter neurons were characterized by the co-expression of dsRed and EGFP, which were restricted to the ICx. One week after injection of RV, mice were perfused with saline, followed by 4% paraformaldehyde (PFA) in phosphate-buffered saline (PBS). Brains were post-fixed in 4% PFA for 2 h and then incubated in 30% sucrose in PBS until they sank to the bottom. Coronal brain sections (40 μm thick) were prepared using a cryostat (Leica CM1900). All sections were collected and stained with 4′,6-diamidino-2-phenylindole (DAPI). The sections were imaged with an Olympus VS120 epifluorescence microscope (10× objective). Images were analyzed in ImageJ. Regional boundaries were defined according to the Allen Brain Atlas, and the fractional distribution of RV-labeled cells across brain regions was quantified.

For cell-type-specific anterograde tracing of Cbln2+ or Cck+ ICx neurons, AAV-hSyn-DIO-mGFP-T2A-Synaptophysin-mRuby was stereotaxically injected into the ICx of Cck-IRES-Cre or Cbln2-IRES-Cre mice. Three weeks later, mice were perfused and brains were processed as described above. Coronal brain sections (40 μm thick) were prepared using a cryostat (Leica CM1900), immunostained for mCherry, counterstained with DAPI, and imaged using an Olympus VS200 microscope (10× objective). For quantification of downstream projections, the fluorescence intensity of synaptophysin-mRuby-labeled axon terminals in each target region was measured in ImageJ and normalized to the summed signal across quantified regions. Regional boundaries were defined using the Allen Brain Atlas.

## FosTRAP experiments

AAV-EF1α-DIO-ChR2-mCherry was unilaterally injected into the ICx of FosCreER (Fos-2A-iCreER) mice for 17–20 kHz or 33–36 kHz sound-TRAP experiments, whereas the CIC was targeted only in the 33–36 kHz control group. Tamoxifen was dissolved in corn oil containing 10% anhydrous ethanol at a final concentration of 30 mg/mL. On day 7, after recovery from intraoral cheek-fistula surgery, mice were placed in their home cages inside a soundproof chamber and allowed to acclimate for 12 h. Mice were then exposed to ultrasonic stimulation at either 17–20 kHz or 33–36 kHz (2 s on, 2 s off, 3 cycles, followed by a 1 min interstimulus interval) for 30 min. Thirty minutes after the end of acoustic stimulation, tamoxifen (150 mg/kg) was delivered to the FosCreER mice via intraoral solution delivery through the pre-implanted cheek fistula. On day 28 (3 weeks after sound-TRAP), mice were re-exposed to the same sound stimulus and perfused 1.5 h later. Coronal sections (40 μm) were collected for immunohistochemical analysis of mCherry and c-Fos.

## RNA in situ hybridization

RNA in situ hybridization was performed as described previously, with modifications for combined immunofluorescence labeling. Mice were perfused with 0.9% sodium chloride (Guangzhou Chemical Reagent Factory), followed by 4% PBS-buffered paraformaldehyde (PBS-PFA). Brains were post-fixed in 4% PBS-PFA solution 2 h and then incubated in 30% sucrose in PBS at 4 °C until they sank to the bottom. Cryostat sections (12-μm thick) were prepared and collected on the glass slides. Fluorescence in situ hybridization (FISH) was performed as described as follows. Briefly, cryostat sections were rinsed with PBS for 5 min, and then placed in a dryer at 60 °C for 30 min. Brain sections were then fixed with 4% PBS-PFA solution for 15 min. The slides were sequentially immersed in a graded ethanol dehydration series (50%, 70%, and 100% anhydrous ethanol). Endogenous peroxidase activity was quenched by incubating slides with RNAscope hydrogen peroxide for 10 min at room temperature. For antigen retrieval, slides were immersed in boiling Co-Detection Target Retrieval solution (ACD, 323166) for 5 min, then rinsed with PBST. Sections were incubated with Cy2-conjugated anti-EGFP primary antibodies (1:600) diluted in Co-Detection Antibody Diluent (ACD, 323160). This process was conducted in a humidified dark chamber at 4 °C for 24 h. Following PBST washes, the tissue was post-fixed with 4% PFA for 30 min at room temperature. Subsequently, Protease Plus was applied to the sections for 30 min at 40 °C to facilitate probe penetration. Digoxigenin-labeled mouse anti-sense cRNA probes for *Cbln2* were hybridized to the sections at 40 °C for 2 h. Signal amplification was performed using the RNAscope Multiplex Fluorescent kit; specifically, AMP1, AMP2, and AMP3 reagents were applied sequentially, with each incubation lasting 30 min at 40 °C. Between steps, slides were rinsed with 1× Wash Buffer (ACD, 320058). HRP-labeled probes for *Cbln2* were applied for 15 min at 40 °C, followed by incubation with TSA-buffered secondary antibodies (1:15) for 30 min. After treating with HRP blocker for 15 min, EGFP signals were further enhanced using Alexa Fluor® 488-conjugated goat anti-rabbit secondary antibodies (1:500) for 30 min at room temperature. Finally, fluorescence was visualized using an Olympus VS 200 microscope (10× objective) or a Zeiss 980 confocal microscope (60× oil-immersion objective).

## Slice electrophysiology recording.

Acute brain slices containing the ICx were prepared according to a previously described protocol ^1^. Briefly, adult mice were anesthetized with isoflurane and decapitated. The brains were rapidly removed and immersed in ice-cold, oxygenated (95% O_2_ and 5% CO_2_) sucrose-based cutting solution (CS: 228 mM sucrose, 11 mM glucose, 26 mM NaHCO_3_, 1 mM NaH_2_PO_4_, 2.5 mM KCl, 7 mM MgSO_4_, and 0.5 mM CaCl_2_). Coronal sections (250-μm thick) were obtained using a Vibratome (Leica VT 1200S). The slices were incubated in artificial cerebrospinal fluid (ACSF) at 28 °C for 30 min and subsequently maintained at room temperature for at least 1 h prior to recording. The ACSF composition was: 119 mM NaCl, 2.5 mM KCl, 1 mM NaH_2_PO_4_, 1.3 mM MgSO_4_, 26 mM NaHCO_3_, 10 mM glucose, and 2.5 mM CaCl_2_. The ACSF was perfused at 1 mL/min. The acute brain slices were visualized using an Olympus 40× water-immersion objective equipped with differential interference contrast optics (Olympus Inc., Japan) and a CCD camera. Patch pipettes (3.0–3.5 MΩ) were pulled from borosilicate glass capillary tubes (Cat. no. 64-0793, Warner Instruments, Hamden, CT, USA) using a PC-10 pipette puller (Narishige Inc., Tokyo, Japan). For current-clamp recordings of action potentials from ChR2-mCherry⁺ ICx neurons, pipettes were filled with internal solution (135 mM K-methanesulfonate, 10 mM HEPES, 1 mM EGTA, 1 mM Na-GTP, 4 mM Mg-ATP, pH 7.4). For voltage-clamp recordings of light-evoked postsynaptic currents in dPAG neurons, the membrane potential was held at −70 or 0 mV, and pipettes were filled with internal solution (135 mM CsCl, 10 mM HEPES, 1 mM EGTA, 2 mM QX-314 chloride, 1 mM Na-GTP, 4 mM Mg-ATP, pH 7.4). The current and voltage signals were recorded with MultiClamp 700B amplifier and Clampex 10 data acquisition software (Molecular Devices). After establishment of the whole-cell configuration and equilibration of the intracellular pipette solution with the cytoplasm, series resistance was compensated to 10–15 MΩ. Recordings with series resistance > 15 MΩ were excluded. Photostimulation was delivered through a 200-μm optical fiber positioned above the slice (473 nm, 20 mW). Light-evoked action potentials in ChR2-mCherry+ ICx neurons were tested using 1 ms pulses at 10 or 20 Hz. To map synaptic connections, light-evoked synaptic currents were recorded from EGFP+ glutamatergic neurons in the dPAG. These currents were elicited by single light pulses (1 ms) in the presence of TTX (1 μM) and 4-AP (20 μM) to isolate monosynaptic responses. To determine the neurotransmitter identity of inputs arising from ChR2-mCherry-expressing ICx neurons, the following antagonists were bath-applied in ACSF: D-AP5 (50 μM) and CNQX (20 μM) for glutamatergic transmission, and picrotoxin (50 μM) for GABAergic transmission.

For analysis of synaptic transmission following *Cbln2* knockdown, mice were injected with either shRNA (*Cbln2*) or control shRNA in the ICx. For recordings of Cbln2+ ICx–dPAG transmission, ChR2 was expressed in Cbln2+ ICx neurons, and whole-cell voltage-clamp recordings were obtained from EGFP+ glutamatergic neurons in the dPAG. Light-evoked EPSCs were elicited by single 473-nm light pulses (1 ms) while neurons were held at −70 mV. For recordings of glutamatergic CN–Cbln2+ ICx transmission, ChR2 was expressed in glutamatergic CN neurons, and EPSCs were recorded from H2B-tdTomato-labeled Cbln2+ ICx neurons following optical stimulation of CN axon terminals. Peak EPSC amplitudes were quantified and compared between the control and *Cbln2*-knockdown groups.

## RT-qPCR

To assess *Cbln2* knockdown efficiency, ICx tissues were collected from mice injected with shRNA (*Cbln2*) or control shRNA. Total RNA was extracted using the RNAsimple Total RNA Kit (TIANGEN, Cat. No. DP419) according to the manufacturer’s instructions. Then, 500 ng of total RNA from each sample was reversely transcribed using the HiScript III SuperMix for qPCR (Vazyme, Cat. No. R323-01). Quantitative real-time PCR was performed using Taq Pro Universal SYBR qPCR Master Mix (Vazyme, Cat. No. Q712-02). Relative *Cbln2* mRNA expression was normalized to *Gapdh* and calculated using the 2^−ΔΔCt^ method, with the mean ΔCt value of the shRNA (NC) group used as the reference. Primer sequences are provided in Supplementary Table S1.

## Immunohistochemical analysis.

Immunohistochemical staining was performed as previously described. Mice were anesthetized with isoflurane and sequentially perfused with saline followed by 4% PFA in PBS. Post-fixed brains were cryoprotected in 30% sucrose until equilibration. Coronal sections (40 μm) were obtained using a cryostat and subjected to blocking in PBS containing 10% goat serum and 0.7% Triton X-100. Subsequently, sections were incubated with primary antibodies (listed in Supplementary Table 1) overnight at 4 °C. After three rinses in washing buffer (PBS with 0.7% Triton X-100), sections were incubated with fluorophore-conjugated secondary antibodies (tagged with Cy2 or Cy3; dilution 1:500) for 2 h at room temperature. Sections were then washed three times with washing buffer, nuclei were counterstained with DAPI, then washed with PBS. Sections were transferred onto Superfrost Plus slides and mounted under glass coverslips with mounting medium. Sections were imaged with an Olympus VS 200 (10X objective ) or a Zeiss 980 laser scanning confocal microscope (60 oil-immersion objective lens). For confocal imaging, samples were excited sequentially with 488-, 543-, and 633-nm lasers to minimize channel cross-talk. Images were analyzed using ImageJ.

## Cell-counting strategies

The cell-counting strategies are summarized in Table S3. For counting cells in the ICx, we collected coronal sections (40 or 12 μm) from bregma -4.9 to bregma -5.4 for each brain. Three sections spaced by 200 μm were sampled for immunohistochemistry or FISH to label cells positive for different markers. For whole-brain sampling, we collected coronal sections (40 μm) for each mouse. one section every 200 μm was taken for immunohistochemistry to label positive cells. Relative fluorescent intensities of mRuby+ or mCherry+ cell axon terminals were measured using ImageJ. We acquired micrographs (×10 objective) of retrogradely labeled nuclei and calculated the average of RV-dsRed+ neurons.

## Single-nucleus RNA sequencing

Five adult WT mice were anesthetized with isoflurane and brains were rapidly removed and immersed in ice-cold ACSF. The ACSF was pre-equilibrated with carbogen (95% O_2_ and 5% CO_2_) and contained: 119 mM NaCl, 2.5 mM KCl, 1 mM NaH2PO_4_, 1.3 mM MgSO_4_, 26 mM NaHCO_3_, 10 mM glucose, and 2.5 mM CaCl_2_. Coronal slices (250 μm) were prepared using a vibratome (VT 1200S, Leica Microsystems, Wetzlar, Germany) in ACSF. The IC was microdissected based on established anatomical landmarks, immediately immersed in RNAlater (Invitrogen), flash-frozen in liquid nitrogen, and stored at −80 °C. Single nuclei were isolated using a Nuclei Isolation Kit (Sigma-Aldrich, NUC201-1KT) according to the manufacturer’s instructions. Following isolation, the nuclei were resuspended in a chilled PBS solution supplemented with 1% BSA and 0.2 U/μL RNase inhibitor. The final nuclei concentration was adjusted to 800–1,000 nuclei/μL for library preparation.

snRNA-seq libraries were generated using Chromium™ Next GEM Single Cell 3’ GEM Library & Gel Bead Kit v3.1 (10x Genomics, Cat. no. 1000121) according to the manufacturer’s protocol. Libraries were sequenced on an Illumina NovaSeq 6000 v1.5 platform to a depth of at least 100,000 reads per nucleus.

## IC snRNA-seq data processing

Unique molecular identifier (UMI) counts were generated for each barcode using the cellranger count pipeline (Cell Ranger v5.0.1) with the --include-introns parameter ^3^. Count data were processed with the Seurat package (version 4.1.0) ^4^. Downstream analyses were performed in Seurat (v4.1.0). Doublets were identified and removed using DoubletFinder (v2.0.3) ^5^. For quality control, nuclei with 500 < nFeature_RNA < 6,000, 1,000 < nCount_RNA < 20,000, and percent.mt < 1 were retained. After quality control, nuclei from different samples were then merged by merge function, and gene expression counts were normalized with NormalizedData function, were scaled and centred using ScaleData function. Batch-effect correction was performed by Harmony (version 0.1.0) after principal component analysis ^6^. A shared nearest-neighbor graph was constructed based on Harmony embeddings, and clustering was performed using the SLM algorithm implemented in FindClusters. Clusters were visualized using Uniform Manifold Approximation and Projection (UMAP). Cluster marker genes were identified using FindAllMarkers, and cell types were assigned based on canonical markers. Specifically, clusters were annotated as excitatory neurons (Slc17a6), inhibitory neurons (Gad1, Gad2), oligodendrocytes (Mbp, Mog), oligodendrocyte precursor cells (Pdgfra), astrocytes (Aqp4, Gfap, Slc15a2), endothelial cells (Abcc9, Pecam1, Cdh5), microglia (Cx3cr1, Csf1r, Cd53), and ependymal cells (Spef2). Neuronal clusters were then reclustered at a resolution of 0.1. Gene ontology enrichment analysis of cluster marker genes was performed using Metascape ^7^.

## Fiber photometry

Calcium signals were recorded by a fiber photometry system (Thinker Tech Nanjing Biotech CO., Ltd.). AAV-CAG-DIO-GCaMP6s was stereotaxically injected into the ICx of Cbln2-IRES-Cre or Cck-IRES-Cre mice. AAV2-retro-DIO-GCaMP6s was stereotaxically injected into the dPAG of Cbln2-IRES-Cre mice. After viral injection, the optical fiber (200 μm core diameter, NA 0.35) was implanted above the ICx. A fiber photometry system (ThinkerTech) was used for recording GCaMP signals from genetically identified neurons. To induce fluorescence signals, a laser beam from a laser tube (470/10 nm, model 65144, Edmund Optics) was reflected by a dichroic mirror, focused by a 10 × (NA = 0.35) lens and coupled to an optical commutator. To minimize photobleaching, the power at the fiber tip was adjusted to 0.02 mW. GCaMP6s fluorescence was band-pass filtered (MF525-39, Thorlabs) and collected by a photomultiplier tube (R3896, Hamamatsu) ^8^. An amplifier (C7319, Hamamatsu) was used to convert the photomultiplier tube current output to voltage signals, which was further filtered through a low-pass filter (40 Hz cut-off; Brownlee 440). The analog voltage signals were digitized at 100 Hz and recorded by a Power 1401 digitizer and Spike2 software (CED, Cambridge, UK). Two weeks after AAV injection, fiber photometry was used to record GCaMP signals. A flashing light-emitting diode triggered by a 1-s square-wave pulse was simultaneously recorded to synchronize the video and GCaMP signals. Head-fixed mice standing on a treadmill in response to sound stimuli (1–4 kHz, 17–20 kHz, 33–36 kHz, or 57–60 kHz; 2 s, 80 dB). Fluorescence changes were expressed as ΔF/F = (F − F0)/F0, where F0 was defined as the mean baseline fluorescence during the period from −1 s to 0 s relative to stimulus onset. Peak ΔF/F was defined as the maximal ΔF/F value during the 2 s sound-stimulation period. After the experiments, the fiber placements in the ICx were histologically examined in each mouse.

## Head-fixed two-photon imaging

Animals aged 8 weeks were anesthetized with intraperitoneal injections of tribromoethanol (125–250 mg/kg, i.p.). The scalp was shaved and an incision was made, after which a circular craniotomy (3 mm in diameter) was performed over the ICx of Cbln2-IRES-Cre mice using a 0.4-mm drill bit. Following the unilateral injection of AAV-DIO-GCaMP6s into the left ICx, a circular cover glass of 2.5 mm diameter (N-buliv, LG18-402L-1) was placed onto the ICx and secured in place to the surrounding skull with glue (Filtek™, Z350XT). After a headplate was attached, a small water well was built around the window to allow immersion of the objective lens during imaging. Three weeks post-viral injection, in vivo calcium imaging was conducted using a two-photon laser-scanning microscope (Carl Zeiss LSM 980 NLO) equipped with a 40× water-immersion objective (0.8 NA, 3.5 mm working distance). The excitation laser was tuned to 920 nm, with the output power strictly maintained below 20 mW. During acoustic stimulation, time-series images of calcium activity within the ICx were recorded at a resolution of 256 × 256 pixels and a sampling rate of 4 Hz. To evaluate neuronal calcium dynamics, motion correction was initially performed using the EZcalcium-2.1.2 toolbox in MATLAB, followed by the quantification of somatic calcium signals via ImageJ software (National Institutes of Health) according to previous studies ^9, 10^. Regions of interest (ROIs) corresponding to individual neuronal somata were manually defined. Fluorescence intensity (F) was calculated as the mean pixel intensity within each ROI at each time point. The relative change in fluorescence (ΔF/F) was calculated as (F − F_0_)/F_0_, where F_0_ was the mean baseline fluorescence during the period from −5 to 0 s. Background fluorescence was subtracted before ΔF/F calculation. After imaging, the ICx region was verified histologically.

## Single-unit recording with optrode

An optrode was used to record and identify single-unit activity from Cbln2+ ICx neurons. AAV2/9-DIO-ChR2-mCherry was injected into the ICx of Cbln2-IRES-Cre mice. Three weeks after injection, single-unit recordings were performed in the ICx of head-fixed, awake mice standing on a treadmill. The single-channel optrode was constructed by aligning an optical fiber parallel to a glass-coated tungsten electrode (impedance: 1–3 MΩ), with a tip-to-tip distance of approximately 200 μm. To minimize tissue damage, the optrode was inserted vertically into the ICx via a micromanipulator, positioning the tungsten electrode tip within the ICx while maintaining the optical fiber above the target region. Spike signals were amplified using a differential amplifier (Model 1800, A-M Systems), digitized at a sampling rate of 10 kHz, and recorded using Spike2 software (Version 7.03). Upon isolation of spikes from a sound (17–20 kHz) response unit, a pulse train (10 Hz, 1 s) was delivered to verify whether the unit originated from ChR2-expressing neurons. Putative Cbln2+ neurons were identified based on two criteria: a light-evoked response latency <5 ms and high waveform similarity between light-evoked and sound-evoked spikes. Units meeting both criteria were subjected to further sound-stimulation testing. Spike sorting was subsequently conducted using Spike2 software (Version 7.03), consistent with previously established protocols ^11^.

## Behavioral testing procedures

After AAV injection and fiber implantation, mice were housed individually for 3 weeks before the behavioral tests. Mice were handled daily for at least 3 days before testing. On the test day, cages were transferred to the behavioral room and mice were habituated to the room for 3 h before experiments began. Apparatus was cleaned with 20% ethanol to eliminate odor cues from other mice. Behavioral tests were performed in the same circadian period (1 PM–7 PM). All behavioral analyses were performed by experimenters blind to group allocation.

## Auditory stimulation and behavioral tests

Mice were transferred from their home cages to the testing open-top chamber (35 × 35 × 15 cm) and permitted to habituate for 10 minutes. An ultrasonic speaker (Vifa, Avisoft) placed 15 cm above the floor of the chamber. The stimulus consisted of a frequency-modulated upsweep (1–4 kHz, 17–20 kHz, 33–36 kHz, 57–60 kHz; linearly increasing over 100 ms) with a total duration of 2 s and an intensity of 80 dB, delivered via a power amplifier (ATA-3090, Aigtek Electronic Technology). The order of the four sound frequencies was randomized within each mouse. For sound-intensity experiments, mice were presented with 17–20 kHz FM sweeps at 50, 60, 70, 80, or 90 dB, and the order of sound intensities was randomized within each mouse. Frequency-comparison and intensity-comparison experiments were performed in two independent groups of mice. Sound intensity was calibrated using an Avisoft ultrasonic recording system. Sounds emitted from the speaker were recorded and the sound level was measured in BatSound software by adjusting amplifier output power. Stimuli were presented with an inter-trial interval of approximately 2 minutes. Mouse locomotion was recorded with an overhead camera and analyzed using a video tracking system (VisuTrack XR-VT, Xinruan Information Technology, China). For quantification of sound-evoked locomotor responses, each sound stimulus was delivered once per mouse. Locomotor speed was quantified in three time windows: before, during, and after stimulation. “Before” was defined as the mean locomotor speed during the 3 s preceding stimulus onset, “during” as the maximal locomotor speed during the 2 s stimulation period, and “after” as the mean locomotor speed during the 15 s following stimulus offset.

## Optogenetic activation and defensive behavior assays

After a 3-week recovery period following stereotaxic injection of AAV into the ICx and optical fiber implantation above the ICx, SPF, CnF, ATm, or dPAG, mice were handled for 3 days before behavioral testing. A series of escape behavior assays were conducted over five consecutive days within a testing chamber (30 × 40 × 30 cm). These assays were designed to evaluate behavioral responses to different stimulation parameters, including laser power (10/20/40 mW), frequency (2/20 Hz), duration (0.5/5 s), and the adaptation to repetitive photostimulation. To deliver light stimulation, the implanted optical fiber was connected to a 473-nm laser tube (DPSSL Driver) through an FC/PC adaptor. Laser pulses were controlled by a pulse generator (RWD), and the optical power at the fiber tip was calibrated prior to the experiments. Locomotor activity was recorded from an overhead camera, and behavioral data were subsequently processed using a video tracking system for offline analysis (VisuTrack XR-VT, Xinruan Information Technology, China). For optogenetic escape assays, each photostimulation parameter was delivered once per mouse. Locomotor speed was quantified as the mean speed during the 5 s before stimulation (before), the maximal speed during photostimulation (during), and the mean speed during the 15 s after stimulation (after).

## Heart rate and diaphragm EMG recording

Following the induction of anesthesia with tribromoethanol (125–250 mg/kg, i.p.), an abdominal incision was performed to expose the diaphragm. A bipolar tungsten microelectrode (A-M Systems, No. 795500), with approximately 0.2 mm of insulation removed from the tips, was inserted into the left costal region of the diaphragm using a syringe needle as a guide. Concurrently, unipolar tungsten microelectrodes were implanted subcutaneously in the right forelimb and lower left abdomen for heart rate monitoring. These microelectrodes were then tunneled percutaneously, and the connectors for electromyogram (EMG) and heart rate (HR) recording were securely fixed to the skull. Physiological signals were amplified using a Model 1800 Microelectrode AC Amplifier (A-M Systems) and band-pass filtered at 100–5000 Hz for EMG recordings and 100–500 Hz for HR recordings. Signals were digitized and recorded in Spike2 software (CED). For each sound or photostimulation condition, stimulation was repeated three times per mouse. Heart rate was calculated as beats per second, and respiratory rate was calculated as breaths per second. For 17–20 kHz sound stimulation, “before” was defined as the maximal rate during the 3 s preceding stimulus onset, “during” as the maximal rate during stimulation, and “after” as the maximal rate during the 15 s following stimulus offset. For optogenetic responses, “Pre” was defined as the maximal rate during the 5 s preceding photostimulation onset, “Laser” as the maximal rate during the 5 s photostimulation period, and “Post” as the maximal rate during the 15 s following photostimulation offset.

## Pupil measurement

Mice were habituated to a constant ambient light environment for 1 h and were then head-fixed under infrared illumination for pupil recording. Under constant lighting conditions, videos were acquired during the pre-stimulus, stimulus, and post-stimulus periods in response to light or acoustic stimulation. Pupil size was quantified frame by frame using VisuTrack XR-VT (Xinruan Information Technology, China), and pupil responses were expressed as normalized pupil area. For each sound or photostimulation condition, stimulation was repeated three times per mouse. For sound-evoked responses, “before” was defined as the mean normalized pupil area during the 3 s preceding stimulus onset, “during” as the maximal normalized pupil area during stimulation, and “after” as the mean normalized pupil area during the 15 s following stimulus offset. For optogenetic responses, “Pre” was defined as the mean normalized pupil area during the 5 s preceding photostimulation onset, “Laser” as the maximal normalized pupil area during the 5 s photostimulation period, and “Post” as the mean normalized pupil area during the 15 s following photostimulation offset.

## Repeated sound exposure-induced anxiety-like behavior

Mice were randomly assigned to the silent control, 33–36 kHz sound, or 17–20 kHz sound group. Mice were exposed to the corresponding acoustic stimulus for 1 h per day for 3 consecutive days. Each stimulation consisted of three cycles of 2 s sound-on and 2 s sound-off, followed by a 1-min interval. Mice in the silent control group were placed in the same chamber for an equivalent duration without sound stimulation. Immediately after the final exposure on day 3, anxiety-like behaviors were assessed using the open field test (OFT) and elevated plus maze (EPM).

For chemogenetic inhibition of the Cbln2+ ICx–dPAG pathway, AAV2-retro-DIO-Flp was injected into the dPAG, and AAV-fDIO-hM4Di or AAV-fDIO-mCherry control virus was injected into the ICx of Cbln2-IRES-Cre mice. After viral expression, mice were randomly assigned to silent or 17–20 kHz sound-exposure groups. During the 3-day exposure paradigm, clozapine-N-oxide (CNO; 1 mg/kg, i.p.) was administered 30 min before each daily session. Mice were then exposed to 17–20 kHz sound for 1 h using the same stimulation protocol described above, whereas silent controls remained in the same chamber for 1h without sound stimulation. Immediately after the final session on day 3, anxiety-like behaviors were assessed using the OFT and EPM. After behavioral testing, mice received an intraperitoneal injection of CNO 30 min before being re-exposed to 17–20 kHz sound. Mice were perfused 90 min after sound stimulation, and brain sections were collected for histological verification of viral expression and c-Fos analysis.

## Open field test

To assess whether repeated activation of the ICx–dPAG pathway induced anxiety-like behavior, mice received unilateral photostimulation once daily for 3 consecutive days. Each daily stimulation session lasted 1 h and consisted of 5 s on / 5 s off trains (20 Hz, 10 ms pulse width, 10 mW laser output). Behavioral testing was performed after the third stimulation session. Mice were placed individually in one corner of an open-field arena (50 × 50 × 30 cm) and allowed to explore freely for 5 min immediately after the third stimulation session. Locomotor trajectories were recorded with an overhead camera. The center zone was defined as the central 25 × 25 cm square, and the time spent in this region was quantified offline using VisuTrack XR-VT (Xinruan Information Technology, China). The apparatus was cleaned with 75% ethanol between animals.

## Elevated plus maze

The elevated plus maze (EPM) apparatus consisted of a central platform (5 × 5 cm), two opposite open arms (30 × 5 cm), and two perpendicular closed arms (30 × 5 × 20 cm), with the entire structure positioned 70 cm above the floor. Each mouse was placed individually on the central platform facing a closed arm and allowed to explore freely for 5 min. Locomotor trajectories were recorded with an overhead camera. Time spent in the open arms was quantified offline through automated tracking software (VisuTrack XR-VT, Xinruan Information Technology, China). To eliminate residual olfactory cues, the entire apparatus was thoroughly cleaned with 75% ethanol between consecutive tests.

## Conditioned Place Aversion (CPA)

Optogenetic activation CPA assays were conducted in a two-compartment apparatus (25 × 25 cm per chamber). The two chambers were distinguished by wall patterns (horizontal vs. vertical black-and-white stripes) and floor textures (smooth vs. rough). All phases were conducted under sound-attenuated conditions. The CPA paradigm consisted of three consecutive phases: a baseline preference test (pre-test, Day 1), a conditioning phase (Day 2), and an aversion test (post-test, Day 3). During the pre-test, mice were allowed to explore the entire apparatus freely for 15 min, and animals with a strong innate chamber bias (>70%) were excluded from subsequent experiments. During the conditioning phase, animals were allowed to freely move between the chambers for 20 minutes, optical stimulation (473 nm, 10 ms pulse duration, 20 Hz, 0.2 s duration) was triggered when the mouse entered the designated conditioned chamber and stopped when it exited. During the post-test, mice were again allowed to explore both chambers freely for 15 min. Time spent in each chamber was quantified to assess conditioned place aversion. Behavior was recorded by overhead cameras and analyzed using VisuTrack XR-VT.

For sound-induced CPA experiments, a similar two-compartment apparatus (20 × 15 cm per chamber) was employed, utilizing distinct visual (horizontal vs. vertical stripes) and tactile (smooth vs. rough floors) cues to differentiate the two chambers. The experimental timeline was the same as that used for optogenetic CPA and included pre-test, conditioning, and post-test phases. During the 15 min pre-test, mice were allowed to explore both chambers freely and animals with strong chamber bias were excluded. During this 20 min conditioning period, a 17–20 kHz stimulation (2 s on, 2 s off) was triggered when the mouse entered the conditioned chamber and stopped when it exited. In the non-conditioned chamber, 33–36 kHz sound stimulation (2 s on, 2 s off) was delivered. During the post-test, mice explored both chambers freely for 15 min. Time spent in each chamber was quantified to assess sound-induced conditioned aversion.

## Cue-associated freezing behavior test

Three weeks after stereotaxic AAV injection, adult male mice were tested using the XR-XC404 fear-conditioning system (XinRuan Shanghai Instrument). Each mouse was placed in an operant box (cuboid, 28 × 28 × 52 cm) located in a soundproof cubicle and allowed to explore freely for 2 min. The conditioned stimulus (CS+) consisted of 30 s trains of frequency-modulated sweeps (0.5 s duration, logarithmic upsweeps from 1 to 15 kHz, 50 ms rise time) delivered at 1 Hz and 70 dB. The CS+ was paired with a foot shock (1 s, 0.6 mA, 15 CS+–foot-shock pairings; inter-trial interval: 30 s). The frequency-modulated sweep stimulus was generated by a waveform generator (Cat. no. DG1022Z, Rigol), amplified by a power amplifier (ATA-3090, Aigtek Electronic Technology), and delivered through an ultrasonic speaker (Vifa, Avisoft) positioned 15 cm above the floor. After 24 h, mice were tested for fear retrieval in an altered context for 5 min, followed by a 2 min CS+ presentation to assess cue-evoked freezing. To create the altered context, the metal grid floor was replaced with a plastic sheet, red stripes were added to the white plastic walls, and 3% acetic acid replaced 20% ethanol as the contextual odor cue.

## Photostimulation-based fear conditioning

The modified fear conditioning experiment (light stimulation instead of foot shock) was conducted over two consecutive days. During the training session, mice were placed in a conditioning chamber located in a sound-attenuated cubicle. The chamber was cleaned with 20% ethanol to establish olfactory background, while the ventilation fan provided a background noise of approximately 50 dB. After 2 min free exploration period, three 30 s tones (2 kHz, 70 dB) co-terminated with light stimulation (pulse duration 20 ms at 20 Hz for 2 secs) separated by 1-min intervals were delivered. Upon completion of the paired training, the animals remained in the chamber for an additional 30 s before being returned to their home cages. The fear memory retrieval session consisted of three sequential tests: a contextual test, an altered context test, and a tone test. For the context test, mice were placed back into the original conditioning chamber for 5 min to quantify freezing. Subsequently, the animals were transferred to the altered chamber for 5 min to evaluate freezing in the altered context, followed by a 1 min tone presentation (2 kHz, 70 dB) to assess cue-dependent fear retrieval. The altered context test was performed in a separate room. The chamber for the altered context was modified by replacing the metal grid floor with plastic sheeting, and the square plastic walls featuring black-and-white stripes were substituted with a white cylindrical enclosure. Additionally, the odor cue was changed from ethanol to acetic acid, and the ventilation fan was turned off.

Manufacturers of the instruments used in this study

| **Instrument name** | **Manufacturer** |
| --- | --- |
| optogenetic laser | RWD |
| Brain Stereotactic Injector | RWD |
| Data Acquisition System | CED |
| Fiber photometry | ThinkerTech Nanjing Bioscience Inc |
| Axon Digidata 1550B Low Noise Data Acquisition System | Axon |
| [Axon Multiclamp 700B](https://biomed-labx.com/products/axon-multiclamp-700b/) | Axon |
| MPC-200 | Sutter instrument |

## Quantification and statistical analysis.

All experiments were performed and analyzed with the experimenter blind to experimental condition whenever possible. For statistical analyses, Student’s t-test, one-way analysis of variance (ANOVA), or two-way ANOVA was used as appropriate. For comparisons involving all pairwise differences among multiple groups, Tukey’s multiple-comparisons test was used. For planned comparisons following ANOVA, Bonferroni’s multiple-comparisons test was used. Repeated-measures ANOVA was applied when data were collected from the same animals across multiple conditions or time windows. The value of n represents the number of mice, cells, fields, or trials, as indicated in the figure legends. Detailed information on statistical analyses is provided in the figure legends and Supplementary Table S4. For reproducibility, each experiment was independently repeated at least three times with similar results.

1.	Huo, L. et al. Brain circuits for retching-like behavior. *Natl Sci. Rev.* **11**, nwad256 (2024).

2.	Wickersham, I. R., Finke, S., Conzelmann, K. K. & Callaway, E. M. Retrograde neuronal tracing with a deletion-mutant rabies virus. *Nat. Methods* **4**, 47–49 (2007).

3.	Zheng, G. X. Y. et al. Massively parallel digital transcriptional profiling of single cells. *Nat. Commun.* **8**, 14049 (2017).

4.	Hao, Y. et al. Integrated analysis of multimodal single-cell data. *Cell* **184**, 3573–3587.e29 (2021).

5.	McGinnis, C. S., Murrow, L. M. & Gartner, Z. J. DoubletFinder: Doublet Detection in Single-Cell RNA Sequencing Data Using Artificial Nearest Neighbors. *Cell Syst.* **8**, 329–337.e4 (2019).

6.	Korsunsky, I. et al. Fast, sensitive and accurate integration of single-cell data with Harmony. *Nat. Methods* **16**, 1289–1296 (2019).

7.	Zhou, Y. et al. Metascape provides a biologist-oriented resource for the analysis of systems-level datasets. *Nat. Commun.* **10**, 1523 (2019).

8.	Chen, T.-W. et al. Ultrasensitive fluorescent proteins for imaging neuronal activity. *Nature* **499**, 295–300 (2013).

9.	Ji, G. et al. Cognitive impairment in pain through amygdala-driven prefrontal cortical deactivation. *J. Neurosci.* **30**, 5451–5464 (2010).

10.	Wang, G. Q. et al. Deactivation of excitatory neurons in the prelimbic cortex via Cdk5 promotes pain sensation and anxiety. *Nat. Commun.* **6**, 7660 (2015).

11.	Xie, Z. et al. Mechanically evoked defensive attack is controlled by GABAergic neurons in the anterior hypothalamic nucleus. *Nat. Neurosci.* **25**, 72–85 (2022).
