# Supplementary material for: A Molecularly Defined Midbrain Circuit Mechanism for Sound‐Driven Defensive Behavior and Anxiety
Source: Adv Sci (Weinh). 2026 Sep 27:e77983. Online ahead of print. doi: 10.1002/advs.77983 (PMC13616374; doi:10.1002/advs.77983)
Supplement: Supplementary file 4 [file ADVS-9999-e77983-s009.docx]

**Contents:**

Supplementary Table 1 Information of mouse and reagents: Page 2-4

Supplementary Table 2 Summary of all experimental designs: Page 5-7

Supplementary Table 3 Summary of Cell Counting Strategy: Page 8

Supplementary Table 4 Summary of statistical analyses: Page 9-19

| **Supplementary Table 1 Information of mouse lines and reagents** | | |
| --- | --- | --- |
| **Mouse Lines** | | |
| FosCreER mice | JAX Mice | Stock No: 021882 |
| Vglut2-IRES-Cre | JAX Mice | Stock No: 016963 |
| Vgat-IRES-Cre | JAX Mice | Stock No: 028862 |
| Cbln2-IRES-Cre | JAX Mice | Stock No: 032949 |
| Cck*-*IRES-Cre | JAX Mice | Stock No: 012706 |
| **Antibodies** | | |
| Rabbit Polyclonal Anti-Glutamate | Sigma | G6642 |
| Rabbit Polyclonal Anti-GABA | Sigma | A2052 |
| Rabbit polyclonal anti-NeuN | Abcam | ab177487 |
| Rabbit polyclonal anti-EGFP | Abcam | ab290 |
| Chicken polyclonal anti-EGFP | Abcam | ab13970 |
| Chicken polyclonal anti-mCherry | Abcam | ab205402 |
| Rabbit monoclonal Anti-Fos | Cell Signaling Technology | 2250 |
| Goat anti-Rabbit IgG (H+L) Alexa 488 | Thermo Fisher Scientific | A11034 |
| Goat anti-Chicken IgG (H+L) Alexa 488 | Thermo Fisher Scientific | A11001 |
| Goat anti-Rabbit IgG (H+L) Alexa 546 | Thermo Fisher Scientific | A11010 |
| Goat anti-Chicken IgY (H+L) Alexa 546 | Thermo Fisher Scientific | A11040 |
| Goat anti-Mouse IgG (H+L) Alexa 546 | Thermo Fisher Scientific | A11030 |
| **Chemicals, peptides, and recombinant proteins** | | |
| D-AP5 | Tocris | 0106 |
| CNQX | Tocris | 0190 |
| Picrotoxin | Tocris | 1128 |
| TTX | Tocris | 1078 |
| 4-AP | Tocris | 0940 |
| DAPI | Solarbio | C0065 |
| Clozapine N-oxide | ENZO | BML-NS105 |
| Co-Detection Antibody Diluent | ACD | 323160 |
| Co-Detection Target Retrieval solution | ACD | 323166 |
| RNAscope Probe-Mm-Cbln2 | ACD | 428551 |
| **Bacterial and viral strains** | | |
| AAV2/9-EF1α-DIO-hChR2-mCherry | TaiToll | N/A |
| AAV2/9-EF1α-flex-taCasp3-TEVp-WPRE-hGH polyA | BrainVTA | N/A |
| AAV2/9-EF1α-DIO-mCherry | BrainVTA | N/A |
| AAV2/9-EF1α-DIO-RV-G | BrainVTA | N/A |
| RV-EnVA-DG-dsRed | BrainVTA | N/A |
| AAV2/9-EF1α-DIO-H2B-EGFP | BrainVTA | N/A |
| AAV2/8-hSyn-DIO-mGFP-T2A-Synaptophysin-mRuby-WPRE-hGH polyA | BrainVTA | N/A |
| AAV2/9-CAG-DIO-GCaMp6s | BrainVTA | N/A |
| AAV2/retro-EF1α-DIO-flp | BrainVTA | N/A |
| AAV2/9-EF1α-fDIO--taCasp3-TEVp-WPRE-hGH polyA | BrainVTA | N/A |
| AAV2/9-EF1α-fDIO-EGFP | BrainVTA | N/A |
| AAV2/retro-EF1α-DIO-GCaMp6s | BrainVTA | N/A |
| AAV2/9-mCaMKIIα-H2B-EGFP | BrainVTA | N/A |
| AAV2/9-mCaMKIIα-Cre | BrainVTA | N/A |
| AAV2/9-mCaMKIIα-hChR2-mCherry | BrainVTA | N/A |
| AAV2/1-DIO-Flp | BrainVTA | N/A |
| AAV2/9-EF1α-fDIO-mCherry | BrainVTA | N/A |
| rAAV-hSyn-fDIO-hM4D(Gi)-mCherry | BrainVTA | N/A |
| AAV-EF1α-DIO-H2B-tdTomato-WPREs | BrainVTA | N/A |
| AAV2/9-H1-shRNA(mCBLN2)-hSyn- EGFP-WPRE-SV40pA | TaiToll | N/A |
| AAV2/9-H1-shRNA(ffLuciferase)-hSyn-EGFP-WPRE-pA | TaiToll | N/A |
| **Primer sequences** | | |
| **Gene** | **Primer** | **Sequence (5′–3′)** |
| *Cbln2* | Forward | AACCGCACCATGACCATCTAT |
|  | Reverse | GCAAGATCAAAGTGGTTGCCAAT |
| *Gapdh* | Forward | CCCAGCAAGGACACTGAGCAA |
|  | Reverse | TTATGGGGGTCTGGGATGGAAA |

| **Supplementary Table 2 Summary of all experimental designs** | | | | |
| --- | --- | --- | --- | --- |
| **Figures** | **Aims** | **Mouse lines** | **AAV injection & optical fiber implantation** | **Type of data** |
| Fig.1a-1q  Fig.S1a-1d | Quantitative analysis of defensive behavior induced by 17–20 kHz stimulus | WT | No AAV injection. No optical fiber implantation | Behavior  Pupil size  ECG  Breathing |
| Fig.S1e-1h  Fig.S2a | Identify nuclei and neuronal cell types associated with sound-evoked defensive behavior | WT | No AAV injection. No optical fiber implantation | Histology |
| Fig.2a-2g  Fig.S2b-2e | FosTRAP2 strategy for analyzing the role of ICx in sound-evoked defensive behavior in mice | Fos-2A-CreERT2 | AAV2/9-DIO-ChR2-mCherry into ICx Optical fiber above ICx | Behavior  Histology |
| Fig.2h-2l  Fig.S2j-2o | Activation of glutamatergic neurons in the auditory pathway | Vglut2-IRES-Cre  WT | AAV2/9-DIO-ChR2-mCherry into ICx/CN/SO/MGB, Optical fiber above ICx/CN/SO/MGB;  AAV-CaMKIIα-ChR2-mCherry into ACx, Optical fiber above ACx | Behavior  Histology |
| Fig.S2f-2i | Activation of GABAergic ICx neurons | Vgat-IRES-Cre | AAV2/9-DIO-ChR2-mCherry or AV2/9-DIO-mCherry injected into the ICx, Optical fiber implanted above the ICx | Behavior  Histology |
| Fig.2m-2u  Fig.S3a-3d | Ablation of glutamatergic neurons of ICx | Vglut2-IRES-Cre | AAV2/9-DIO-taCasp3 or AAV2/9-DIO-EGFP injected into the ICx | Behavior  Pupil size  ECG  Breathing  Histology |
| Fig.S3e-3h | Ablation of glutamatergic neurons of ACx | WT | AAV-CaMKIIα-Cre and AAV2/9-DIO-taCasp3 or AAV2/9-DIO-EGFP injected into ACx | Behavior  Histology |
| Fig.S3i-3m | Fiber photometry recording from Vglut2+ ICx neurons | Vglut2-IRES-Cre | AAV2/9-DIO-GCaMp6s injected into the ICx and optical fiber implanted above the ICx | Fiber photometry  Histology |
| Fig.3a-3g | Single-nucleus RNA sequencing and gene expression analyses | WT | No AAV injection. No optical fiber implantation | Single-nucleus RNA Sequencing & analyses |
| Fig.3h-3l | Colocalization of Vglut2+ or Vgat+ neurons and Cbln2 mRNA in ICx; Colocalization of Cck^+^ neurons and Cbln2 mRNA in ICx | Vglut2-IRES-Cre  Vgat-IRES-Cre  Cbln2-IRES-Cre  Cck-IRES-Cre | AAV-DIO-H2B-EGFP injected into the ICx | Histology |
| Fig.4a-4m  Fig.S4a-4f | Optogenetic activation of Cbln2+ ICx neurons | Cbln2-IRES-Cre | AAV2/9-DIO-ChR2-mCherry or AV2/9-DIO-mCherry injected into the ICx, Optical fiber implanted above the ICx | Behavior  Pupil size  ECG  Breathing  Histology |
| Fig.4c-4d, 4g. Fig.S4g-4o | Optogenetic activation of Cck+ ICx neurons | Cck-IRES-Cre |  |  |
| Fig.4e-4f | Cbln2+ ICx neurons electrophysiological properties | Cbln2-IRES-Cre | AAV-DIO-EGFP injected into the ICx | Slice physiology |
| Fig.4n-4w  Fig.S5a-5d | Ablation of Cbln2+ neurons of ICx | Cbln2-IRES-Cre | AAV2/9-DIO-taCasp3 or AAV2/9-DIO-EGFP injected into the ICx | Behavior  Pupil size  ECG  Breathing  Histology |
| Fig.S5e-5r | Ablation of Cck+ neurons of ICx | Cck-IRES-Cre |  |  |
| Fig.S6a-6j | *Cbln2* knockdown in the ICx | WT | AAV9-H1-ShRNA(NC)-hsyn-EGFP or AAV9-H1-ShRNA(*Cbln2*)-hsyn-EGFP | Behavior  Pupil size  ECG  Breathing  Histology |
| Fig.S7a-7d | Fiber photometry recording from Cck+ ICx neurons | Cck-IRES-Cre | AAV2/9-DIO-GCaMp6s injected into the ICx and optical fiber implanted above the ICx | Fiber photometry  Histology |
| Fig.5a-5d | Fiber photometry recording from Cbln2+ ICx neurons | Cbln2-IRES-Cre | AAV2/9-DIO-GCaMp6s injected into the ICx and optical fiber implanted above the ICx | Fiber photometry  Two-photon imaging  Single-unit recording with optrode  Histology |
| Fig.5a-5d | Endoscope Ca imaging of Cbln2+ ICx neurons | Cbln2-IRES-Cre | AAV2/9-DIO-GCaMp6s injected into the ICx and cover glass above the ICx |  |
| Fig.5i-5o  Fig.S8a-8e | Single-unit recording of Cbln2+ ICx neurons | Cbln2-IRES-Cre | AAV2/9-DIO-ChR2-mCherry injected into the ICx, |  |
| Fig.S9a-9f | Anterograde tracing downstream pathway of Cbln2+ or Cck+ IC neurons | Cbln2-IRES-Cre  Cck-IRES-Cre | AAV-DIO-mGFP-T2A-Synaptophysin-mRuby | Histology |
| Fig.6a-l  Fig.S10a-10h | Activation of IC-dPAG, IC-ATm, IC-SPF, and IC-CnF pathways, and postsynaptic currents recorded from dPAG EGFP+ glutamatergic neurons | Cbln2-IRES-Cre | AAV2/9-DIO-ChR2-mCherry or AV2/9-DIO-mCherry injected into the ICx, AAV-CaMkIIα-H2B-EGFP injected into the dPAG. Optical fiber implanted above the dPAG, ATm, SPF, or CnF | Behavior  Pupil size  ECG  Breathing  Slice physiology |
| Fig.6m-6p | Chronic optogenetic activation of ICx-dPAG pathway | Cbln2-IRES-Cre | AAV2/9-DIO-ChR2-mCherry or AV2/9-DIO-mCherry injected into the ICx, Optical fiber implanted above the dPAG | Behavior |
| Fig.S10i | ICx-dPAG-AHN anterograde tracing | Vglut2-IRES-Cre | AAV2/1-DIO-flp injected into the ICx, AAV-fDIO-mCherry injected into the dPAG; | Histology |
| Fig.6q-6y  Fig.S10j-10m | Ablation of the ICx-dPAG pathway | Cbln2-IRES-Cre | AAV2/Retro-DIO-flp injected into the dPAG, AAV2/9-fDIO-taCasp3 or AAV2/9-fDIO-EGFP injected into the ICx | Behavior  Pupil size  ECG  Breathing  Histology |
| Fig.S10n-10s | Recording Cbln2^+^ neuronal activity in the ICx-dPAG pathway | Cbln2-IRES-Cre | AAV2/retro-DIO-GCaMP6s injected into the dPAG and optical fiber implanted above the ICx | Fiber photometry  Histology |
| Fig.S11a-11d | Retrograde tracing of Cbln2+ ICx-dPAG pathway neurons | Cbln2-IRES-Cre | AAV-DIO-EGFP-T2A-TVA  & AAV-DIO-RVG injected into the ICx, RV-EvnA-DG-DsRed injected into the dPAG | Histology |
| Fig.S11e-11h | Ablation of the CN-ICx pathway | Vglut2-IRES-Cre | AAV2/Retro-DIO-flp injected into the ICx, AAV2/9-fDIO-taCasp3 or AAV2/9-fDIO-EGFP injected into the CN | Behavior  Histology |
| Fig.S12a-12f | *Cbln2* knockdown in the ICx, and postsynaptic currents recorded from ICx ^Cbln2+^–dPAG EGFP+ glutamatergic neurons | Cbln2-IRES-Cre | AAV2/9-DIO-ChR2-mCherry, AAV9-H1-ShRNA(NC)-hsyn-EGFP or AAV9-H1-ShRNA(*Cbln2*)-hsyn-EGFP injected into the ICx; AAV-CaMkIIα-H2B-EGFP injected into the dPAG | Slice physiology |
| Fig.S12g-12i | *Cbln2* knockdown in the ICx, and postsynaptic currents recorded from CN^Glu^–Cbln2+ ICx neurons | Cbln2-IRES-Cre | AAV-CaMkIIα-ChR2-mCherry injected into the CN; AAV2/9-DIO-H2B-tdTomato, AAV9-H1-ShRNA(NC)-hsyn-EGFP or AAV9-H1-ShRNA(*Cbln2*)-hsyn-EGFP injected into the ICx | Slice physiology |
| Fig.7a-1e | Repeated 17–20 kHz sound exposure induces anxiety-like behaviors | WT | No AAV injection. No optical fiber implantation | Behavior |
| Fig.7f-1m | Chemogenetic inhibition of the Cbln2+ ICx–dPAG pathway | Cbln2-IRES-Cre | AAV2/Retro-DIO-flp injected into the dPAG, AAV2/9-fDIO-hM4Di-mCherry or AAV2/9-fDIO-mCherry injected into the ICx | Behavior |

| **Supplementary Table 3 Summary of Cell Counting Strategy** | | | |
| --- | --- | --- | --- |
| Brain region | ICx | dPAG | The whole brain |
| Section type | Coronal section (12 or 40 μm) | Coronal section (40 μm) | Coronal section (40 μm) |
| Section Range | Bregma (-4.90 to -5.4) | Bregma (-3.8 to -4.6) | The whole brain |
| Total collection | Approximately 36 or 11 sections | Approximately 20 sections | Approximately 300 sections |
| Sampling | To sample 1 section every 5 or 3 sections to get 7 or 4 sections evenly spaced by 60 μm | To sample 1 section every 5 sections to get 4 sections evenly spaced by 200 μm | To sample 1 section every 5 sections to get 50 sections evenly spaced by the whole brain |

| **Supplementary Table 4. Summary of statistical analyses** | | | |
| --- | --- | --- | --- |
| Figure | Sample size（n） | Statistical test | P values |
| 1f | 1–4 kHz: n = 18 mice  17–20 kHz: n = 18 mice  33–36 kHz: n = 18 mice  57–60 kHz: n = 18 mice | Two-way ANOVA with Tukey’s post hoc test | Peak locomotion speed (cm/s): During: 17–20 kHz vs. 1–4 kHz: P < 0.0001 **** During: 17–20 kHz vs. 33–36 kHz: P < 0.0001 **** During: 17–20 kHz vs. 57–60 kHz: P < 0.0001 **** |
| 1g | 50 dB: n = 14 mice  60 dB: n = 14 mice  70 dB: n = 14 mice  80 dB: n = 14 mice  90 dB: n = 14 mice | Two-way ANOVA with Tukey’s post hoc test | Peak locomotion speed (cm/s): During: 50 dB vs. 70 dB: P < 0.0001 **** During: 50 dB vs. 80 dB: P < 0.0001 **** During: 50 dB vs. 90 dB: P < 0.0001 **** During: 60 dB vs. 70 dB: P < 0.0001 **** During: 60 dB vs. 80 dB: P < 0.0001 **** During: 60 dB vs. 90 dB: P < 0.0001 **** During: 70 dB vs. 80 dB: P < 0.01 ** |
| 1q | Pre-test: n = 8 mice  Conditioning: n = 8 mice  Post-test: n = 8 mice | One-way ANOVA with Tukey’s post hoc test | Preference time (%): Pre-test vs. Conditioning: P < 0.0001 ****; Pre-test vs. Post-test: P < 0.001 ***; Conditioning vs. Post-test: P < 0.01 ** |
| 2e | 33–36 kHz-TRAP: n = 8 mice  17–20 kHz-TRAP: n = 8 mice | Two-way ANOVA with Bonferroni’s post hoc test | Peak locomotion speed (cm/s):; During: 33–36 kHz-TRAP vs. 17–20 kHz-TRAP: P < 0.0001 **** |
| 2f | 33–36 kHz-TRAP: n = 8 mice  17–20 kHz-TRAP: n = 8 mice | Two-way ANOVA with Bonferroni’s post hoc test | Time in conditioned chamber (%):; 17–20 kHz-TRAP: Pre-test vs. Post-test: P < 0.01 ** |
| 2g | 33–36 kHz-TRAP: n = 5 mice  17–20 kHz-TRAP: n = 5 mice | Two-way ANOVA with Bonferroni’s post hoc test | % Freezing: Training Context: 33–36 kHz-TRAP vs. 17–20 kHz-TRAP: P < 0.05 *; Tone: 33–36 kHz-TRAP vs. 17–20 kHz-TRAP: P < 0.001 *** |
| 2l | ICx^glu^-mCherry: n = 8 mice  ICx^glu^-ChR2: n = 5 mice  CN^glu^-ChR2: n = 7 mice  SO^glu^-ChR2: n = 6 mice  MGB^glu^-ChR2: n = 8 mice  ACx^glu^-ChR2: n = 8 mice | Two-way ANOVA with Tukey’s post hoc test | Peak locomotion speed (cm/s): During: ICx^glu^-mCherry vs. ICx^glu^-ChR2: P < 0.0001 **** During: ICx^glu^-ChR2 vs. CN^glu^-ChR2: P < 0.0001 **** During: ICx^glu^-ChR2 vs. SO^glu^-ChR2: P < 0.0001 **** During: ICx^glu^-ChR2 vs. MGB^glu^-ChR2: P < 0.0001 **** During: ICx^glu^-ChR2 vs. ACx^glu^-ChR2: P < 0.0001 **** |
| 2n | Vglut2-EGFP: n = 6 mice  Vglut2-taCasp3: n = 6 mice | Student t-test | Cell counts in ICx: Vglut2-taCasp3 vs. Vglut2-EGFP: P < 0.0001 **** |
| 2p | Vglut2-EGFP: n = 8 mice  Vglut2-taCasp3: n = 8 mice | Two-way ANOVA with Bonferroni’s post hoc test | Peak locomotion speed (cm/s):; During: Vglut2-EGFP vs. Vglut2-taCasp3: P < 0.0001 **** |
| 2s | Vglut2-EGFP: n = 9 trials  Vglut2-taCasp3: n = 9 trials | Two-way ANOVA with Bonferroni’s post hoc test | Normalized pupil size:; During: Vglut2-EGFP vs. Vglut2-taCasp3: P < 0.0001 **** After: Vglut2-EGFP vs. Vglut2-taCasp3: P < 0.0001 **** |
| 4d | Cbln2-mCherry: n = 6 mice  Cck-ChR2: n = 4 mice  Cbln2-ChR2: n = 7 mice | Two-way ANOVA with Tukey’s post hoc test | Peak locomotion speed (cm/s):; 10 mW: Cbln2-ChR2 vs. Cbln2-mCherry: P < 0.01 **; Cbln2-ChR2 vs. Cck-ChR2: P < 0.01 ** 20 mW: Cbln2-ChR2 vs. Cbln2-mCherry: P < 0.0001 ****; Cbln2-ChR2 vs. Cck-ChR2: P < 0.0001 **** 40 mW: Cbln2-ChR2 vs. Cbln2-mCherry: P < 0.0001 ****; Cbln2-ChR2 vs. Cck-ChR2: P < 0.0001 **** |
| 4g | Cbln2-mCherry: n = 5 mice  Cck-ChR2: n = 4 mice  Cbln2-ChR2: n = 7 mice | Two-way ANOVA with Bonferroni’s post hoc test | Time in conditioned chamber (%):; Cbln2-ChR2: Pre-test vs. Post-test: P < 0.001 *** |
| 4h | Cbln2-mCherry: n = 5 mice  Cbln2-ChR2: n = 7 mice | Two-way ANOVA with Bonferroni’s post hoc test | % Freezing:; Training Context: Cbln2-ChR2 vs. Cbln2-mCherry: P < 0.01 ** Tone: Cbln2-ChR2 vs. Cbln2-mCherry: P < 0.0001 **** |
| 4k | Cbln2-mCherry: n = 9 trials  Cbln2-ChR2: n = 12 trials | Two-way ANOVA with Bonferroni’s post hoc test | Normalized pupil size:; Laser: Cbln2-ChR2 vs. Cbln2-mCherry: P < 0.0001 **** Post: Cbln2-ChR2 vs. Cbln2-mCherry: P < 0.0001 **** |
| 4p | Cbln2-EGFP: n = 6 mice  Cbln2-taCasp3: n = 6 mice | Student t-test | Cell counts in ICx: Cbln2-taCasp3 vs. Cbln2-EGFP: P < 0.0001 **** |
| 4r | Cbln2-EGFP: n = 7 mice  Cbln2-taCasp3: n = 7 mice | Two-way ANOVA with Bonferroni’s post hoc test | Peak locomotion speed (cm/s):; During: Cbln2-taCasp3 vs. Cbln2-EGFP: P < 0.0001 **** |
| 4u | Cbln2-EGFP: n = 9 trials  Cbln2-taCasp3: n = 9 trials | Two-way ANOVA with Bonferroni’s post hoc test | Normalized pupil size:; During: Cbln2-taCasp3 vs. Cbln2-EGFP: P < 0.0001 **** After: Cbln2-taCasp3 vs. Cbln2-EGFP: P < 0.0001 **** |
| 5c right | Cbln2-Cre: n = 6 mice  1–4 kHz: n = 39 trials  17–20 kHz: n = 47 trials  33–36 kHz: n = 56 trials  57–60 kHz: n = 52 trials | One-way ANOVA with Tukey’s post hoc test | Peak ∆F/F (%): 17–20 kHz vs. 1–4 kHz: P < 0.0001 ****; 17–20 kHz vs. 33–36 kHz: P < 0.0001 ****; 17–20 kHz vs. 57–60 kHz: P < 0.0001 **** |
| 5n | Cbln2-Cre: n = 4 mice  1–4 kHz: n = 23 units  17–20 kHz: n = 23 units  33–36 kHz: n = 23 units  57–60 kHz: n = 23 units | Two-way ANOVA with Bonferroni’s post hoc test | Mean firing frequency (Hz): During: 17–20 kHz vs. 1–4 kHz: P < 0.0001 ****; During: 17–20 kHz vs. 33–36 kHz: P < 0.0001 ****; During: 17–20 kHz vs. 57–60 kHz: P < 0.0001 ****; After: 17–20 kHz vs. 1–4 kHz: P < 0.01 **; After: 17–20 kHz vs. 33–36 kHz: P < 0.01 **; After: 17–20 kHz vs. 57–60 kHz: P < 0.01 ** |
| 5o | Cbln2-Cre: n = 4 mice  1–4 kHz: n = 12 units  17–20 kHz: n = 12 units  33–36 kHz: n = 12 units  57–60 kHz: n = 12 units | Two-way ANOVA with Bonferroni’s post hoc test | Mean firing frequency (Hz): During: 80 dB vs. 60 dB : P < 0.0001 ****; During: 80 dB vs. 70 dB : P < 0.0001 ****; During: 70 dB vs. 60 dB : P < 0.0001 ****; After: 80 dB vs. 60 dB : P < 0.05 * |
| 6d | Cbln2-mCherry IC-dPAG: n = 6 mice  Cbln2-ChR2 IC-dPAG: n = 6 mice  Cbln2-ChR2 IC-SPF: n = 7 mice  Cbln2-ChR2 IC-ATm: n = 8 mice  Cbln2-ChR2 IC-CnF: n = 8 mice | Two-way ANOVA with Tukey’s post hoc test | Peak locomotion speed (cm/s):; 10 mW: Cbln2-ChR2 IC-dPAG vs. Cbln2-mCherry IC-dPAG: P < 0.0001 ****; Cbln2-ChR2 IC-dPAG vs. Cbln2-ChR2 IC-SPF: P < 0.01 **; Cbln2-ChR2 IC-dPAG vs. Cbln2-ChR2 IC-ATm: P < 0.0001 ****; Cbln2-ChR2 IC-dPAG vs. Cbln2-ChR2 IC-CnF: P < 0.0001 ****; 20 mW: Cbln2-ChR2 IC-dPAG vs. Cbln2-mCherry IC-dPAG: P < 0.0001 ****; Cbln2-ChR2 IC-dPAG vs. Cbln2-ChR2 IC-SPF: P < 0.0001 ****; Cbln2-ChR2 IC-dPAG vs. Cbln2-ChR2 IC-ATm: P < 0.0001 ****; Cbln2-ChR2 IC-dPAG vs. Cbln2-ChR2 IC-CnF: P < 0.0001 ****; 40 mW: Cbln2-ChR2 IC-dPAG vs. Cbln2-mCherry IC-dPAG: P < 0.0001 ****; Cbln2-ChR2 IC-dPAG vs. Cbln2-ChR2 IC-SPF: P < 0.0001 ****; Cbln2-ChR2 IC-dPAG vs. Cbln2-ChR2 IC-ATm: P < 0.0001 ****; Cbln2-ChR2 IC-dPAG vs. Cbln2-ChR2 IC-CnF: P < 0.0001 ****; |
| 6g | Cbln2-mCherry IC-dPAG: n = 9 trials  Cbln2-ChR2 IC-dPAG: n = 12 trials | Two-way ANOVA with Bonferroni’s post hoc test | Normalized pupil size:; Laser: Cbln2-ChR2 IC-dPAG vs. Cbln2-mCherry IC-dPAG: P < 0.0001 **** Post: Cbln2-ChR2 IC-dPAG vs. Cbln2-mCherry IC-dPAG: P < 0.0001 **** |
| 6k right | Before: n = 10 cells  PTX: n = 10 cells  APV/CNQX: n = 10 cells | One-way repeated-measures ANOVA followed by Tukey’s post hoc test | Amplitude of PSC (nA): Before vs. APV/CNQX: P < 0.001 *** Before vs. PTX: P > 0.05 n.s |
| 6l | CaMKIIα-EGFP-: n = 8 cells  CaMKIIα-EGFP+: n = 10 cells | Student t-test | Amplitude of EPSC (pA):; CaMKIIα-EGFP- vs. CaMKIIα-EGFP+: P < 0.001 *** |
| 6n | Cbln2-mCherry IC-dPAG: n = 12 mice  Cbln2-ChR2 IC-dPAG: n = 12 mice | Student t-test | Open field test: Total distance (cm):; Cbln2-ChR2 IC-dPAG vs. Cbln2-mCherry IC-dPAG: P = 0.6114 n.s; Center duration (s):; Cbln2-ChR2 IC-dPAG vs. Cbln2-mCherry IC-dPAG: P < 0.001 ***; Center entries:; Cbln2-ChR2 IC-dPAG vs. Cbln2-mCherry IC-dPAG: P < 0.05 * |
| 6p | Cbln2-mCherry IC-dPAG: n = 12 mice  Cbln2-ChR2 IC-dPAG: n = 12 mice | Student t-test | Time in the open arm (s):; Cbln2-ChR2 IC-dPAG vs. Cbln2-mCherry IC-dPAG: P < 0.001 *** |
| 6r | Cbln2-EGFP: n = 6 mice  Cbln2-taCasp3: n = 6 mice | Student t-test | Cell counts in ICx: Cbln2-taCasp3 vs. Cbln2-EGFP: P < 0.001 *** |
| 6t | Cbln2-EGFP: n = 8 mice  Cbln2-taCasp3: n = 8 mice | Two-way ANOVA with Bonferroni’s post hoc test | Peak locomotion speed (cm/s):; During: Cbln2-taCasp3 vs. Cbln2-EGFP: P < 0.0001 **** |
| 6w | Cbln2-EGFP: n = 9 trials  Cbln2-taCasp3: n = 9 trials | Two-way ANOVA with Bonferroni’s post hoc test | Normalized pupil size:; During: Cbln2-taCasp3 vs. Cbln2-EGFP: P < 0.0001 **** After: Cbln2-taCasp3 vs. Cbln2-EGFP: P < 0.0001 **** |
| 7c | Silent: n = 9 mice  33–36 kHz: n = 9 mice  17–20 kHz: n = 9 mice | One-way ANOVA with Tukey’s post hoc test | Total distance:  Silent vs. 17–20 kHz: P = 0.563 n.s  33–36 kHz vs. 17–20 kHz: P = 0.788 n.s  Center duration(s):  Silent vs. 17–20 kHz: P < 0.05 *  33–36 kHz vs. 17–20 kHz: P < 0.05 *  Center entries:  Silent vs. 17–20 kHz: P < 0.05 *  33–36 kHz vs. 17–20 kHz: P < 0.05 * |
| 7e | Silent: n = 9 mice  33–36 kHz: n = 9 mice  17–20 kHz: n = 9 mice | One-way ANOVA with Tukey’s post hoc test | Time in the open arm (s):  Silent vs. 17–20 kHz: P < 0.0001 ****  33–36 kHz vs. 17–20 kHz: P < 0.0001 **** |
| 7i | Co/mCherry+: 8 fields from 4 mice  Co/ hM4Di+: 8 fields from 4 mice | Student t-test | Colocalization / %:  Co/mCherry+ vs. Co/ hM4Di+: P < 0.0001 **** |
| 7k | Silent+mCherry: n = 5 mice  Silent+hM4Di: n = 5 mice  17–20 kHz+mCherry: n = 7 mice  17–20 kHz+ hM4Di: n = 7 mice | Two-way ANOVA with Bonferroni’s post hoc test | Total distance:  Silent+mCherry vs. Silent+hM4Di: P > 0.9999 n.s  17–20 kHz+mCherry vs. 17–20 kHz+ hM4Di: P = 0.6964 n.s  Center duration(s):  Silent+mCherry vs. Silent+hM4Di: P > 0.9999 n.s  17–20 kHz+mCherry vs. 17–20 kHz+ hM4Di: P < 0.05 *  Center entries:  Silent+mCherry vs. Silent+hM4Di: P > 0.9999 n.s  17–20 kHz+mCherry vs. 17–20 kHz+ hM4Di: P < 0.05 * |
| 7m | Silent+mCherry: n = 5 mice  Silent+hM4Di: n = 5 mice  17–20 kHz+mCherry: n = 7 mice  17–20 kHz+ hM4Di: n = 7 mice | Two-way ANOVA with Bonferroni’s post hoc test | Time in the open arm (s):  Silent+mCherry vs. Silent+hM4Di: P > 0.9999 n.s  17–20 kHz+mCherry vs. 17–20 kHz+ hM4Di: P < 0.05 * |
| S1b | 1–4 kHz: n = 15 trials  17–20 kHz: n = 15 trials  33–36 kHz: n = 15 trials  57–60 kHz: n = 15 trials | One-way ANOVA with Tukey’s post hoc test | Maximal normalized pupil size: During: 17–20 kHz vs. 1–4 kHz: P < 0.001 *** During: 17–20 kHz vs. 33–36 kHz: P < 0.05 * During: 17–20 kHz vs. 57–60 kHz: P < 0.01 ** |
| S1c | 1–4 kHz: n = 15 trials  17–20 kHz: n = 15 trials  33–36 kHz: n = 15 trials  57–60 kHz: n = 15 trials | One-way ANOVA with Tukey’s post hoc test | Maximal heart rate (beats/s): During: 17–20 kHz vs. 1–4 kHz: P < 0.0001 **** During: 17–20 kHz vs. 33–36 kHz: P < 0.0001 **** During: 17–20 kHz vs. 57–60 kHz: P < 0.0001 **** |
| S1d | 1–4 kHz: n = 15 trials  17–20 kHz: n = 15 trials  33–36 kHz: n = 15 trials  57–60 kHz: n = 15 trials | One-way ANOVA with Tukey’s post hoc test | Maximal breathing rate (beats/s): During: 17–20 kHz vs. 1–4 kHz: P < 0.0001 **** During: 17–20 kHz vs. 33–36 kHz: P < 0.0001 **** During: 17–20 kHz vs. 57–60 kHz: P < 0.0001 **** |
| S1g | Silent: n = 8 fields from 4 mice  17–20 kHz: n = 10 fields from 5 mice | Student t-test | Fos-positive cell numbers: IC: 17–20 kHz vs. Silent: P < 0.0001 ****; CN: 17–20 kHz vs. Silent: P < 0.0001 ****; SO: 17–20 kHz vs. Silent: P < 0.01 **; MGB: 17–20 kHz vs. Silent: P < 0.05 *; ACx: 17–20 kHz vs. Silent: P < 0.05 * |
| S1h | 17–20 kHz: n = 6 mice per group | Student t-test | Co-labeled for glutamate/GABA and c-Fos: IC: GABA+c-Fos/c-Fos vs. Glutamate+c-Fos/c-Fos: P < 0.0001 ****; CN: GABA+c-Fos/c-Fos vs. Glutamate+c-Fos/c-Fos: P < 0.001 ***; SO: GABA+c-Fos/c-Fos vs. Glutamate+c-Fos/c-Fos: P < 0.0001 ****; MGB: GABA+c-Fos/c-Fos vs. Glutamate+c-Fos/c-Fos: P < 0.0001 ****; ACx: GABA+c-Fos/c-Fos vs. Glutamate+c-Fos/c-Fos: P < 0.0001 **** |
| S2a | 17–20 kHz: 9 fields from 5 mice  33–36 kHz: 9 fields from 5 mice | Two-way ANOVA with Bonferroni’s post hoc test | Fos-positive cell numbers: IC: 17–20 kHz vs. 33–36 kHz: P < 0.01 **; ICx: 17–20 kHz vs. 33–36 kHz: P < 0.0001 ****; CIC: 17–20 kHz vs. 33–36 kHz: P < 0.0001 **** |
| S2e | 33–36 kHz-TRAP CIC: n = 8 mice  33–36 kHz-TRAP ICx: n = 8 mice  17–20 kHz-TRAP ICx: n = 8 mice | Two-way ANOVA with Bonferroni’s post hoc test | Peak locomotion speed (cm/s):; During: 33–36 kHz-TRAP CIC vs. 17–20 kHz-TRAP ICx: P < 0.0001 **** 33–36 kHz-TRAP ICx vs. 17–20 kHz-TRAP ICx: P < 0.0001 **** 33–36 kHz-TRAP CIC vs. 33–36 kHz-TRAP ICx: P > 0.9999 n.s |
| S2i | Vgat-mCherry: n = 6 mice  Vgat-ChR2: n = 6 mice | Two-way ANOVA with Bonferroni’s post hoc test | Locomotion speed (cm/s):; Before: Vgat-ChR2 vs. Vgat-mCherry: P > 0.9999 n.s During: Vgat-ChR2 vs. Vgat-mCherry: P = 0.5323 n.s After: Vgat-ChR2 vs. Vgat-mCherry: P > 0.9999 n.s |
| S2n | Vglut2-mCherry: n = 5 mice  Vglut2-ChR2: n = 5 mice | Two-way ANOVA with Bonferroni’s post hoc test | Time in conditioned chamber (%):; Vglut2-ChR2: Pre-test vs. Post-test: P < 0.001 *** |
| S2o | Vglut2-mCherry: n = 7 mice  Vglut2-ChR2: n = 7 mice | Two-way ANOVA with Bonferroni’s post hoc test | % Freezing:; Training Context: Vglut2-ChR2 vs. Vglut2-mCherry: P < 0.0001 **** Tone: Vglut2-ChR2 vs. Vglut2-mCherry: P < 0.0001 **** |
| S3b | Vglut2-EGFP: n = 9 trials  Vglut2-taCasp3: n = 9 trials | Two-way ANOVA with Tukey’s post hoc test | Maximal heart rate (beats/s): Vglut2-EGFP: Before vs. During: P < 0.0001 ****; Before vs. After: P < 0.0001 ****; During vs. After: P < 0.05 *; Vglut2-taCasp3: Before vs. During: P = 0.7923 n.s; Before vs. After: P = 0.2433 n.s; During vs. After: P = 0.0682 n.s |
| S3c | Vglut2-EGFP: n = 9 trials  Vglut2-taCasp3: n = 9 trials | Two-way ANOVA with Tukey’s post hoc test | Maximal breathing rate (beats/s):; Vglut2-EGFP: Before vs. During: P < 0.0001 ****; Before vs. After: P < 0.0001 ****; During vs. After: P = 0.5974 n.s;; Vglut2-taCasp3: Before vs. During: P = 0.7181 n.s; Before vs. After: P = 0.1971 n.s; During vs. After: P = 0.5974 n.s; |
| S3d | Vglut2-EGFP: n = 4 mice  Vglut2-taCasp3: n = 4 mice | Two-way ANOVA with Bonferroni’s post hoc test | Preference time (%):; Pre-test: Vglut2-taCasp3 vs. Vglut2-EGFP: P > 0.9999 n.s; Conditioning: Vglut2-taCasp3 vs. Vglut2-EGFP: P < 0.001 ***; Post-test: Vglut2-taCasp3 vs. Vglut2-EGFP: P < 0.001 *** |
| S3f | CaMKIIα-EGFP: n = 6 mice  CaMKIIα-taCasp3: n = 6 mice | Student t-test | Cell counts in ICx: CaMKIIα-taCasp3 vs. CaMKIIα-EGFP: P < 0.01 ** |
| S3g | CaMKIIα-EGFP: n = 7 mice  CaMKIIα-taCasp3: n = 7 mice | Two-way ANOVA with Bonferroni’s post hoc test | locomotion speed (cm/s):; Before: CaMKIIα-taCasp3 vs. CaMKIIα-EGFP: P > 0.9999 n.s; During: CaMKIIα-taCasp3 vs. CaMKIIα-EGFP: P > 0.9999 n.s; After: CaMKIIα-taCasp3 vs. CaMKIIα-EGFP: P > 0.9999 n.s |
| S3h | CaMKIIα-EGFP: n = 7 mice  CaMKIIα-taCasp3: n = 7 mice | Two-way ANOVA with Bonferroni’s post hoc test | % Freezing:; Baseline: CaMKIIα-taCasp3 vs. CaMKIIα-EGFP:P = 0.0895 n.s; CS+: CaMKIIα-taCasp3 vs. CaMKIIα-EGFP: P < 0.001 *** |
| S3k | Vglut2-Cre: n = 5 mice  1–4 kHz: n = 26 trials  17–20 kHz: n = 30 trials  33–36 kHz: n = 32 trials  57–60 kHz: n = 28 trials | One-way ANOVA with Tukey’s post hoc test | Peak ∆F/F (%): 17–20 kHz vs. 1–4 kHz: P < 0.0001 ****; 17–20 kHz vs. 33–36 kHz: P < 0.0001 ****; 17–20 kHz vs. 57–60 kHz: P < 0.0001 **** |
| S4a | Cbln2-mCherry: n = 6 mice  Cbln2-ChR2: n = 7 mice | Two-way ANOVA with Bonferroni’s post hoc test | Peak locomotion speed (cm/s):; 5 s: Cbln2-ChR2 vs. Cbln2-mCherry: P < 0.0001 **** |
| S4b | Cbln2-mCherry: n = 6 mice  Cbln2-ChR2: n = 7 mice | Two-way ANOVA with Bonferroni’s post hoc test | Peak locomotion speed (cm/s):; 20 Hz: Cbln2-ChR2 vs. Cbln2-mCherry: P < 0.0001 **** |
| S4c | Cbln2-mCherry: n = 6 mice  Cbln2-ChR2: n = 7 mice | Two-way ANOVA with Tukey’s post hoc test | Peak locomotion speed (cm/s):; Cbln2-mCherry: 1st vs. 2nd: P = 0.9920 n.s; 1st vs. 3rd: P = 0.9875 n.s; 2nd vs. 3rd: P = 0.9601 n.s; Cbln2-ChR2: 1st vs. 2nd: P = 0.8513 n.s; 1st vs. 3rd: P = 0.9811 n.s; 2nd vs. 3rd: P = 0.9327 n.s |
| S4e | Cbln2-mCherry: n = 9 trials  Cbln2-ChR2: n = 12 trials | Two-way ANOVA with Tukey’s post hoc test | Maximal heart rate (beats/s):; (Cbln2-mCherry: Pre vs. Laser: P > 0.9999 n.s; Pre vs. Post: P > 0.9999 n.s; Laser vs. Post: P > 0.9999 n.s; Cbln2-ChR2: Pre vs. Laser: P < 0.0001 ****; Pre vs. Post: P < 0.0001 ****; Laser vs. Post: P = 0.3077 n.s) |
| S4f | Cbln2-mCherry: n = 9 trials  Cbln2-ChR2: n = 12 trials | Two-way ANOVA with Tukey’s post hoc test | Maximal breathing rate (beats/s):; (Cbln2-mCherry: Pre vs. Laser: P = 0.8811 n.s; Pre vs. Post: P = 0.9688 n.s; Laser vs. Post: P = 0.9688 n.s; Cbln2-ChR2: Pre vs. Laser: P < 0.0001 ****; Pre vs. Post: P < 0.0001 ****; Laser vs. Post: P < 0.0001 ****) |
| S4i | Cck-mCherry: n = 9 trials  Cck-ChR2: n = 9 trials | Two-way ANOVA with Bonferroni’s post hoc test | Normalized pupil size:; Pre: Cck-mCherry vs. Cck-ChR2: P > 0.9999 n.s; Laser: Cck-mCherry vs. Cck-ChR2: P > 0.9999 n.s; Post: Cck-mCherry vs. Cck-ChR2: P > 0.9999 n.s |
| S4m | Cck-mCherry: n = 9 trials  Cck-ChR2: n = 9 trials | Two-way ANOVA with Tukey’s post hoc test | Maximal heart rate (beats/s):; (Cck-mCherry: Pre vs. Laser: P = 0.5868 n.s; Pre vs. Post: P = 0.3072 n.s; Laser vs. Post: P = 0.8741 n.s; Cck-ChR2: Pre vs. Laser: P = 0.8741 n.s; Pre vs. Post: P = 0.3072 n.s; Laser vs. Post: P = 0.5868 n.s) |
| S4n | Cck-mCherry: n = 9 trials  Cck-ChR2: n = 9 trials | Two-way ANOVA with Tukey’s post hoc test | Maximal breathing rate (beats/s):; (Cck-mCherry: Pre vs. Laser: P > 0.9999 n.s; Pre vs. Post: P = 0.8870 n.s; Laser vs. Post: P = 0.8870 n.s; Cck-ChR2: Pre vs. Laser: Pre vs. Laser: P = 0.3483 n.s; Pre vs. Post: P = 0.6216 n.s; Laser vs. Post: P = 0.8870 n.s) |
| S5b | Cbln2-EGFP: n = 9 trials  Cbln2-taCasp3: n = 9 trials | Two-way ANOVA with Tukey’s post hoc test | Maximal heart rate (beats/s): Cbln2-EGFP: Before vs. During: P < 0.0001 ****; Before vs. After: P < 0.0001 ****; During vs. After: P = 0.8118 n.s; Cbln2-taCasp3: Before vs. During: P = 0.0872 n.s; Before vs. After: P = 0.9491 n.s; During vs. After: P = 0.1627 n.s |
| S5c | Cbln2-EGFP: n = 9 trials  Cbln2-taCasp3: n = 9 trials | Two-way ANOVA with Tukey’s post hoc test | Maximal breathing rate (beats/s):; Cbln2-EGFP: Before vs. During: P < 0.0001 ****; Before vs. After: P < 0.0001 ****; During vs. After: P < 0.0001 ****; Cbln2-taCasp3: Before vs. During: P = 0.7081 n.s; Before vs. After: P = 0.7081 n.s; During vs. After: P = 0.2600 n.s |
| S5d | Cbln2-EGFP: n = 7 mice  Cbln2-taCasp3: n = 7 mice | Two-way ANOVA with Bonferroni’s post hoc test | Preference time (%):; Pre-test: Cbln2-taCasp3 vs. Cbln2-EGFP: P = 0.7012 n.s; Conditioning: Cbln2-taCasp3 vs. Cbln2-EGFP: P < 0.0001 ****; Post-test: Cbln2-taCasp3 vs. Cbln2-EGFP: P < 0.0001 **** |
| S5g | Cck-EGFP: n = 6 mice  Cck-taCasp3: n = 6 mice | Student t-test | Cell counts in ICx:; Cck-taCasp3 vs. Cck-EGFP: P < 0.0001 **** |
| S5i | Cck-EGFP: n = 7 mice  Cck-taCasp3: n = 7 mice | Two-way ANOVA with Bonferroni’s post hoc test | locomotion speed (cm/s):; Before: Cck-taCasp3 vs. Cck-EGFP: P > 0.9999 n.s; During: Cck-taCasp3 vs. Cck-EGFP: P > 0.9999 n.s; After: Cck-taCasp3 vs. Cck-EGFP: P > 0.9999 n.s |
| S5l | Cck-EGFP: n = 9 trials  Cck-taCasp3: n = 9 trials | Two-way ANOVA with Bonferroni’s post hoc test | Normalized pupil size:; Before: Cck-taCasp3 vs. Cck-EGFP: P > 0.9999 n.s; During: Cck-taCasp3 vs. Cck-EGFP: P > 0.9999 n.s; After: Cck-taCasp3 vs. Cck-EGFP: P = 0.7893 n.s |
| S5p | Cck-EGFP: n = 9 trials  Cck-taCasp3: n = 9 trials | Two-way ANOVA with Tukey’s post hoc test | Maximal heart rate (beats/s): Cck-EGFP: Before vs. During: P < 0.0001 ****; Before vs. After: P < 0.0001 ****; During vs. After: P < 0.05 *; Cck-taCasp3: Before vs. During: P < 0.05 *; Before vs. After: P < 0.0001 ****; During vs. After: P < 0.01 ** |
| S5q | Cck-EGFP: n = 9 trials  Cck-taCasp3: n = 9 trials | Two-way ANOVA with Tukey’s post hoc test | Maximal breathing rate (beats/s):; Cck-EGFP: Before vs. During: P < 0.01 **; Before vs. After: P = 0.0540 n.s; During vs. After: P = 0.3334 n.s; Cck-taCasp3: Before vs. During: P < 0.0001 ****; Before vs. After: P < 0.001 ***; During vs. After: P = 0.6092 n.s |
| S5r | Cck-EGFP: n = 7 mice  Cck-taCasp3: n = 7 mice | Two-way ANOVA with Bonferroni’s post hoc test | Preference time (%):; Pre-test: Cck-taCasp3 vs. Cck-EGFP: P > 0.9999 n.s; Conditioning: Cck-taCasp3 vs. Cck-EGFP: P > 0.9999 n.s; Post-test: Cck-taCasp3 vs. Cck-EGFP: P > 0.9999 n.s; Cck-taCasp3: Pre-test vs. Conditioning: P < 0.01 **; Cck-EGFP: Pre-test vs. Conditioning: P < 0.001 *** |
| S6b | NC: n = 6 mice  Cbln2-KD: n = 6 mice | Student t-test | *Cbln2* mRNA fluorescence intensity:; Cbln2-KD vs. NC: P < 0.0001 **** |
| S6d | NC: n = 7 mice  Cbln2-KD: n = 7 mice | Two-way ANOVA with Bonferroni’s post hoc test | Peak locomotion speed (cm/s):; During: Cbln2-KD vs. NC: P < 0.0001 **** |
| S6e | NC: n = 6 mice  Cbln2-KD: n = 6 mice | Two-way ANOVA with Bonferroni’s post hoc test | Preference time (%):; Pre-test: Cbln2-KD vs. NC: P = 0.9864 n.s; Conditioning: Cbln2-KD vs. NC: P < 0.01 **; Post-test: Cbln2-KD vs. NC: P < 0.01 ** |
| S6g | NC: n = 9 trials  Cbln2-KD: n = 9 trials | Two-way ANOVA with Bonferroni’s post hoc test | Normalized pupil size:; During: Cbln2-KD vs. NC: P < 0.0001 **** After: Cbln2-KD vs. NC: P < 0.0001 **** |
| S6i | NC: n = 9 trials  Cbln2-KD: n = 9 trials | Two-way ANOVA with Bonferroni’s post hoc test | Maximal heart rate (beats/s): NC: Before vs. During: P < 0.0001 ****; Before vs. After: P < 0.0001 ****; During vs. After: P = 0.5294 n.s; Cbln2-KD: Before vs. During: P = 0.9997 n.s; Before vs. After: P = 0.5294 n.s; During vs. After: P =0.3575 n.s; |
| S6j | NC: n = 9 trials  Cbln2-KD: n = 9 trials | Two-way ANOVA with Bonferroni’s post hoc test | Maximal breathing rate (beats/s):; NC: Before vs. During: P < 0.0001 ****; Before vs. After: P < 0.0001 ****; During vs. After: P = 0.3891 n.s; Cbln2-KD: Before vs. During: P > 0.9999 n.s; Before vs. After: P > 0.9999 n.s; During vs. After: P > 0.9999 n.s; |
| S7c | Cck-Cre: n = 4 mice  1–4 kHz: n = 36 trials  17–20 kHz: n = 54 trials  33–36 kHz: n = 42 trials  57–60 kHz: n = 43 trials | One-way ANOVA with Tukey’s post hoc test | Peak ∆F/F (%): 17–20 kHz vs. 1–4 kHz: P = 0.2197 n.s; 17–20 kHz vs. 33–36 kHz: P < 0.0001 ****; 17–20 kHz vs. 57–60 kHz: P < 0.0001 ****; 33–36 kHz vs. 1–4 kHz: P < 0.0001 ****; 33–36 kHz vs. 57–60 kHz: P < 0.001 ***; 57–60 kHz vs. 1–4 kHz: P < 0.0001 **** |
| S10a | Cbln2-mCherry: n = 6 mice  Cbln2-ChR2: n = 6 mice | Two-way ANOVA with Bonferroni’s post hoc test | Peak locomotion speed (cm/s):; 2 Hz: Cbln2-ChR2 vs. Cbln2-mCherry: P < 0. 05 *; 20 Hz: Cbln2-ChR2 vs. Cbln2-mCherry: P < 0.0001 **** |
| S10b | Cbln2-mCherry: n = 6 mice  Cbln2-ChR2: n = 6 mice | Two-way ANOVA with Bonferroni’s post hoc test | Peak locomotion speed (cm/s):; 5 s: Cbln2-ChR2 vs. Cbln2-mCherry: P < 0.0001 **** |
| S10c | Cbln2-mCherry: n = 6 mice  Cbln2-ChR2: n = 6 mice | Two-way ANOVA with Tukey’s post hoc test | Peak locomotion speed (cm/s):; Cbln2-mCherry: 1st vs. 2nd: P = 0.9677 n.s; 1st vs. 3rd: P = 0.9693 n.s; 2nd vs. 3rd: P > 0.9999 n.s; Cbln2-ChR2: 1st vs. 2nd: P = 0.6361 n.s; 1st vs. 3rd: P = 0.4049 n.s; 2nd vs. 3rd: P = 0.9202 n.s |
| S10e | Cbln2-mCherry: n = 9 trials  Cbln2-ChR2: n = 12 trials | Two-way ANOVA with Tukey’s post hoc test | Maximal heart rate (beats/s):; (Cbln2-mCherry: Pre vs. Laser: P = 0.7989 n.s; Pre vs. Post: P = 0.9048 n.s; Laser vs. Post: P = 0.9753 n.s; Cbln2-ChR2: Pre vs. Laser: P < 0.0001 ****; Pre vs. Post: P < 0.0001 ****; Laser vs. Post: P = 0.5131 n.s) |
| S10f | Cbln2-mCherry: n = 9 trials  Cbln2-ChR2: n = 12 trials | Two-way ANOVA with Tukey’s post hoc test | Maximal breathing rate (beats/s):; (Cbln2-mCherry: Pre vs. Laser: P = 0.9885 n.s; Pre vs. Post: P = 0.9885 n.s; Laser vs. Post: P > 0.9999 n.s; Cbln2-ChR2: Pre vs. Laser: P < 0.0001 ****; Pre vs. Post: P < 0.0001 ****; Laser vs. Post: P < 0.0001 ****) |
| S10g | Cbln2-mCherry: n = 6 mice  Cbln2-ChR2: n = 6 mice | Two-way ANOVA with Bonferroni’s post hoc test | Time in conditioned chamber (%):; Cbln2-ChR2: Pre-test vs. Post-test: P < 0.0001 **** |
| S10h | Cbln2-mCherry: n = 6 mice  Cbln2-ChR2: n = 6 mice | Two-way ANOVA with Bonferroni’s post hoc test | % Freezing:; Training Context: Cbln2-ChR2 vs. Cbln2-mCherry: P < 0.0001 **** Tone: Cbln2-ChR2 vs. Cbln2-mCherry: P < 0.0001 **** |
| S10j | Cbln2-EGFP: n = 7 mice  Cbln2-taCasp3: n = 7 mice | Two-way ANOVA with Bonferroni’s post hoc test | Preference time (%):; Pre-test: Cbln2-taCasp3 vs. Cbln2-EGFP: P > 0.9999 n.s; Conditioning: Cbln2-taCasp3 vs. Cbln2-EGFP: P < 0.0001 ****; Post-test: Cbln2-taCasp3 vs. Cbln2-EGFP: P < 0.0001 **** |
| S10l | Cbln2-EGFP: n = 9 trials  Cbln2-taCasp3: n = 9 trials | Two-way ANOVA with Tukey’s post hoc test | Maximal heart rate (beats/s): Cbln2-EGFP: Before vs. During: P < 0.01 **; Before vs. After: P < 0.0001 ****; During vs. After: P < 0.001 ***; Cbln2-taCasp3: Before vs. During: P = 0.4659 n.s; Before vs. After: P = 0.3069 n.s; During vs. After: P = 0.0595 n.s |
| S10m | Cbln2-EGFP: n = 9 trials  Cbln2-taCasp3: n = 9 trials | Two-way ANOVA with Tukey’s post hoc test | Maximal breathing rate (beats/s):; Cbln2-EGFP: Before vs. During: P < 0.0001 ****; Before vs. After: P = 0.0853 n.s; During vs. After: P < 0.001 ***; Cbln2-taCasp3: Before vs. During: P = 0.9009 n.s; Before vs. After: P = 0.9742 n.s; During vs. After: P = 0.9742 n.s |
| S10s | Cbln2-Cre: n = 5 mice  1–4 kHz: n = 36 trials  17–20 kHz: n = 35 trials  33–36 kHz: n = 41 trials  57–60 kHz: n = 42 trials | One-way ANOVA with Tukey’s post hoc test | Peak ∆F/F (%): 17–20 kHz vs. 1–4 kHz: P < 0.0001 ****; 17–20 kHz vs. 33–36 kHz: P < 0.0001 ****; 17–20 kHz vs. 57–60 kHz: P < 0.0001 ****; 33–36 kHz vs. 1–4 kHz: P = 0.9806 n.s; 33–36 kHz vs. 57–60 kHz: P = 0.9925 n.s; 57–60 kHz vs. 1–4 kHz: P = 0.9994 n.s |
| S11f | CN-ICx Vglut2+-EGFP: n = 6 mice  CN-ICx Vglut2+-taCasp3: n = 6 mice | Student t-test | Cell counts in ICx: CN-ICx Vglut2+-EGFP vs. CN-ICx Vglut2+-taCasp3: P < 0.001 *** |
| S11h | CN-ICx Vglut2+-EGFP: n = 6 mice  CN-ICx Vglut2+-taCasp3: n = 6 mice | Two-way ANOVA with Bonferroni’s post hoc test | Peak locomotion speed (cm/s):; During: CN-ICx Vglut2+-EGFP vs. CN-ICx Vglut2+-taCasp3: P < 0.0001 **** |
| S12b | NC: n = 4 mice  Cbln2-KD: n = 4 mice | Student t-test | Cell counts in ICx:; Cbln2-KD vs. NC: P = 0.7888 n.s |
| S12c | NC: n = 4 mice  Cbln2-KD: n = 4 mice | Student t-test | Relative *Cbln2* mRNA expression:; Cbln2-KD vs. NC: P < 0.01 ** |
| S12f | NC: n = 10 cells from 4 mice  Cbln2-KD: n = 11 cells from 5 mice | Student t-test | Amplitude of EPSC (pA):; Cbln2-KD vs. NC: P < 0.01 ** |
| S12i | NC: n = 18 cells from 5 mice  Cbln2-KD: n = 13 cells from 5 mice | Student t-test | Amplitude of EPSC (pA):; Cbln2-KD vs. NC: P < 0.05 * |
